# Targeted pathway mining reveals novel modified hexapeptides from the demiguisin family

**DOI:** 10.1038/s42004-026-02250-w

**Published:** 2026-10-17

**Authors:** Tanya Decker, Marie Dayras, Lena Schweizer, Sari Rasheed, Franziska Fries, Bulisa Iova, Kasun H. Bodawatta, Knud Andreas Jønsson, Rolf Müller, Christine Beemelmanns

**Affiliations:** 1 Helmholtz Institute for Pharmaceutical Research Saarland (HIPS), Saarbrücken, Germany; 2 Université d’Orléans, CNRS, ICOA, Orléans, France; 3 German Center for Infection Research (DZIF), Partner Site Hannover-Braunschweig, Braunschweig, Germany; 4 PNG National Museum and Art Gallery, Port Moresby, Papua New Guinea; 5 Section for Molecular Ecology and Evolution, Globe Institute, University of Copenhagen, Copenhagen, Denmark; 6 Department of Bioinformatics and Genetics, Swedish Museum of Natural History, Stockholm, Sweden; 7 Pharma Science Hub, Saarland University, Saarbrücken, Germany; 8 Faculty of Medicine, Saarland University, Saarbrücken, Germany

**Keywords:** Microbiology, Metabolomics, Biosynthesis

## Abstract

Following the discovery of the piperazic acid (Piz)-containing hexapeptide demiguisin A from a toxic bird-associated *Amycolatopsis* strain, we aimed to investigate the structural diversity and bioactive potential of this natural product family. Here, we used the *dmg* biosynthetic gene cluster from *Amycolatopsis* sp. PS_44_ISF1 as a pathway mining probe to search for alternative demiguisin A producers. A global search identified only one other soil-derived *Amycolatopsis* sp. NBC_01488, encoding a highly syntenic pathway. Under feather-mimicking conditions, PS_44_ISF1 demonstrated enhanced growth and metabolite production relative to NBC_01488. Despite phylogenomic distance, both strains produced shared metabolites, while NBC_01488 yielded more abundant demiguisins, enabling the isolation of five novel derivatives characterized by two Piz-moieties with different hydroxylation patterns and glycine truncation. Two congeners displayed potent activity against the avian pathogen *Mycobacterium intracellulare* and moderate activity against *Mycobacterium tuberculosis*. These findings show that targeted pathway mining guided the expansion of the chemical diversity of Piz-modifications and demiguisin congeners with promising anti-*Mycobacterium* activity.

## Introduction

**Fig. 1:**
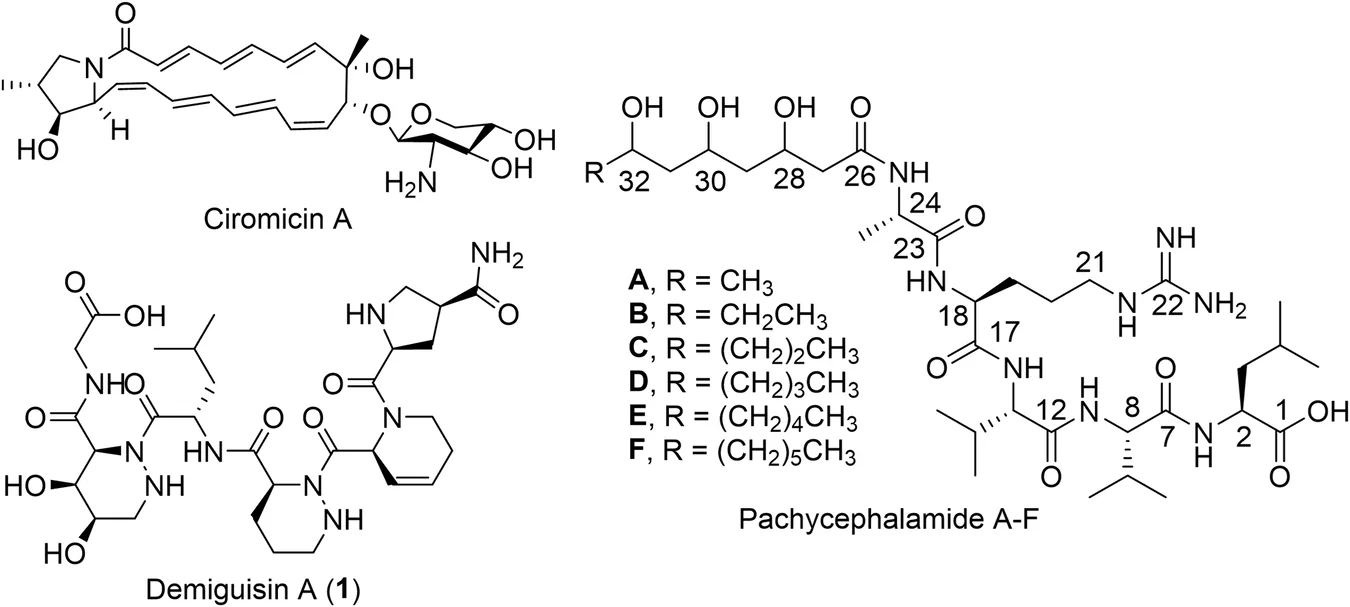
Chemical structures of natural products isolated from *Amycolatopsis* sp. PS_44_ISF1. Scaffolds include the macrolactam ciromicin A, the linear hexapeptide demiguisin (**1**), and lipopeptides pachycephalamides A-F.

Birds rely on a collection of behavioural, physiological, and chemical defences to mitigate the constant threat posed by pathogens and parasites in their environment^1^. Preen oil from the uropygial gland (UG) is a key component of avian integumentary immunity, supporting feather maintenance and waterproofing, while the UG is also known to harbour diverse microbes, including γ- and α-Pseudomonadota and Actinomycetota, that contribute to feather defense^2,3^. We recently exemplified a case of the bacterial isolate *Amycolatopsis* sp. PS_44_ISF1 derived from the UG of the toxic bird *Pachycephala schlegelii* that can directly contribute to the bird’s chemical defence system, by producing bioactive metabolites such as the antifungal macrolactam ciromicin A^3,4^, the co-secreted tensioactive lipopeptide family pachycephalamides, and a highly modified hexapeptide, named demiguisin A (**1**) (Fig. 1). The chemical structure of **1**, caught our interest, as it contains non-proteinogenic piperazic acid (hexahydropyridazine-3-carboxylic acid, Piz) residues within the sequence Gly–β,γ-OH-Piz–Leu–Piz–DHPA–γ-c-Pro (Fig. 1)^3,5^. Owing to its cyclized structure, Piz imparts significant conformational rigidity to peptide scaffolds, a property often correlated with enhanced biological activity, including anticancer, anti-infective, antiparasitic, antimetastatic, and immunosuppressive activities^5^.

Given the highly modified structural features of **1** and the complexity of its multimodular non-ribosomal peptide synthetase (NRPS)-dependent biosynthesis (referred to as *dmg*) entailing several modifying enzyme candidates^3^, we hypothesized that the structural diversity within the demiguisin compound family might be broader than originally described. To test this hypothesis, we employed a combination of targeted genome mining and targeted metabolite profiling, and identified a soil-derived *Amycolatopsis* sp. NBC_01488 as a potential producer^6^. Optimized cultivation conditions enabled the isolation and structural elucidation of new demiguisin derivatives, followed by reassessment of their biosynthesis and antibacterial activity against a panel of bacterial strains. Motivated by the possibility that these compounds may contribute to the protection of keratin-rich feather surfaces in the avian host environment, demiguisins were also assessed for their activity against feather-degrading bacteria.

## Results and discussion

### Genome mining for demiguisin-encoding biosynthetic gene clusters

**Fig. 2:**
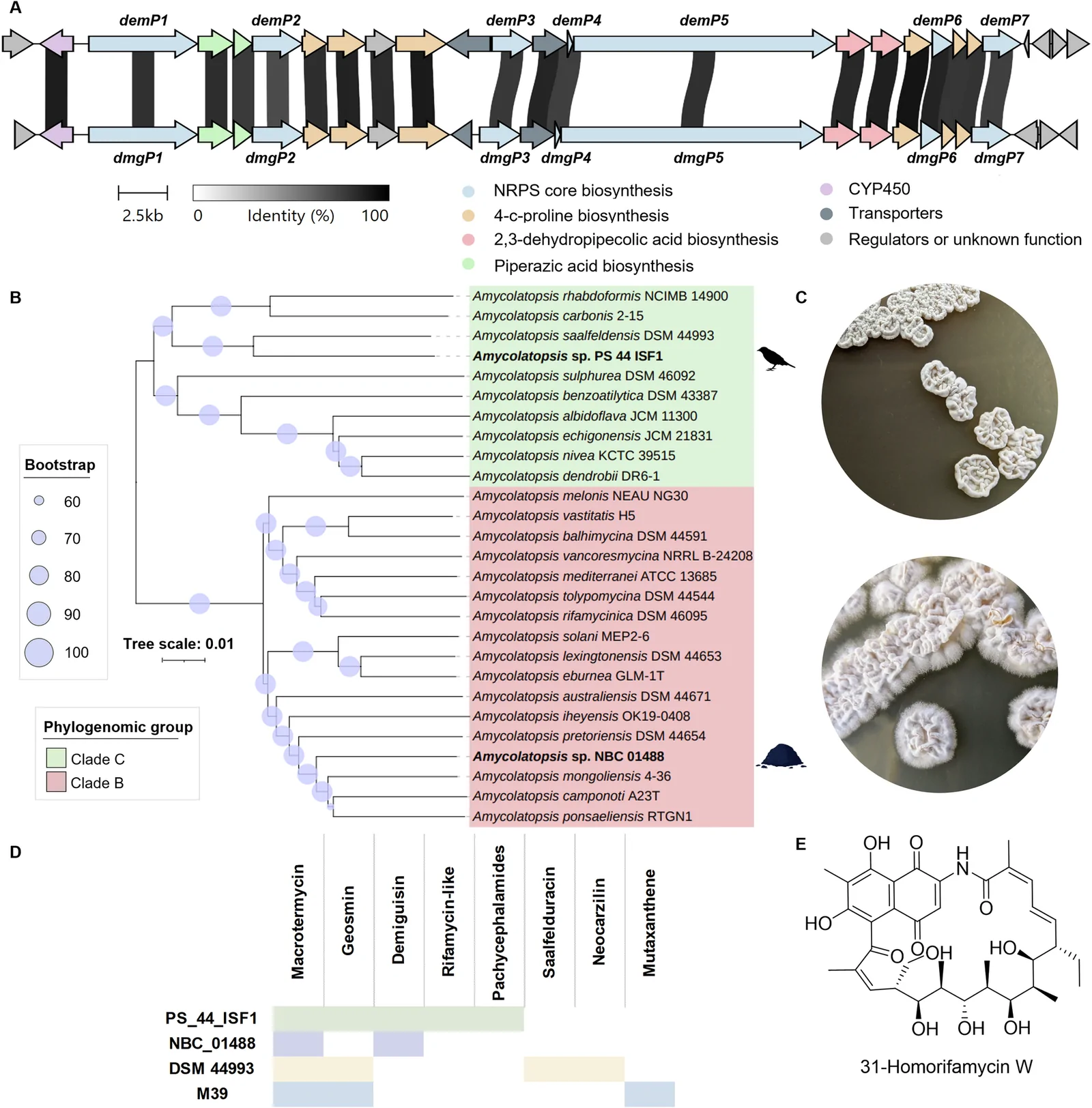
Taxonomic analysis and genome mining of *Amycolatopsis* species. **A** Clinker alignment of *dem* (NBC_01488) and *dmg* (PS_44_ISF1) gene clusters encoding demiguisin. **B** Unrooted phylogenomic tree that was generated using TYGS highlights NBC_01488 (soil-derived) and PS_44_ISF1 (bird-derived), with an illustration depicting their isolation source, including closely related species. **C** Colony morphology of *Amycolatopsis* sp. PS_44_ISF1 (top) and *Amycolatopsis* sp. NBC_01488 (bottom). **D** Distribution of known BGCs detected in *Amycolatopsis* sp. PS_44_ISF1, *Amycolatopsis* sp. NBC_01488, *A. saalfeldensis* DSM 44993, and *Amycolatopsis* sp. M39. **E** Chemical structure of 31-homorifamycin W^13^.

To identify strains potentially capable of producing demiguisins, we first queried the full *dmg* biosynthetic gene cluster (BGC) sequence (region 3.20 in PS_44_ISF1; the BGC previously verified using knockout studies to encode for demiguisin A production) using an nBLAST (NCBI) search, but no fully matching hit was deposited within the public databases^3^. Subsequently, various gene segments (i.e., section *dmgP1* to *dmgP7*, gene *dmgP5*, and gene *dmgP1*) of the *dmg* BGC from PS_44_ISF1 were individually queried, yielding only one homologous gene cluster sequence (*dmgP5*; Fig. 2A) encoded within the genome of *Amycolatopsis* sp. NBC_01488 (region 1.19 in NBC_01488, referred to as *dem*)^6^. To identify the taxonomic origin of the BGC hit, we then pursued a phylogenomic analysis of both *Amycolatopsis* strains, PS_44_ISF1 and NBC_01488, using the Type Strain Genome Server (TYGS) platform to infer genome-based relatedness. Intriguingly, both strains were found to be located within different *Amycolatopsis* phylogenomic clades (Fig. 2B)^7,8^. While the closest relative of strain PS_44_ISF1 remains *Amycolatopsis saalfeldensis* DSM 44993, *Amycolatopsis* sp. NBC_01488 forms a sister clade with *Amycolatopsis mongoliensis* 4-36 (Fig. 2B). Further TYGS analysis indicated that both strains, PS_44_ISF1 and NBC_01488, likely each represent unique species, a conclusion also supported by Whole-Genome Average Nucleotide Identity (FastANI) results (Supplementary Fig. 1), showing that their nearest known relatives exhibit ANI values below the 95% species-delineation threshold^9^.

In light of the phylogenomic distance of the encoding two strains and to further analyse the *dem* cluster region of *Amycolatopsis* sp. NBC_01488 in greater detail, we performed a comprehensive analysis of their full biosynthetic potential using antiSMASH V7.1 and used two in-house *Amycolatopsis* strains (M39 and DSM 44993) containing experimentally validated BGCs for comparison and dereplication purposes^10^. The BGC landscape was subsequently analysed using BiG-SCAPE V2.0 through comparison with characterized clusters in the MiBIG V4.0 database^11,12^. The analysis pipeline showed that several BGCs were conserved across all four strains (Supplementary Fig. 2), such as the cluster encoding for the production of the antifungal macrolactams, while clusters encoding for pachycephalamides and a rifamycin-like BGC (encoding metabolites corresponding to 31-homorifamycin W; Fig. 2E) appeared to be unique to *Amycolatopsis* sp. PS_44_ISF1 (Fig. 2D and Supplementary Fig. 2A, B)^13^. The two closely related clusters encoding for demiguisin production were present only in *Amycolatopsis* sp. PS_44_ISF1 and *Amycolatopsis* sp. NBC_01488. Detailed pairwise comparison of the *dmg* (PS_44_ISF1) and *dem* (NBC_01488) BGCs using clinker (CAGECAT webserver) revealed a high degree of synteny and sequence identity (>70%) across the core NRPS loci, particularly within the central NRPS assembly line (Fig. 2A and Supplementary Fig. 2C)^14^. However, the two BGCs did not cluster together in the BiG-SCAPE network under standard cutoffs (Supplementary Fig. 2), likely due to variability in flanking or auxiliary genes outside the core NRPS region (Supplementary Fig. 2B).

Taken together, the presence of highly similar *dmg*/*dem* BGCs in the phylogenomically distinct *Amycolatopsis* sp. PS_44_ISF1 and *Amycolatopsis* sp. NBC_01488 strains suggest a shared evolutionary history; however, whether this reflects horizontal gene transfer or vertical inheritance from a common ancestor cannot be resolved based on the current dataset.

### Evaluation of cultivation conditions on demiguisin production

**Fig. 3:**
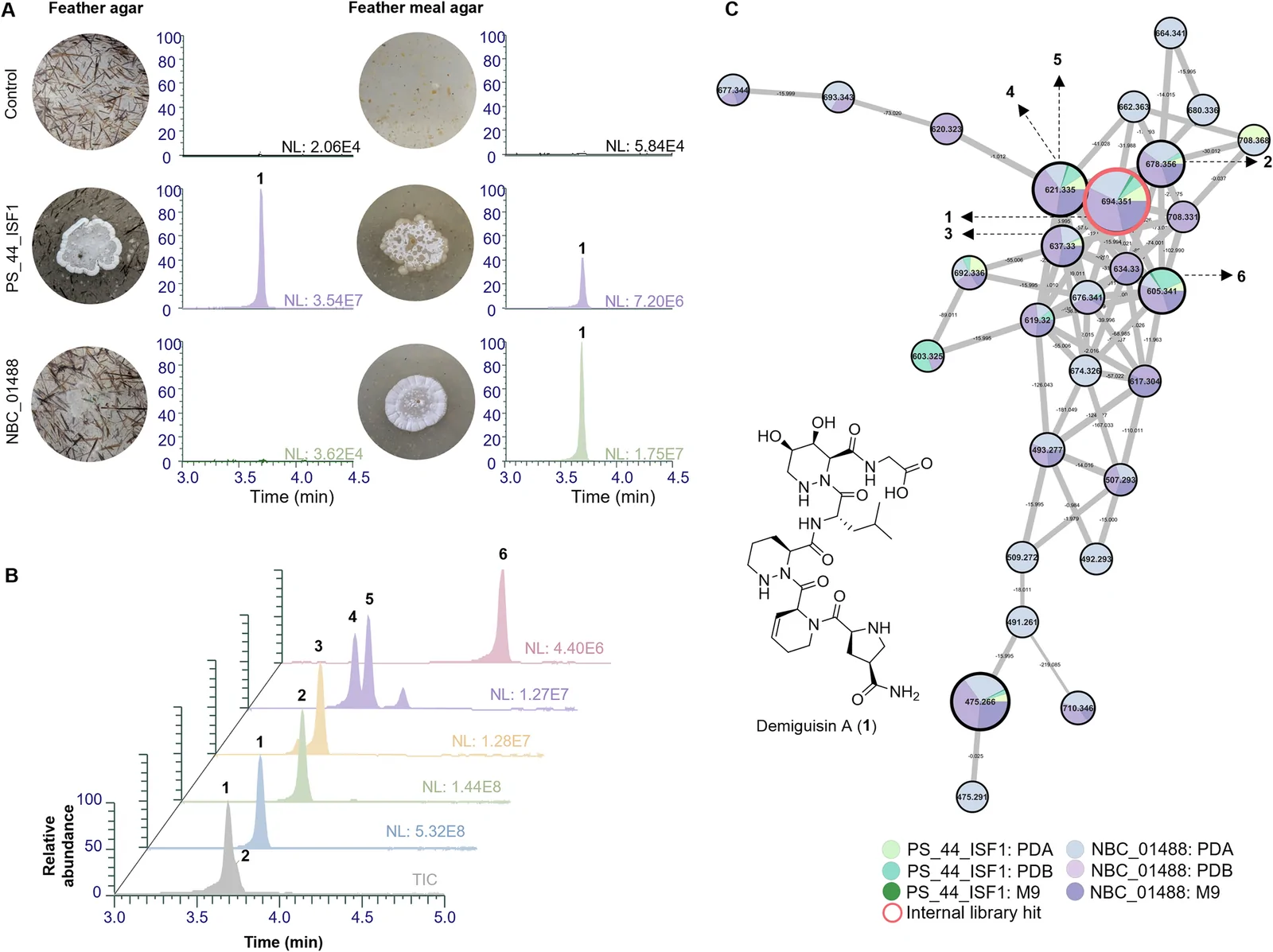
Morphological, physiological, and metabolomic analyses of *Amycolatopsis* sp. PS_44_ISF1 and NBC_01488. **A** Growth phenotypes of *Amycolatopsis* sp. PS_44_ISF1 and NBC_01488 on pheasant feather medium and feather meal medium after 14 days. **B** The bottom lane (grey) shows the most abundant metabolites (**1**–**2**) in the total ion chromatogram of NBC_01488, and the coloured lanes show extracted ion chromatograms of lower abundance compounds **3**–**6**. **C** GNPS molecular network highlighting nodes corresponding to **1** and analogues (compounds **2**–**6**) produced by *Amycolatopsis* sp. PS_44_ISF1 and *Amycolatopsis* sp. NBC_01488 after 14 days. Node colours indicate the producing strain and cultivation medium, while edge thickness reflects cosine similarity between MS/MS spectra, and node size indicates the number of spectra per node, providing an estimate of relative abundance.

With PS_44_ISF1 and NBC_01488 strains potentially capable of producing new demiguisins, due to the presence of the *dmg*/*dem* BGCs in their genomes, we then aimed to confirm production of **1** in the newly identified strain as well as potential novel derivatives of this peptide by both strains and compare their relative abundance. To evaluate growth, metabolite production, and potential differences in demiguisin-derived peptide scaffolds, both strains (*Amycolatopsis* sp. PS_44_ISF1 and *Amycolatopsis* sp. NBC_01488) were cultivated on nutrient-rich (PDB and PDA), nutrient-poor (M9), and feather-based media (FA and FMA). The use of feather-based media for cultivation purposes was intended to assess whether the bird-derived demiguisin-producing strain is adapted to avian keratinous substrates. For comparative metabolite profiling analysis of cultivation studies, solid-phase enriched metabolites were analysed by LC–HRMS/MS (Fig. 3B), and MS data dereplicated using web-based Global Natural Products Social Molecular Networking (GNPS) V1.0, SIRIUS V6.1, and the Natural Product Atlas (NPAtlas) database, and visualized in Cytoscape V3.10^15–18^.

Prior to evaluating the growth of both strains on the selected feather-based media, we first mined both genomes for keratinases, typically annotated as subtilisin-like serine proteases belonging to the S8 family^19^. As references, we used the well-characterized KerA (GenBank ID: AAB34259.1) from *Bacillus licheniformis* and KerC (GenBank ID: ABY65903.1) from *Bacillus subtilis* in a dedicated BLASTp (NCBI) searches against the genomes of PS_44_ISF1 and NBC_01488^19,20^. Indeed, results from these queries returned multiple high-confidence KerA-like and KerC-like hits annotated as S8 family serine peptidases (Supplementary Tables 5 and 6)^19^, while in both cases PS_44_ISF1 displayed significantly higher query coverage and lower E-values, consistent with full-length subtilisin-like proteases and supporting its potential for growth on keratin-rich substrates. We hypothesized that *Amycolatopsis* sp. PS_44_ISF1 is better adapted to utilize native keratin as a growth substrate.

To validate this hypothesis, we conducted a keratin degradation assay with both strains on the pre-processed FMA (with readily accessible keratin-derived nutrients) and on non-processed pheasant feathers (resembling the nutrient-limited avian feather environment dominated by recalcitrant β-keratin). This resulted in the bird-derived PS_44_ISF1 strain exhibiting 80% degradation of feathers when grown on feather broth media and 48% on feather meal broth (Supplementary Fig. 38 and Supplementary data 1). In comparison, no degradation was observed when the soil-derived NBC_01488 strain was grown on feather broth media, and 28% on feather meal broth (Supplementary Fig. 38). Moreover, growth studies clearly showed that avian-derived strain PS_44_ISF1 exhibited robust growth on feather-based substrates (FA and FMA), whereas the soil-derived strain NBC_01488 grew well on the pre-processed FMA, while it failed to grow on non-processed pheasant feathers (Fig. 3A)^19,20^. The higher keratinolytic activity of PS_44_ISF1 is thus consistent with the genomic analyses, which identified more complete KerA- and KerC-like subtilisin homologs in this strain. Metabolite extractions from these plates confirmed that NBC_01488 is capable of producing compound **1**; however, production was only detected on the pre-processed FMA substrate that supported strain growth (Fig. 3A, Supplementary Fig. 4, and Supplementary Data 2). Taken together, the enhanced keratinolytic capacity of the bird-derived PS_44_ISF1 enables efficient utilization of intact feather keratin, supporting robust growth and making this strain well suited for production of demiguisins on native feather substrates.

Motivated by the confirmation of demiguisin A (**1**) production by the newly identified NBC_01488 strain, we then conducted GNPS molecular networking to revealed a diverse repertoire of secondary metabolites produced by both strains (PS_44_ISF1 and NBC_01488) when cultivated on the five selected media types (Supplementary Fig. 3). Annotated metabolite features unique to the avian-derived *Amycolatopsis* sp. PS_44_ISF1 were identified as pachycephalamides A–F, ciromicin A, and 31-homorifamycin W, consistent with its predicted BGC repertoire (Fig. 2E and Supplementary Fig. 3)^3,4,13^. Most importantly for the focus of this study, the metabolite profile of both strains, PS_44_ISF1 and NBC_01488, shared an expansive demiguisin subnetwork, thus confirming that both strains produce novel structurally diverse demiguisin derivatives. Detailed analysis of the cluster revealed that the feature annotated as demiguisin A (**1**) (*m*/*z* 694.3519 [M + H]^+^, RT: 3.69 min) clustered together with several unannotated nodes (i.e., *m*/*z* 678.3563, 637.3299, 621.3348, 621.3378, and 605.3411 [M + H]^+^) (Fig. 3B, C and Supplementary Fig. 3), which were named demiguisins (**2**–**6**). Analysis of the production levels of molecular features **1**–**6** showed that **1** remained the predominant metabolite under all tested conditions. Production levels were substantially higher in PDA medium (**1**: 1.31 × 10^8^) than in M9 (**1**: 6.19 × 10^6^) or FA medium (**1**: 3.54 × 10^7^) and correlated with the growth behavior of the producer strain. However, the highest overall abundance of **1** was detected from the soil-derived strain NBC_01488 cultivated on PDA (Supplementary Fig. 4 and Supplementary Data 3). In contrast, the abundances of features assigned to compounds **2**–**6** remained low and varied considerably across cultivation conditions, while cultivation time strongly influenced overall feature abundance (Supplementary Fig. 4 and Supplementary data 4). Here, it needs to be noted that co-cultivation of PS_44_ISF1 with avian- and non-avian-derived strains belonging to the genus *Kocuria*, *Streptomyces*, and *Rhodococcus* did not increase overall production of demiguisins nor significantly alter peptide composition (Supplementary Fig. 5 and Supplementary data 5).

### Structural elucidation of new Piz-containing peptides

**Fig. 4:**
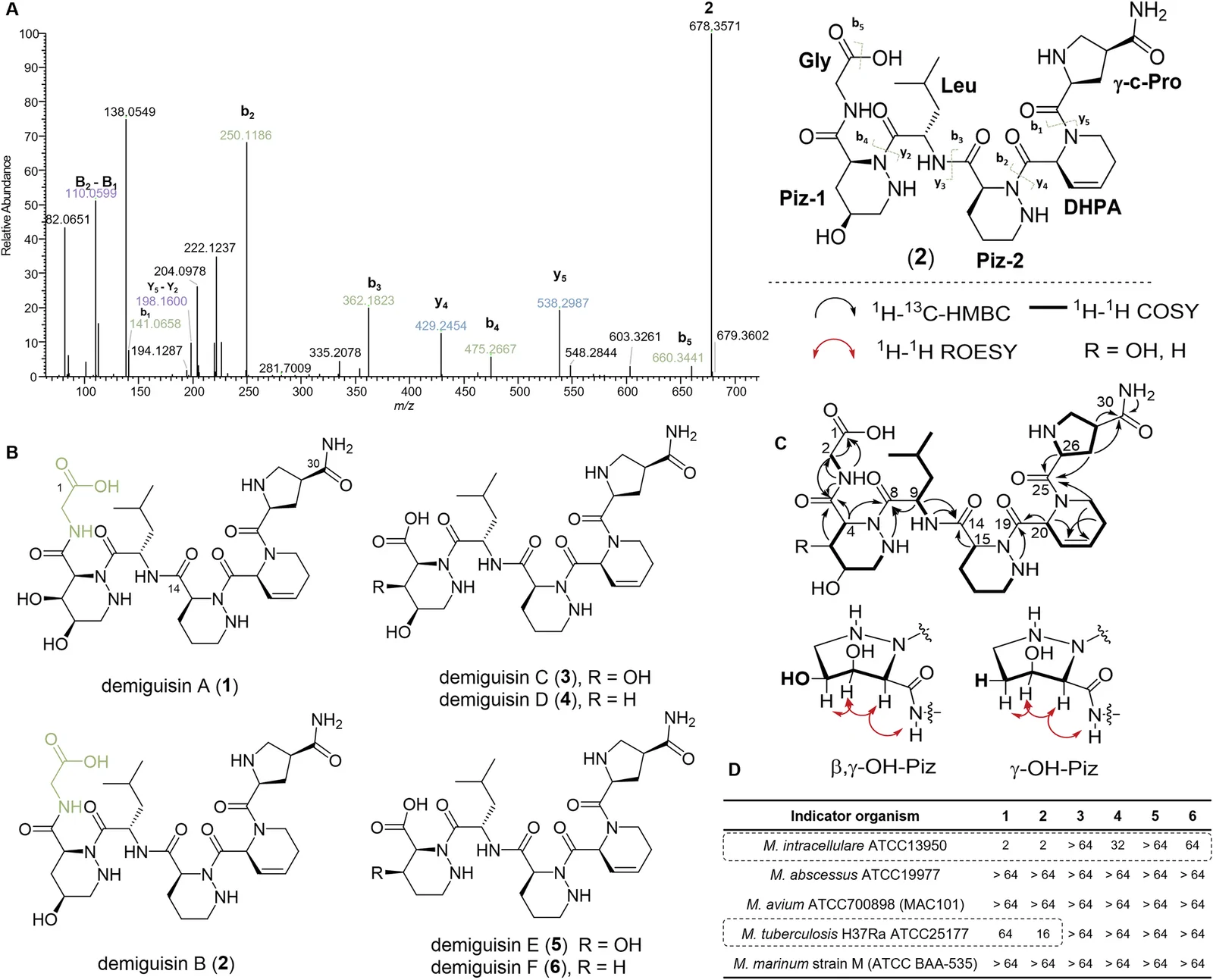
Characterization of demiguisin derivatives. **A**, **B** The MS/MS fragmentation profile of new demiguisin derivative (**2**) annotated with MS/MS fragments with matching calculated *m/z* values of the a, b, and y ions. **C** 2D-NMR correlations of demiguisin B (**2**) (^1^H-^1^H COSY: Homonuclear correlation spectroscopy; ^1^H-^13^C HMBC: Heteronuclear multiple-bond correlation spectroscopy; ^1^H-^1^H ROESY: Rotating frame Overhauser effect spectroscopy). **D** Bioactivity data as MIC values given in µg/mL.

Intrigued by the presence of yet unreported molecular ion features related to the demiguisin compound family (*m*/*z* 678.3563, 637.3299, 621.3348, 621.3378, and 605.3411 [M + H]^+^) produced by both strains, we leveraged the outcomes of the cultivation studies for upscaling the production of demiguisins using *Amycolatopsis* sp. NBC_01488 (PDA plates, 10 days). Solid-phase extraction of methanolic extracts was performed for pre-fractionation prior to UV-guided reverse-phase HPLC on a phenyl-hexyl column, and purified compounds were confirmed with LC-HRMS/MS. This resulted in the isolation of six demiguisin compounds, which included the most abundant, known derivative demiguisin A (**1**) and five new congeners. Comparative NMR analysis of **1** (*m*/*z* of 694.3517 [M+H]^+^) isolated from NBC_01488 was conducted with the NMR data of the demiguisin A (Gly–β,γ-OH-Piz–Leu–Piz–DHPA–γ-c-Pro) produced by PS_44_ISF1. For complete assignment, 1D and 2D NMR data analyses were performed in DMSO-d_6_ (Supplementary Table 7 and Supplementary Figs. 7–12) and compared to previously reported NMR data^5^, leading to the confirmation that both strains produce the same demiguisin A compound. The second most abundant congener, demiguisin B (**2**), with an *m*/*z* value of 678.3571 [M + H]^+^, had a molecular formula of C_30_H_47_N_9_O_9_ (calcd. for C_30_H_48_N_9_O_9_, 678.3575 [M + H]^+^), suggesting a difference from **1** of 16 Da characteristic of the loss of an oxygen (Supplementary Figs. 13–17 and Supplementary Table 7). The linear amino acid sequence Gly–γ-OH-Piz–Leu–Piz–DHPA–γ-c-Pro (Fig. 4), accounting for the twelve degrees of unsaturation, was assembled based on the molecular formula, diagnostic MS/MS fragmentation patterns, and corroborating HMBC and ROESY correlations from α-proton signals to amide carbonyl signals of neighbouring amino acids. The MS/MS fragmentation pattern of compound **2** showed characteristic ions indicating a single hydroxyl group on the first Piz moiety (Fig. 4). Combined 1D and 2D NMR analyses revealed a peptide scaffold containing glycine (Gly), leucine (Leu), and Piz residues, alongside signals for two oxidatively modified amino acids, including oxygenated methine and olefin correlations.

The oxygenated Piz residue adjacent to the C-terminal glycine was identified as 4-hydroxyhexahydropyridazine-3-carboxylic acid (γ-OH-Piz). This assignment was supported by a characteristic COSY-defined spin system comprising a methine proton H-4 (δ_H_ 4.77), a methylene proton H_2_-5 (δ_H_ 2.39 and 1.91), an oxygenated methine proton H-6 (δ_H_ 3.64), and a methylene proton H_2_-7 (δ_H_ 2.80 and 2.72), and key HMBC correlations from H-4 to C-3 (δ_C_ 170.8). The presence of a 3,4-dehydropipecolic acid moiety (DHPA, 3,4-dehydro-homoproline) was deduced based on an extended COSY correlation network between H-20 (δ_H_ 5.73)/H-21 (δ_H_ 5.88)/H-22 (δ_H_ 5.91)/H_2_-23 (δ_H_ 2.19 and 2.08)/H_2_-24 (δ_H_ 3.94, 3.35) defining a continuous spin system spanning from the α-methine carbon C-20 (δ_C_ 51.6) to the epsilon methylene carbon C-24 (δ_C_ 39.6). HMBC correlations of the α-proton (H-20, δ_H_ 5.73) and δ-proton (H-23b, δ_H_ 2.02) to γ-olefin carbon (C-21, δ_C_ 122.6), and from ε-proton (H-24a, δ_H_ 3.94) to β-olefin carbon (C-22, δ_C_ 126.6) aligned with the deductions. The third modified amino acid was assigned as γ-carbamoyl-proline (γ-c-Pro). A partial structure of the third modified amino acid, γ-carbamoyl-proline (γ-c-Pro). COSY correlations established proton–proton connectivity between the methine proton H-26 (δ_H_ 3.88), the β-protons H_2_-27 (δ_H_ 2.27 and 1.64), the γ-methine proton H-28 (δ_H_ 2.80), and the methylene protons H_2_-29 (δ_H_ 2.99 and 2.80). The HMBC correlations from primary amine proton signals (30-NH_2_, δ_H_ 7.35 and 6.82), β-proton (H-27a, δ_H_ 2.27), and δ-proton (H-29a, δ_H_ 2.99) to C-30 (δ_C_ 175.6) allowed to deduce the planar structure of γ-c-Pro. Overall, compound **2** differs from **1** as it contains a γ-OH-Piz instead of β,γ-OH-Piz. Demiguisin C (**3**), with *m/z* 637.3299 [M + H]^+^, exhibited a molecular formula of C_28_H_44_N_8_O_9_ (calcd. for C_28_H_45_N_8_O_9_, 637.3309 [M + H]^+^), representing a loss of C_2_H_2_N (57 Da) compared to **1**, consistent with the absence of the C-terminal glycine residue. MS/MS fragmentation analysis confirmed the lack of glycine-containing fragment ions, while retaining fragments consistent for β,γ-OH-Piz–Leu–Piz–DHPA–γ-c-Pro. The ^1^H and ^13^C NMR spectra showed the characteristic absence of the glycine signals compared to **1** and, in combination with 2D NMR spectra (Supplementary Figs. 18–21 and Supplementary Table 8), confirmed the structure of compound **3** as β,γ-OH-Piz–Leu–Piz–DHPA–γ-c-Pro (Fig. 4). Demiguisin D (**4**) and demiguisin E (**5**) (*m*/*z* 621.3348 and 621.3378 [M + H]^+^) exhibited identical molecular formulas of C_28_H_44_N_8_O_8_ (calcd. for C_28_H_45_N_8_O_8_, 621.3360 [M + H]^+^) and indistinguishable MS/MS fragmentation patterns, indicating that they are positional isomers differing only in the site of hydroxylation on the Piz-1 residue. Analysis of their 1D and 2D NMR data showed that compound **4** contains a γ-hydroxylated Piz unit (Supplementary Table 8 and Supplementary Figs. 22–25), analogous to that observed for demiguisin B (**2**). In contrast, compound **5** displayed a distinct COSY spin system consistent with β-hydroxylation of the Piz-1 residue, as evidenced by correlations between the α-CH (H-2, δ_H_ 4.46) and an oxygenated β-CH signal (H-3, δ_H_ 4.21) (Supplementary Table 8 and Supplementary Figs. 26–28). Accordingly, compounds **4** and **5** were elucidated as γ-OH-Piz–Leu–Piz–DHPA–γ-c-Pro and β-OH-Piz–Leu–Piz–DHPA–γ-c-Pro, respectively (Fig. 4). Demiguisin F (**6**) (*m/z* 605.3400 [M + H]^+^) showed a molecular formula of C_28_H_44_N_8_O_7_ (calcd. for C_28_H_46_N_8_O_7_, 605.3411 [M + H]^+^) corresponding to a further loss of one oxygen atom relative to compounds **4** and **5**. Comparison of its ¹H NMR and 2D NMR data with those of demiguisin C-E (**3**–**5**) revealed the absence of oxygenated methine signals associated with the Piz-1 residue (Supplementary Table 8 and Supplementary Figs. 29–31), while all remaining amino acid motifs were conserved. Accordingly, compound **6** was assigned as Piz–Leu–Piz–DHPA–γ-c-Pro (Fig. 4). Given the structural similarity and the high homology of the overall BGC as well as the absence of epimerases within the NRPS BGC responsible for compounds **1**–**6** (vide infra), and the conserved chemical shifts and coupling patterns observed for the Leu and Piz residues across all compounds, the same L-stereochemistry was inferred for all derivatives.

### Bioactivity Assessment

As pre-screening of culture extracts against feather-degrading bacteria (*Kocuria*, *Curtobacterium*) isolated from the same avian host species showed that primarily fractions containing rifamycin-like metabolites exhibited inhibitory activity, with minor activity observed from the demiguisin fractions (Supplementary Fig. 39), we expanded our antimicrobial testing of the isolated Piz-containing compounds specifically to a panel of bacterial and fungal test strains, with a particular focus on *Mycobacterium* spp. (*M. tuberculosis*, *M. marinum*, *M. abscessus*, *M. avium*, and *M. intracellulare*; full panel in Supplementary Table 10). This focus was motivated by the prominent antimycobacterial activity reported for many Piz-containing natural products, including activity against drug-resistant *Mycobacterium* species, but also by the limited quantity of isolated derivatives **2**–**6**. As expected, demiguisin A showed no activity against Gram-positive or Gram-negative test strains, including efflux-deficient and hyperpermeable *E. coli* strains (MIC > 64 µg/mL, Supplementary Table 10); however, antimycobacterial activity was observed against *M. tuberculosis* H37Ra, with compound **2** exhibiting moderate activity (MIC 16 µg/mL), whereas compound **1** showed only weak activity (MIC 64 µg/mL). In addition, compounds **1** and **2** displayed potent activity against *M. intracellulare* (MIC 2 µg/mL), which is a member of the *M. avium* complex (MAC) that has been reported to cause avian tuberculosis, although this disease is predominantly associated with *M. avium* subsp. *avium*^21^. Derivatives **4** and **6**, which lack the Gly residue, showed weaker activity against this strain with MIC values of 32 µg/mL and 64 µg/mL, respectively, while compounds **3** and **5** were inactive (MIC > 64 µg/mL) (Fig. 4D). Furthermore, it was noted that none of the compounds exhibited cytotoxicity toward the human hepatic cell line HepG2 (IC₅₀ >37 µg/mL; Supplementary Table 10). These observations suggest that the terminal Gly residue may play an important role in maintaining the bioactive conformation or physicochemical properties required for antimycobacterial activity. At the same time, the absence of detectable cytotoxicity toward the HepG2 cell line indicates that the Piz residue itself is unlikely to be responsible for the cytotoxic effects often associated with other Piz-containing natural products.

### Comparative analysis of demiguisin biosynthesis

**Fig. 5:**
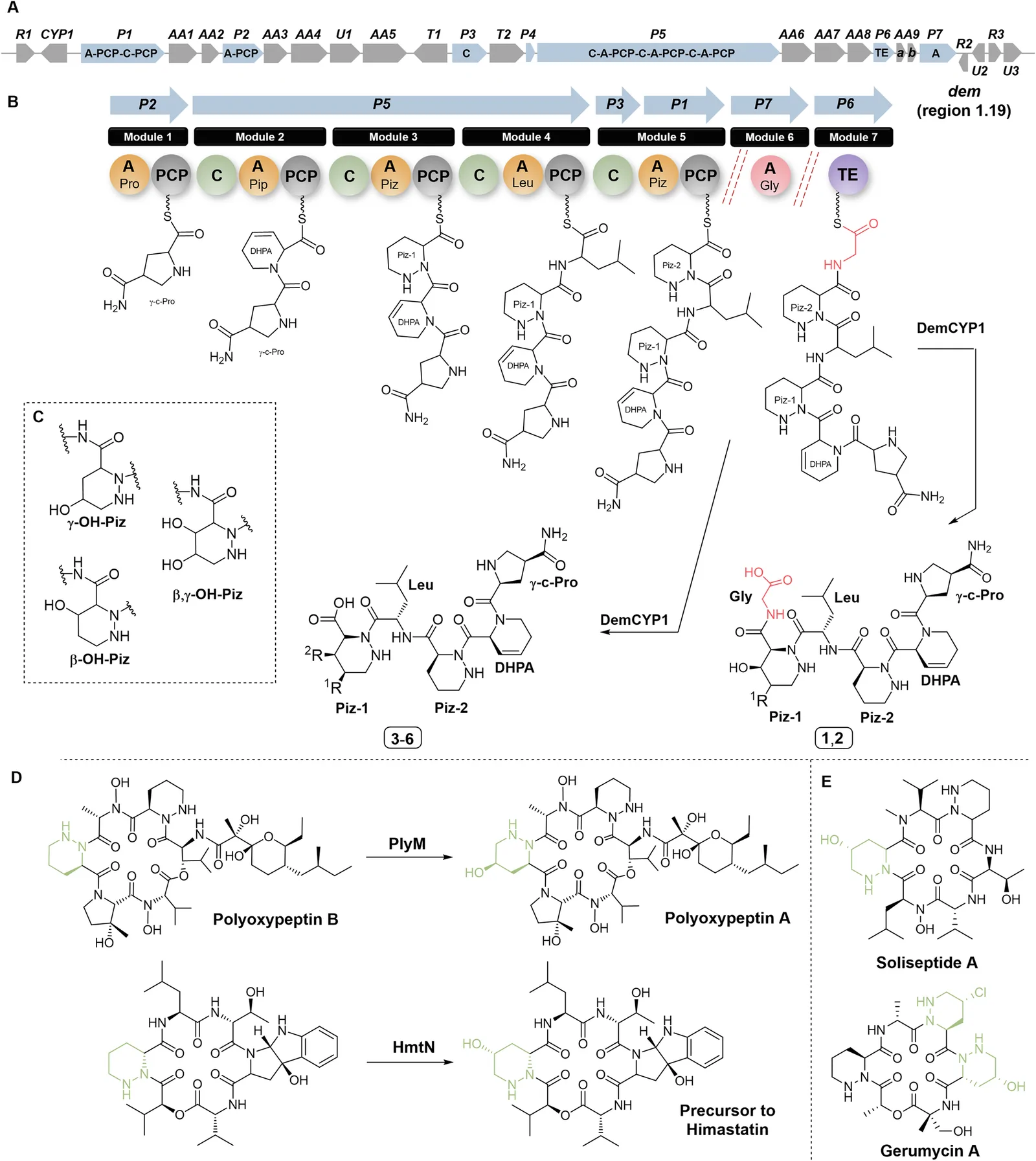
Retrobiosynthetic analysis of *dem* BGC. **A** Organization of the *dem* BGC and **B** NRPS assembly line for the production of a proposed demiguisin derivatives, highlighting the variable incorporation of Gly and **C** hydroxylation of the Piz-1 moiety. **D** Two examples of cytochrome P450 monooxygenases (PlyM and HmtN) that catalyse the hydroxylation of piperazic acid unit within a microbial natural product as a post-translation modification^5^. **E** Two examples of microbial natural products with modifications (i.e., chlorination or hydroxylation) catalysed by unknown enzymes. Abbreviations: PCP – peptidyl carrier protein, C – condensation domain, A – adenylation domain, TE – thioesterase, Pip – piperazine, Piz – piperazic acid, Gly – glycine, Pro – proline, Lys – lysine, Leu – leucine, DHPA – 3,4-dehydro-homoproline.

Building on previous studies, which verified the BGC responsible for demiguisin A production by knockout studies, we then compared the architecture of the BGC homolog *dem* (NBC_01488) and *dmg* (PS_44_ISF1) in greater detail to deduce the origin of the amino acid variations (presence/absence of Gly and hydroxylation of the Piz-1). The identified *dem* region spanned approximately 39.2 kb encoding 26 open reading frames (*demR1-U3*), including seven NRPS-encoding genes (*demP1-P7*) (with protein similarities for each gene matched to the MiBIG database; Supplementary Table 11). Nine genes encoding for enzymes responsible for amino acid biosynthesis and tailoring modifications (*demAA1-AA9*, *demCYP1*), two genes encoding for transport (*demT1-T2*), three genes for regulatory (*demR1-R3*) functions, and three with unknown function (*demU1-U3*) (Fig. 5A). Most importantly, four core NRPS genes *demP1*, *demP2*, *demP5*, and *demP7* were tentatively assigned to encode for six adenylation (A) domains, designated to incorporate the amino acids into the growing peptide chain and mirroring what has previously been reported for *dmg* in PS_44_ISF1. The substrate specificities of the six A domains in NBC_01488 were examined using the PARAS and PRISM 4 webtools^22,23^, with PARAS predicting a peptide sequence of Pro–Pro–Pro–Pro–Leu–Gly, while PRISM 4 predicted a peptide sequence of Pro–Pip–Pro–Pip–Phe–Gly (Supplementary Table 12). Similar predictions were observed for *dmg* (PS_44_ISF1) (Supplementary Table 12). Thus, despite minor discrepancies, the overall chemical features were consistent with the bioinformatic predictions.

However, the origin of the observed glycine-specific truncation remained unclear as both biosynthetic pathways (*dem* and *dmg*) harboured a glycine-specific A-domain (*demP7* or *dmgP7*) within the NRPS. Thus, we speculated that either dedicated peptidases or the unusual phenomenon of modular skipping^24^ might be the cause of the glycine-specific truncation (Fig. 5B). However, neither peptidases nor other similarly acting enzymes were detectable within the broader BGC boundaries that would support the selective cleavage and inaction hypothesis. Instead, both the *demP7* and *dmgP7* loading modules lack a condensation (C) domain, which may impair glycine incorporation and promote premature chain release or module bypass, consistent with the skipping model, and result in shunt products. Skipped modules often result in premature release of peptide intermediates from upstream modules due to disrupted downstream interactions or terminal-like release activity. This phenomenon has been observed in both engineered and naturally occurring non-linear NRPS systems and is influenced by enzyme dynamics and interdomain interactions^25,26^.

Amongst the different structural features of demiguisins, the observed variation in the site-specific mono- and di-hydroxylation pattern of the first Piz residue (Piz-1, Fig. 5C) represented the most prominent structural differences among the isolated derivatives. Current evidence suggests that unmodified Piz is first incorporated by dedicated NRPS adenylation domains and subsequently modified after peptide release from the assembly line^5^. However, modifications occurring on NRPS-bound intermediates cannot be excluded at present. While no enzymes that selectively catalyse Piz β-hydroxylation have, to our knowledge, been reported or experimentally characterized, γ-hydroxylation of Piz residues by P450 enzymes has been proposed for structurally related NRPS-derived peptide scaffolds. For example, Ju and co-workers identified the cytochrome P450 monooxygenase HmtN, which catalyses the γ-hydroxylation of piperazic acid following peptide assembly (Fig. 5D)^27^. Similarly, the biosynthesis of polyoxypeptin A entails the cytochrome P450 monooxygenase PlyM, which is hypothesized to catalyse the conversion of polyoxypeptin B to the γ-OH-Piz-containing product (Fig. 5D)^28^. Thus, we performed BLASTp analyses and multiple sequence alignments of four CYP450 enzymes (HmtN, PlyA, DemCYP450, and DmgCYP450) to explore potential sequence–function relationships. Despite their common annotation as P450 monooxygenases, HmtN as well as PlyA exhibited only low sequence identity and similarity to DemCYP450 and DmgCYP450 (Supplementary Table 13 and Supplementary Fig. 40). Nevertheless, their annotation and conserved genomic positioning within the respective BGCs support the hypothesis that DemCYP450 and DmgCYP450 catalyse site-specific hydroxylation of Piz. Alternatively, we speculate that the BGC-encompassing LLM-class flavin-dependent oxidoreductases DemAA3 and DmgAA1 may mediate oxidative modifications of the Piz moiety or perform related auxiliary redox reactions related to precursor biosynthesis. Overall, while the experimental validation of such non-canonical pathways poses inherent challenges, future whole or partial pathway cloning followed by domain and enzyme inactivation and peptide ejection assays could help distinguish whether the truncated derivatives arise from premature TE-mediated release or bona fide skipping of the terminal NRPS module, and may allow to determine the precise roles, timing of action, and substrate specificities of oxidative enzymes.

## Conclusion

Since the first discovery of the unusual amino acid Piz by Hassall and co-workers in 1959, about 140 Piz-containing natural products have been reported, predominantly from actinomycetes^5^. This study highlights how genome-guided exploration of related biosynthetic pathways can uncover both improved producer strains and expanded structural diversity. We used the *dmg* BGC from *Amycolatopsis* sp. PS_44_ISF1 as a probe to guide the identification of a new *Amycolatopsis* sp. NBC_01488 strain encoding a *dem* BGC capable of producing demiguisin, while functional analysis of degradative capacities identified habitat-specific growth conditions and covered that the newly identified soil-derived strain shows higher production titers than the avian isolate. Exploration of different cultivation conditions enabled the detection and isolation of Piz-containing demiguisin derivatives featuring glycine truncation and position-specific hydroxylation of only one of the two Piz moieties. Despite pronounced phylogenomic divergence between *Amycolatopsis* sp. PS_44_ISF1 and *Amycolatopsis* sp. NBC_01488, the distinct colony morphologies, and their isolation from geographically distant and ecologically distinct environments, the high conservation of the *dmg* and *dem* BGCs suggests strong selective pressure to maintain this biosynthetic pathway. The herein reported expansion of the demiguisin family further expands the chemical diversity of Piz-modifications and the overall Piz-containing peptides with promising antimycobacterial activity.

## Materials and methods

### Chemicals and equipment

All media ingredients, reagents, and chemicals were purchased as follows: Methanol (Th. Geyer, Renningen); water for analytical and preparative HPLC (VWR International GmbH, Dresden); formic acid (VWR International GmbH, Dresden); acetonitrile (Th. Geyer, Renningen); Diaion HP20 resin (>250 µm beads; Thermo Scientific); DMSO-d_6_ (Sigma Aldrich, USA); rifampicin (TCI, Germany); media ingredients (Supplementary Table 1; Carl Roth, Germany). The HRMS data were collected on the Orbitrap Exploris 120 Mass Spectrometer (Thermo Fisher Scientific) equipped with an electrospray ion (ESI) source and a Vanquish Core UHPLC system (Thermo Fisher Scientific). High-performance liquid chromatography (HPLC) was carried out by using a Vanquish Core UHPLC system (Thermo Fisher Scientific). The chromatography conditions are indicated in the respective sections below. The NMR spectra of the peptidic compounds were collected using NMR spectrometers outfitted with Bruker CryoPlatforms (500 and 700 MHz).

### Genome mining

#### Biosynthetic repertoire

To identify strains potentially capable of producing demiguisins with higher yields, partial sequences (*dmgP7*, *dmgP1*, and *dmgP1* to *dmgP7*) of the BGC responsible for producing demiguisin (*dmg*) [location: 317,893–327,300; total length: 9408 nt, region 3.2] from the genome of *Amycolatopsis* sp. PS_44_ISF1 was subjected to nBLAST analysis on NCBI^5^. Genomes of strains showing ≥85% sequence identity with the partial NRPS BGC were retrieved and analysed with antiSMASH v7.1 using bgcFlow wrapper 0.4.0 (conda environment) using default parameters^10,29,30^. In addition to antiSMASH, the genomes of both strains were additionally submitted to PRISM 4, and the substrate specificity of the adenylation domains in *dmg* and *dem* BGCs was predicted using PARAS and PRISM 4^22,23^. BGC similarity networks were constructed with the antiSMASH output results for all strains using BiG-SCAPE v2.0 with the parameters --include_singletons --mix --mibig --version 4.0^11^. The default cutoff value of 0.3 was applied. Networks were visualized in Cytoscape v3.8.1 (https://cytoscape.org/) and manually curated^18^. The *dmg* BGCs were further examined using Clinker of CompArative GEne Cluster Analysis Toolbox (CAGECAT) using the default parameters (min. alignment sequence identity of 0.3), and homologous gene clusters were visualized with Clustermap.js^14^.

#### Mining for *kerA* and *kerC* genes

To assess if both *Amycolatopsis* strains encode for keratinases, the KerA protein sequence (GenBank ID: AAB34259.1) and KerC protein sequence (GenBank ID: ABY65903.1) were used in BLASTp searches against the PS_44_ISF1 and NBC_01488 genomes^20^. The top hits from these searches were tabulated and compared for sequence identity, high query coverage, and *E*-values.

#### Taxonomic classification

To refine taxonomic placement of the strains containing the *dmg* BGC, genome FASTA files were subsequently uploaded to the Type Strain Genome Server (TYGS) for whole-genome-based phylogenomic analysis^7^. The resulting phylogenomic tree was exported in Newick format and visualized using iTOL (Interactive Tree of Life) v7.2.1 (https://itol.embl.de/)^31^. Finally, FastANI was performed using bgcFlow wrapper 0.4.0 to investigate species-level relatedness by calculating pairwise average nucleotide identity (ANI) between the genomes of strains incorporated into the TYGS analysis as the closest related reference strains to PS_44_ISF1 and NBC_01488^9,29^.

### Strain cultivation

#### Media screening

Three media were selected to optimize production of various secondary metabolites (Supplementary Table 1), including PDA, peptone dextrose broth (PDB), and a minimal broth (M9) supplemented with glucose and casamino acids. Seed cultures of *Amycolatopsis* sp. PS_44_ISF1 and *Amycolatopsis* sp. NBC_01488 were prepared in 25 mL of PDB in a 100 mL Erlenmeyer flask and cultivated for three days at 30 °C with shaking at 150 rpm. For PDB and M9 broth cultures, 100 µL of seed culture was used to inoculate 100 mL of PDB or M9 broth (1 mL of seed culture was centrifuged and resuspended in M9 broth prior to inoculation) in 300 mL Erlenmeyer flasks, in triplicate. The flasks were incubated at 30 °C for 14 days with shaking at 180 rpm.

For broth cultures, the culture was transferred to 50 mL falcon tubes, centrifuged at 10,000 rpm for 10 min, and the cell free supernatant was transferred into a Schott bottle containing 20 g/L of MeOH-activated Diaion HP20 resin and left overnight at room temperature. The beads were filtered, rinsed with 10% MeOH, and eluted with 50% and 100% MeOH until the eluent was colourless. The eluent was poured off into round bottom flasks and dried *in vacuo*. The dried extracts from PDB and M9 were then dissolved in 10% MeOH and partially purified using C_18_ solid phase extraction (SPE). The SPE column was first pre-activated (100% MeOH) and equilibrated (10% MeOH) column, where after the crude extracts were loaded onto the column. The column was washed with 10% MeOH, and the metabolites were eluted with 50 and 100% MeOH. Samples were pooled and dried *in vacuo*, and weighed for LC-HRMS/MS analysis. In all cases, media controls were included.

For agar cultures, 100 µl of seed culture was used to spread plate onto 90 mm PDA plates in triplicate, and were incubated at 30 °C for 14 days. Following incubation, the agar was cut into small pieces (1 × 1 cm), and the metabolites were extracted in a beaker with MeOH overnight. Afterwards, the solvent was filtered off (using filter paper) and evaporated *in vacuo*. The dried PDA extracts were then dissolved in 10% MeOH and partially purified using C_18_ solid phase extraction (SPE) as described above.

#### Eco-mimetic cultivations

Two media, containing either pheasant feathers or feather meal (Supplementary Table 1), were selected to investigate whether *Amycolatopsis* sp. PS_44_ISF1 and *Amycolatopsis* sp. NBC_01488 were able to grow and produce demiguisins. Following seed culture preparation, cultures were spun down (8000 rpm, 5 min, 4 °C) and the supernatant was removed. The cell pellet was rinsed with 0.9% NaCl and resuspending in the respective media (OD_600_ = 0.7). Broth cultures were also prepared in 300 mL Erlenmeyer flasks containing 75 mL of feather broth (FB) and feather meal broth (FMB), and inoculated with 5 mL of seed culture. For agar cultures, 5 µL (OD_600_ = 0.1) was used to spot plate onto the feather agar (FA) and feather meal agar (FMA) plates, in triplicate. Agar cultures were incubated at 30 °C for 10 and 14 days, while broth cultures were incubated for 7, 10, and 14 days at 30 °C on an orbital shaker at 180 rpm. Solvent extractions were performed at the respective time points, and all extracts were subjected to SPE, as described in the ‘Production of demiguisins on standard cultivation media’ section. Samples were dried *in vacuo* and weighed for LC-HRMS analysis.

#### Time-dependent production of demiguisins

Seed cultures of *Amycolatopsis* sp. PS_44_ISF1 and *Amycolatopsis* sp. NBC_01488 were prepared in 50 mL PDB for 3 days at 30 °C at 150 rpm. Following incubation, 100 µL of each seed culture was used to inoculate 90 mm PDA plates in triplicate and cultivated for 4, 7, 10, and 14 days at 30 °C. Solvent extractions were performed at the respective time points, and all extracts were subjected to SPE, as described in the ‘Production of demiguisins on standard cultivation media’ section. Samples were dried *in vacuo* and weighed for LC-HRMS analysis.

#### Co-cultivation studies

Seed cultures of *Amycolatopsis* sp. PS_44_ISF1, *Amycolatopsis* sp. NBC_01488, *Kocuria* sp. LS23, and *Kocuria* sp. LS19 were prepared in 25 mL PDB for 3 days at 30 °C at 150 rpm. The PS_44_ISF1 or NBC_01488 seed cultures were used to spread plate 100 µl onto one half of a 120 mm PDA plate. Thereafter, 100 µl of *Kocuria* sp. LS23 or LS19 was spread plated onto the other half of the Petri dish, leaving 1 cm space between the strains. Additional test cultures, including *Streptomyces* sp. M56 and *Rhodococcus* sp. Zoo49 were included in the co-cultivation studies against PS_44_ISF1. A control plate for each strain was prepared on 120 mm PDA plate using 200 µl of each seed culture (monoculture plates). Plates were incubated at 30 °C for 14 days. Thereafter, the *Amycolatopsis* side of the Petri dish, as well as control plates and media control plates, were extracted and purified as described in the ‘Production of demiguisins on standard cultivation media’ section for agar cultures. Samples were dried on the SpeedVac and weighed for LC-HRMS analysis.

#### LC-HRMS/MS analysis

High-resolution ultra-high performance liquid chromatography electrospray ionization mass spectrometry (HR-UHPLC-ESI-MS) measurements were carried out on a Orbitrap Exploris 120 Mass Spectrometer (Thermo Fisher Scientific) equipped with an electrospray ion (ESI) source and a Vanquish Core UHPLC system (Thermo Fisher Scientific) connected to a Phenomenex Kinetex C_18_ column (50 × 2.1 mm, particle size 1.7 μm, pore diameter 100 Å;) at a flowrate of 0.3 mL/min. An injection volume of 5 μL was used with the following gradient elution of solvents A (water, 0.1% [vol/vol] formic acid) and B (acetonitrile, 0.1% [vol/vol] formic acid): 5% B for 1 min; a linear gradient to 100% B for 10 min, then 100% B for 4 min, a gradient to 5% B for 1 min, and finally 5% B for 2 min. The column oven was set to 40 °C, scan range of MS was set to *m/z* 200 to 2000 with a scan speed of 10,000 u/s and event time of 0.25 s under positive mode. Desolvation temperature was set to 250 °C with an interface temperature of 350 °C and a heat block of 400 °C. The nebulizing gas flow was set to 1.5 L/min and dry gas flow to 15 L/min. A combination of data-dependent MS/MS analysis and Top10 experiments was applied, and measurements were performed in centroid mode with a resolving power of 17,500 at *m*/*z* 200, an isolation window of 1.1 *m*/*z* and stepped normalized collision energy of 20/30/40%. HRMS data were analysed using the FreeStyle v1.8 (Thermo Fisher Scientific). Relative abundance was determined by determining the peak area using the extracted ion mode (EIC).

#### GNPS analysis

Tandem mass spectrometry molecular networks were created using the GNPS platform (http://gnps.ucsd.edu). The data files (.RAW) were converted to the .mzML format with MS-Convert v3.0.23194^31^. The converted files were then uploaded to GNPS using WinSCP v6.1.1 and then used to generate an MS/MS molecular network using the GNPS Data Analysis workflow version release 30^15^. The precursor and fragment ion mass tolerance were set to 0.02 Da, and to a product ion tolerance of 0.02 Da. Networks were generated using six minimum matched ion fragments, a minimum cluster size of two, and a cosine score of 0.7. The remaining parameters were kept at default. The library spectra were filtered in the same manner as the input data. All matches kept between network spectra and library spectra were required to have a score above 0.7 and at least six matched peaks. After analysis, all networks were visualized using Cytoscape v3.10.0 software^18^.

### Large scale fermentation and isolation of demiguisin derivatives

A seed culture of *Amycolatopsis* sp. NBC_01488 was prepared in 100 mL of PDB for 3 days at 30 °C at 150 rpm. The NBC_01488 seed cultures were used to spread plate 200 µl onto one side of a 120 mm PDA plate (4 L of PDA in total). Plates were cut into small cubes (1 × 1 cm) and extracted with MeOH overnight. Afterwards, the solvent was filtered off and evaporated *in vacuo*. The dried extract was then dissolved in 10% MeOH and subjected to SPE. Fractions were collected when eluting with 40, 80, and 100% MeOH (2 CV). The fractions were concentrated under reduced pressure, subjected to MS, and later antibacterial activity assays. Demiguisin derivatives eluted in the 40% MeOH fraction, and fraction (resuspended in 50% MeOH) was separated by semi-preparative reverse phase HPLC using a Vanquish Core UHPLC system equipped with a Phenomenex Luna Phenyl-hexyl 250 × 10 mm column (particle size 5 µm, pore diameter 100 Ǻ column). A gradient solvent system of: 0.1% formic acid (FA) in analytical grade water (A) and 0.1% FA in acetonitrile (B) with the following gradient: 13% B (0–5 min), 13%–35% B (5–35 min), 35%–100% B (35–38 min), 100% B (38–40 min) and 100%-13% B (42–45 min) was used with a flow rate of 2 mL/min, UV detection at 214 nm and column oven was set to 25 °C. This resulted in pure demiguisin derivatives eluted between 20 and 31 min.

#### NMR

Spectra of the peptidic compounds were collected using NMR spectrometers outfitted with Bruker CryoPlatforms (DMSO-d_6_, 500, 700 MHz). MestReNova ver. 12.0.1 was used to process the data for the NMR spectra.

### Physical data

**Demiguisin A (1)**: white solid, UV (acetonitrile), *λ*_max_ 214 nm; NMR spectral data (DMSO-d_6_), see Supplementary Table 7 and Supplementary Figs. 7–12; HR-ESI-MS (Supplementary Table 9) *m*/*z* 694.3510 [M + H]^+^ calcd. for C_30_H_47_N_9_O_10_ at 694.3524 ∆_ppm_ 2.02.

**Demiguisin B (2)**: white solid; UV (acetonitrile), *λ*_max_ 214 nm; NMR spectral data (DMSO-d_6_), see Supplementary Table 7 and Supplementary Figs. 13–17; HR-ESI-MS (Supplementary Table 9) *m*/*z* 678.3571 [M + H]^+^ calcd. for C_30_H_47_N_9_O_9_ at 678.3575, ∆_ppm_ 1.77.

**Demiguisin C (3)**: white solid powder; UV (acetonitrile), *λ*_max_ 214 nm; NMR spectral data (DMSO-d_6_), see Supplementary Table 8 and Supplementary Figs. 18–21; HR-ESI-MS (Supplementary Table 9) *m*/*z* 637.3299 [M + H]^+^ calcd. for C_28_H_44_N_8_O_9_ at 637.3309, ∆_ppm_ 1.57.

**Demiguisin D (4)**: white solid; (acetonitrile), *λ*_max_ 214 nm; NMR spectral data (DMSO-d_6_), see Supplementary Table 8 and Supplementary Figs. 22–25; HR-ESI-MS (Supplementary Table 9) *m*/*z* 621.3348 [M + H]^+^ calcd. for C_28_H_45_N_8_O_8_ at 621.3360, ∆_ppm_ 1.93.

**Demiguisin E (5)**: white solid; UV (acetonitrile), *λ*_max_ 214 nm; NMR spectral data (DMSO-d_6_), see Supplementary Table 8 and Supplementary Figs. 26–28; HR-ESI-MS (Supplementary Table 9) *m*/*z* 621.3378 [M + H]^+^ calcd. for C_28_H_45_N_8_O_8_ at 621.3360, ∆_ppm_ 2.90.

**Demiguisin F (6)**: white solid; UV (acetonitrile), *λ*_max_ 214 nm; NMR spectral data (DMSO-d_6_), see Supplementary Table 8 and Supplementary Figs. 29–31; HR-ESI-MS (Supplementary Table 9) *m/z* 605.3400 [M + H]^+^ calcd. for C_28_H_45_N_8_O_7_ at 605.3411, ∆_ppm_ 1.82.

### Biological activity of demiguisin derivatives

#### Keratin azure assay

Seed cultures of PS_44_ISF1 and NBC_01488 were grown in 100 mL of PBD for 7 days. After incubation, the cell pellets were washed in 0.9% (w/v) NaCl solution and resuspended in the respective feather medium to an OD_600_ = 0.7. Thereafter, 5 mL of standardized culture volumes were mixed with either 100 mL of FB or FMB (Supplementary Table 1). Three technical replicates were prepared and placed on a thermoshaker at 180 rpm for 7 days at 30 °C, as well as FB and FMB only for the controls.

Following incubation, the keratinolytic activity was determined by preparing keratin azure (Sigma-Aldrich; cut into small pieces, washed twice with deionized water, and dried). Then, 10 mg of keratin azure was mixed with 800 µl of Tris-HCl (pH 8), followed by the addition of 500 µL of culture supernatant containing extracellular enzymes. The reaction mixtures were incubated for 3 h at 30 °C with shaking at 300 rpm. The reaction was terminated by centrifugation at 10,000 rpm for 10 min at 4 °C. Subsequently, 200 µL of the supernatant from each replicate was transferred into a 96-well plate, and the absorbance was measured at 595 nm. The control FB and FMB were used without additives as the negative control, and 1 µL of protease K was added for the positive control. A calibration curve was created for each medium individually using the following values: 0% for negative control, 100% for positive control. Keratinolytic activity of the samples was subsequently expressed relative to these reference values, allowing comparison of keratin degradation between media and strains^32,33^.

#### Activity of fractions against feather degrading bacteria

SPE fractions (10 mg/mL in 50% MeOH, diluted to 1 mg/mL in water) were tested for their activity against *Kocuria* sp. LS19, *Curtobacterium* sp. LS33, *Curtobacterium* sp. LS34, and *Kocuria* sp. LS38 using a disc diffusion assay^34^. All test microorganisms were inoculated into Mueller-Hinton broth (MHB) and were incubated at 37 °C for 18–24 h. Following incubation, 100 μL of the bacterial suspension (OD_600_ = 0.08–0.13) was spread-plated onto Mueller-Hinton agar (MHA, Merck) to create a microbial lawn. On each plate, 6 mm blank discs (Oxoid) were added using a sterile needle, and 50 µL compound solution (50 µg) was applied. A negative control of 10% MeOH was included for each strain, while 50 µL of rifampicin (1 mg/mL) was included as a positive control. Antimicrobial activity was determined by measuring the inhibition zone surrounding the disc in mm.

#### Bacterial strains and growth conditions for activity of pure compounds

All bacterial isolates were handled following standard microbiological procedures. *M. tuberculosis* ATCC 25177, *M. avium* ATCC 700898, *S. aureus* ATCC 29213 and *E. coli* ATCC 25922 were purchased from the American Type Culture Collection (ATCC), USA. *M. intracellulare* ATCC 13950 and *M. abscessus* ATCC 19977 were acquired from the German Collection of Microorganisms and Cell Cultures (DSMZ). *E. coli* WO153 strain was generously provided by the Antimicrobial Discovery Center, Department of Biology, Northeastern University. Additional strains were obtained from our internal collection and cultured under conditions recommended by their respective depositors.

#### Antimicrobial susceptibility testing

The minimum inhibitory concentrations (MICs) of demiguisins were determined using the standard broth microdilution method in Mueller-Hinton Broth II (cation-adjusted; Sigma) according to EUCAST guidelines. Briefly, 75 µL of bacterial suspension (~4 × 10⁵ colony-forming units (CFU)/mL) in Mueller-Hinton Broth II was added to each well of a 96-well plate, along with 75 µL of serially diluted compound solutions (starting concentration 64 µg/mL). Plates were incubated at 37 °C for 18–24 h, and the MIC was reported as the lowest concentration that inhibited visible bacterial growth.

For mycobacterial strains, MIC determination was performed in 7H9 complete medium (BD Difco; Becton Dickinson) supplemented with 10% oleic acid-albumin-dextrose-catalase (OADC; BD), as described previously^35^. A single-cell suspension of mycobacterial cultures was prepared in PBS and adjusted to a final concentration of 10⁵ CFU/mL for *M. tuberculosis* and *M. marinum* and 5 × 10^4^ for *M. avium*, *M. intracellulare*, and *M. absecuss*. Compounds were tested in two-fold serial dilutions with a final DMSO concentration of 1%. Plates were incubated at 37 °C under conditions appropriate for the growth of the respective mycobacterial species, and the MIC was determined as the lowest concentration showing no visible growth.

#### Cytotoxicity studies using HepG2 cells

HepG2 cells (human hepatoblastoma cell line; DSMZ cell culture no.: ACC 180) were cultured according to the supplier’s instructions in Dulbecco’s modified Eagle medium (DMEM) supplemented with 10% fetal bovine serum. Cytotoxicity of the compounds was assessed as described previously^36^. Briefly, cells were seeded at 6 × 10³ cells per well in 96-well plates with 120 µL of complete medium. After a 2 h equilibration period, serially diluted compounds were added in 60 µL of complete medium. Compounds, solvent control, and doxorubicin (used as a reference compound) were tested in duplicate in two independent experiments. Following 5 days of incubation, 20 µL of MTT (5 mg/mL thiazolyl blue tetrazolium bromide in PBS) was added to each well, and the plates were incubated for an additional 2 h at 37 °C. The medium was then removed, and the cells were washed with 100 µL PBS. Formazan crystals were dissolved by adding 100 µL of 2-propanol/10 N HCl (250:1) was added. Absorbance was measured at 570 nm using a microplate reader (Tecan Infinite M200Pro). Cell viability was calculated relative to the solvent control, and IC_50_ values were determined by sigmoidal curve fitting using GraphPad PRISM 8 (GraphPad Software).

### BLASTp and multiple protein sequence alignment

To gain better insight into the biosynthetic pathway of a non-proteinogenic amino acid (Piz-1) in the demiguisin derivatives, protein sequences corresponding to DemCYP450 (Accession no. CP109434.1), DmgCYP450 (Accession no. JANUXO000000000.1), PlyM_CYP450 (Accession no. KF386858.1), and HmtN_CYP450 (Accession no. FR823394.1) were retrieved from antiSMASH genome mining results. Pairwise sequence identity between DemCYP450 and the other CYP450 homologues was initially assessed using BLASTp (NCBI) analysis. To further evaluate sequence conservation, the amino acid sequences were aligned using Clustal Omega via the EMBL-EBI web server (https://www.ebi.ac.uk/jdispatcher/msa/clustalo) with default parameters^37^. The resulting multiple sequence alignment (MSA) was generated in Clustal format and used to assess sequence identity, similarity, and conserved motifs across the CYP450 proteins. The MSA output was subsequently visualized using MView, which produced a formatted alignment figure highlighting conserved residues and regions of divergence. Sequence identity and similarity values were extracted from the Clustal Omega output to support comparative analysis of CYP450 homologues.

## Supplementary information

Transparent Peer Review file

Supplementary_Information

Description of additional data

Supplementary Data 1

Supplementary Data 2

Supplementary Data 3

Supplementary Data 4

Supplementary Data 5

## Data Availability

All media, genome, and spectral data generated during this study (^1^H NMR, ^13^C NMR, COSY, HSQC, HMBC, HRMS/MS) are provided in the Supplementary Information file (PDF). The raw MS data has been made available is in the open database Zenodo (https://doi.org/10.5281/zenodo.19414529). The source data for Supplementary Fig. 38 is Supplementary Data 1. The source data for Fig. 3 is Supplementary Data 2. The source data for Supplementary Fig. 4 is in Supplementary Data 3 and 4. The source data for Supplementary Fig. 5 is in Supplementary Data 5.
