# Supplementary material for: Targeted pathway mining reveals novel modified hexapeptides from the demiguisin family
Source: Commun Chem. 2026 Oct 17;9:327. doi: 10.1038/s42004-026-02250-w (PMC13676617; doi:10.1038/s42004-026-02250-w)
Supplement: Supplementary file 1 [file 42004_2026_2250_MOESM1_ESM.pdf]

nature portfolio

Peer Review File

Targeted Pathway Mining Reveals Novel Modified Hexapeptides from the Demiguisin Family

Corresponding Author: Professor Christine Beemelmanns

This file contains all reviewer reports in order by version, followed by all author rebuttals in order by version.

Attachments originally included by the reviewers as part of their assessment can be found at the end of this file.

Version 0:

Reviewer comments:

Reviewer #1

(Remarks to the Author)
This study employs targeted genome mining to identify a soil-derived Amycolatopsis strain capable of producing demiguisin-class hexapeptides. Comparative metabolomics and optimized cultivation enabled the isolation and structural characterization of five new demiguisin derivatives, which vary in piperazic acid hydroxylation patterns and the presence of a C-terminal glycine residue. Biological evaluation revealed selective antimycobacterial activity, against Mycobacterium intracellulare and moderate activity against M. tuberculosis. The paper is well written and the experiments are straightforward and conclusive.

I have some concerns:
1. The lack of any genetic evidence for the identity of the gene cluster. There are genetic tools available for Amycolatopsis to delete the central biosynthetic gene and confirm the biosynthetic origin of the compounds.
2. Inactivation of the TE domain coupled with an ejection assay could give further information about whether the lack of the terminal glycine is caused by TE activity or if the last module is “skipped” regularly.
3. The authors emphasize the ecological importance of the compounds due to the activity against Mycobacteria. However, there are other bacterial species e.g. Chlamydia psittaci, Campylobacter, Salmonella and others that are often found to be associated with birds. Are the compounds specifically antimycobacterial or protect against other avian-associated bacteria? Also, the activity against M. avium is relatively low and I think the ecological framing is overstated given the narrow pathogen panel.

Reviewer #2

(Remarks to the Author)
I co-reviewed this manuscript with one of the reviewers who provided the listed reports. This is part of the Communications Chemistry initiative to facilitate training in peer review and to provide appropriate recognition for Early Career Researchers who co-review manuscripts.

Reviewer #3

(Remarks to the Author)
This manuscript is certainly interesting and includes a substantial amount of experimental work. The combination of genome mining, comparative metabolomics, and pathway analysis is potentially valuable, and the identification of new demiguisin analogues contributes to the expanding family of piperazic acid-containing peptides. However, the manuscript often appears unfocused and somewhat chaotic in its organization. Several secondary aspects are discussed in considerable detail despite being only marginally connected to the main topic, whereas important structural and biosynthetic questions are not sufficiently addressed.
Overall, the manuscript contains interesting findings and a large amount of experimental work. However, in its current form, it suffers from problems of focus and from insufficiently rigorous structural and biosynthetic characterization, particularly for the

minor congeners. I believe that substantial revision and a more coherent presentation are necessary before the manuscript can be considered for publication.

Major issues
i. Overall focus and organization - The manuscript would benefit from a clearer focus. In several sections, side topics are discussed extensively without a direct connection to the central theme of the work. In particular, the discussion on the rifamycin-like PKS cluster appears largely outside the main scope of the manuscript, which should primarily concern the demiguisins and their biosynthesis.

ii. Feather-based media and keratin degradation - I do not clearly understand the connection between:
• feather-based media,
• keratin hydrolysis,
• keratinases,
• and demiguisin biosynthesis.
iii. The manuscript includes an extended discussion on kerA/kerC-like genes and keratin degradation, but the relevance of these observations to secondary metabolite biosynthesis remains speculative and experimentally unsupported. In addition, the concept of “feather-based media” should be explained more clearly for the reader. At present, it is not obvious whether these conditions are biologically meaningful for metabolite production or simply intended to mimic the ecological niche of the bird-associated strain.

iv. Structural characterization of the minor compounds - Except perhaps for compound 2, the structural characterization of the minor congeners appears rather limited and, in some cases, not sufficiently rigorous. In particular:
• a clear carbon numbering scheme for the demiguisins is missing;
• key NMR correlations supporting the peptide backbone assembly are not adequately presented;
• several assignments appear to rely mainly on HRMS/MS data and biosynthetic analogy.
More complete HMBC/COSY/ROESY information and a clearer discussion of the structural assignments would significantly strengthen this section.

v. Relative and absolute stereochemistry - The stereochemical discussion is rather weak for peptide natural products of this type. The manuscript essentially assumes identical stereochemistry for all derivatives based on BGC homology and the absence of epimerase domains, but this alone is not sufficient to support the absolute stereochemical assignments. Given the peptidic nature of these compounds, one would expect:
• complete and clearly discussed Marfey analyses,
• stronger ROESY/J-based analysis,
• or more convincing biosynthetic evidence.
In addition, the relative stereochemistry of the hydroxylated Piz residues is barely discussed.

vi. Hydroxylated Piz biosynthesis - Although the section title suggests mechanistic insight, the manuscript does not clearly explain the origin of the different hydroxylation patterns observed in the Piz residues. The proposed involvement of DemAA3/DmgAA3 and/or the CYP450 enzyme remains highly speculative and is not supported by genetic or biochemical evidence. A clearer biosynthetic scheme for hydroxylated Piz formation would be very useful, together with a more detailed discussion of β-OH versus γ-OH Piz formation, sequence of hydroxylation, and comparison with known piperazic acid biosynthetic pathways.

vii. Biological activity - The antimycobacterial activity is interesting, particularly against Mycobacterium intracellulare , but the biological evaluation remains relatively limited. A more detailed discussion of possible structure–activity relationships, especially concerning the role of the terminal Gly residue, would improve the biological part of the manuscript.

Version 1:

Reviewer comments:

Reviewer #1

(Remarks to the Author)
My comments have been addressed.

Reviewer #2

(Remarks to the Author)
I co-reviewed this manuscript with one of the reviewers who provided the listed reports. This is part of the Communications Chemistry initiative to facilitate training in peer review and to provide appropriate recognition for Early Career Researchers who co-review manuscripts.

Reviewer #3

(Remarks to the Author)
Recommendation: Major Revision

The revised manuscript is substantially improved compared with the original submission. The authors have carefully addressed most of my previous comments, and the manuscript is now considerably more focused, better organized, and easier to follow. I particularly appreciate the removal of sections that were not directly relevant to the main objective and the improved discussion of the structural characterization and biosynthetic hypotheses. Overall, the work now represents a much stronger contribution.
Nevertheless, I believe that a few important issues still deserve attention before the manuscript can be considered for publication.
Major comments
1. Further improvement of the manuscript presentation
Although the manuscript has been significantly improved, the document would still benefit from careful text editing. There are several typographical errors that should be corrected (for example, on page 27, line 571, "Fig. SXX" should probably refer to "Fig. S40"). In addition, the narrative occasionally remains somewhat fragmented, giving the impression that sections have been inserted or modified independently rather than fully integrated into a coherent story. A final editorial revision would greatly improve readability.
2. Relationship between keratin degradation and demiguisin biosynthesis
I still remain unconvinced that the keratinase description is sufficiently justified within the context of this manuscript. Although the authors have clarified that this part aims to provide ecological context rather than directly explain demiguisin biosynthesis, the experimental data presented do not convincingly support a relationship between keratin utilization and production of the demiguisins.
The growth experiments on feather-containing media are interesting, but metabolite production is mainly discussed for PDB and PDA cultures. If the authors wish to maintain the ecological argument, they should also report secondary metabolite production, particularly demiguisins, under the feather-based media (FA and FMA). It would be very informative to perform the same GNPS/SIRIUS analysis under these conditions and determine whether keratin-rich substrates actually influence metabolite production or congener distribution.
Moreover, while the manuscript compares the ability of the two strains to grow on feather-derived substrates, no direct measurement of keratin degradation or keratinase activity is presented. Therefore, the conclusion that one strain possesses a superior keratinolytic capacity remains largely indirect.
3. Use of the term "metabolomics"
From a methodological point of view, I am not convinced that the work actually performs a metabolomic analysis in the strict sense. Rather, the study consists of MS-based molecular annotation using GNPS, SIRIUS and database-assisted dereplication. I would encourage the authors to adopt more precise terminology throughout the manuscript and avoid referring to this approach simply as "metabolomics."
4. Structural characterization
The structural characterization has been substantially improved and is now considerably more convincing. Likewise, the discussion concerning the origin of the different demiguisin congeners remains partly speculative, but the authors clearly acknowledge these limitations. I also appreciate that the stereochemical assignments are now explicitly presented as being based on comparison with the known compound together with biosynthetic considerations, which is a more balanced and scientifically rigorous presentation.
5. Biological activity and ecological interpretation
The biological section has also improved, but I still find the discussion only partially convincing.
Overall, the demiguisins display relatively modest antimicrobial activity, with rather high MIC values against most tested microorganisms. The only particularly interesting result is the activity against Mycobacterium intracellulare. However, the manuscript does not report the activity of the other major secondary metabolites produced by Amycolatopsis sp. PS_44_ISF1 and NBC_01488 (for example, the rifamycin congeners or the pachycephalamides) against this microorganism. Without these comparisons, it is difficult to evaluate whether demiguisins truly represent the biologically relevant antimicrobial metabolites produced by these strains.
Similarly, although the authors discuss the possible ecological relevance of the compounds in the context of avian-associated mycobacteria, I could not find data on the activity of the demiguisins against Mycobacterium avium subsp. avium, which is cited in the manuscript as the major causative agent of avian tuberculosis. These data would considerably strengthen the ecological interpretation. In their absence, the discussion should be moderated further.
Overall, I believe that additional experimental evidence or a more cautious interpretation is necessary before meaningful conclusions regarding the antimicrobial and ecological significance of these compounds can be drawn.

Version 3:

Reviewer comments:

Reviewer #3

(Remarks to the Author)
The authors have satisfactorily addressed most of my previous concerns, particularly regarding structural characterization, the biosynthetic hypotheses, and the experimental support for keratin degradation. I therefore consider the manuscript suitable for publication.
A few minor issues remain, mainly about the strength and consistency of some statements across different sections. In particular, in my opinion, the proposed role of demiguisins in avian host defence remains speculative while the relationship between the glycine residue and antimycobacterial activity should not be presented as causal. These points do not require additional experiments, but would benefit from minor rewording to ensure that the conclusions remain fully consistent with the limitations appropriately acknowledged elsewhere in the revised manuscript. Specifically:

1) the Abstract should further moderate the proposed role of demiguisins in avian host defence;
2) the Conclusion should avoid implying a causal dependence of antimycobacterial activity on the glycine residue.

In addition, the Results and Discussion/Methods should use the term “metabolomics” more precisely where the analysis is essentially targeted or comparative metabolite profiling.

Open Access This Peer Review File is licensed under a Creative Commons Attribution 4.0 International License, which permits use, sharing, adaptation, distribution and reproduction in any medium or format, as long as you give appropriate credit to the original author(s) and the source, provide a link to the Creative Commons license, and indicate if changes were made.
In cases where reviewers are anonymous, credit should be given to 'Anonymous Referee' and the source.
The images or other third party material in this Peer Review File are included in the article’s Creative Commons license, unless indicated otherwise in a credit line to the material. If material is not included in the article’s Creative Commons license and your intended use is not permitted by statutory regulation or exceeds the permitted use, you will need to obtain permission directly from the copyright holder.
To view a copy of this license, visit https://creativecommons.org/licenses/by/4.0/

We very much appreciate the reviewers time to carefully evaluate the manuscript and for their constructive feedback that helped to improve the manuscript. Please find comments addressing revision recommendations for the manuscript COMMSCHEM-26-0294 in a point-to-point response.

Reviewer 1 remarks:

This study employs targeted genome mining to identify a soil-derived *Amycolatopsis* strain capable of producing demiguisin-class hexapeptides. Comparative metabolomics and optimized cultivation enabled the isolation and structural characterization of five new demiguisin derivatives, which vary in piperazic acid hydroxylation patterns and the presence of a C-terminal glycine residue. Biological evaluation revealed selective antimycobacterial activity, against *Mycobacterium intracellulare* and moderate activity against *M. tuberculosis*. The paper is well written and the experiments are straightforward and conclusive.

1. The lack of any genetic evidence for the identity of the gene cluster. There are genetic tools available for *Amycolatopsis* to delete the central biosynthetic gene and confirm the biosynthetic origin of the compounds.

2. Inactivation of the TE domain coupled with an ejection assay could give further information about whether the lack of the terminal glycine is caused by TE activity or if the last module is "skipped" regularly.

Response: We are thankful for the critical evaluation and would highlight that genetic validation of the biosynthetic gene cluster was not repeated in the present study, as its identity had already been experimentally confirmed in a previous publication from our group (DOI: 10.1038/s41467-024-52316-3), including knockout studies in the wild-type strain that demonstrated its role in compound biosynthesis.

We agree that targeted experiments, such as TE domain inactivation, would provide valuable insight into pathway function. However, such studies require transfer of the complete or partial BGC into a heterologous host. Due to the large size and structural complexity of the pathway, successful cloning and heterologous expression have not yet been achieved and remain ongoing work. We therefore consider these experiments beyond the scope of the present study. Nevertheless, we have acknowledged this important direction for future investigation in the revised discussion. We have also clarified this point in the manuscript and added the appropriate reference to guide readers to the prior genetic validation study.

3. The authors emphasize the ecological importance of the compounds due to the activity against *Mycobacteria*. However, there are other bacterial species e.g. *Chlamydia psittaci*, *Campylobacter*, *Salmonella* and others that are often found to be associated with birds. Are the compounds specifically antimycobacterial or protect against other avian-associated bacteria? Also, the activity against *M. avium* is relatively low and I think the ecological framing is overstated given the narrow pathogen panel.

Response: Thank you for this insightful comment. While we agree that testing additional avian-associated bacterial species would further broaden the ecological context of the study, the limited amounts obtained from purification unfortunately prevented the expansion of the broader

antimicrobial screening to additional strains and thus we decided to focus on testing members of one genera. We also agree that the ecological relevance of the antimycobacterial activity should be interpreted cautiously. Nevertheless, the activity observed against *Mycobacterium intracellulare* (MIC = 2 µg/mL) remains notable and, in our view, justifies discussion in the manuscript. Nontuberculous mycobacteria have been reported in avian-associated environments and may act as opportunistic pathogens in birds. While we do not propose that demiguisins evolved specifically as antimycobacterial agents, the selective activity against mycobacteria suggests a potentially relevant ecological role that warrants further investigation. To avoid overstating this point, we revised the manuscript to moderate the ecological interpretation and more clearly acknowledge the limitations of the current pathogen panel.

Reviewer 2 remarks:

The manuscript by Decker et al. investigates the dem biosynthetic gene cluster (BGC) from a soil-derived *Amycolatopsis* strain. …. The authors identified six demiguisin congeners in total, including five previously undescribed derivatives, and linked their production to medium-dependent metabolic profiles. Interestingly, the study is methodologically strong integrating in-silico and wet-lab approaches into a single workflow.

The main findings of the manuscript are truly interesting and were obtained using the correct experimental pipeline. However, the current version of the manuscript is not consistent and confusing in some parts. Several sections of the manuscript appear disconnected from the overall context (see below). These sections stray from the authors' original objectives, which are currently not consistent with the presentation of the results.

In my opinion, the authors have not fully described (or achieved) the aim of the manuscript "we hypothesized that the structural diversity within the demiguisin compound family might be broader than originally described". The authors should focus on the true purpose of the manuscript - the comparison between BGC and an explanation of the biosynthetic capabilities of the selected microorganisms. At present, however, the manuscript consists of parts that are either completely or partially unrelated to its true purpose:

Lines 120-159: Conditions included nutrient-rich and nutrient-poor media, such as peptone dextrose broth (PDB) and agar (PDA), M9 minimal medium (M9 broth), as well as habitat specific growth conditions using feather-based media, including cut pheasant feather agar (FA) and feather-meal agar (FMA). Here, it is worth noting that the bird-derived strain PS_44_ISF1 exhibited robust growth on both feather based substrates (FA and FMA), whereas the soil-derived strain NBC_01488 grew only on pre-processed FMA and failed to grow on non-processed pheasant feathers indicating differences within their encoded capacity to degrade biopolymers (Fig. 3A). …

1. **Do the authors intend to investigate the overall metabolomic capabilities of the strains under study or do they want to focus on the BGCs related to demiguisin? At the moment, this section is too disconnected from the rest of the manuscript.**

Response: Thank you for the suggestion. We agree that the original section placed too much emphasis on the overall metabolomic profiles, which made its connection to the main focus of the manuscript less clear. To improve coherence, we have substantially shortened the description of the annotated metabolites and reframed the section to emphasize culture optimization related to demiguisin production. Specifically, detailed discussion of known metabolites was reduced to brief mentions of annotated features, while greater focus was placed on the media, time and demiguisins (Lines 70–73, 145, and 178–189).

1. **Lines 170-181: we mined the genomes for keratinases, commonly assigned as subtilisin-like serine proteases of the S8 family.Among these, KerA (GenBank ID:176 AAB34259.1) from *Bacillus licheniformis* and KerC (GenBank ID: ABY65903.1) from *Bacillus subtilis* are among the best-characterized keratinases and were used as reference sequences in a dedicated BLASTp – Why do the authors move the focus from the biosynthesis of Demiguisin to keratinase family?**

   Response: We thank the reviewer for this important comment. The inclusion of keratinase (S8 family subtilisin-like protease) mining was not intended to shift the focus away from demiguisin biosynthesis, but rather to provide ecological and physiological context for the origin and lifestyle of the producing strains. Specifically, this analysis was performed to assess whether the bird-associated and soil-derived *Amycolatopsis* strains differ in their genomic potential to degrade keratin, as a proxy for their ability to utilise feather-derived substrates for growth. Since feather material represents a likely nutrient source in the avian-associated environment from which PS_44_ISF1 was isolated, this information was included to support discussion of strain adaptation and substrate utilization relevant to metabolite production conditions. However, we acknowledge that this section may have appeared disconnected from the main biosynthetic focus. We have therefore revised this and other manuscript sections to improve clarity and structure.

2. **Lines 290 – 306: Intrigued by the presence of the antibiotic-acting rifamycin congeners, we pursued a clinker alignment analysis using the rifamycin reference rif BGC from A. mediterranei S699 with the rfm BGC identified in PS_44_ISF1 (Table S11, Fig. S38)22 to estimate its structural relatedness to previously reported pathways. Overall, an extensive conservation between the rif and rfm BGCs was observed, including strong alignments across the type I polyketide synthase (PKS) genes (rifA–rifF), starter unit biosynthesis genes (rifG–rifI and rifK–rifN), and post-biosynthetic modification genes (rifS, rifT, and ORF4), which led us to conclude that the diversity of observed rifamycin congeners, while not yet annotated, likely encompass mostly structurally explored rifamycin congeners**
   – Why the authors add this comparison rfm/rif BGC??? The manuscript should be thoroughly revised by the authors, focusing on their original objectives and avoiding the inclusion of information that, while certainly valid, is misleading in the context of the analysis comparing BGCs.

   Response: We thank the reviewer for this constructive comment. We agree that the inclusion of the rifamycin (rfm) BGC comparison was outside the central scope of the manuscript, which is focused on the demiguisin derivatives and their biosynthesis. In accordance with the reviewer's recommendation, we have therefore removed the rifamycin BGC comparison section (lines 290–306), along with the associated figure and supplementary material, from the revised manuscript.

3. **The authors compare the BGCs in first part of the manuscript but at line 300 they compare the architecture of the BGC homolog *dem* (NBC_01488) and *dmg* (PS_44_ISF1) in greater detail. Maybe it would be better to present a single comparison of the BGCs rather than splitting them into one in "less detail" and another in "greater detail." The authors should reorganize the manuscript and revise it accordingly.**

   Response: The two sections were intentionally separated because they serve different purposes within the manuscript. The initial comparison was included as part of the genome mining strategy used to identify a new strain potentially capable of producing these compounds. In contrast, the later, more detailed comparison of the *dem* (NBC_01488) and *dmg* (PS_44_ISF1) BGC architectures was intended to support the biosynthetic discussion of the newly identified derivatives following

compound elucidation. While we still believe the detailed biosynthetic analysis is most appropriately placed in the later section of the manuscript, we have revised the text in both sections to clarify the distinct objective of the section.

4. **The identification and characterization of the six demiguisin derivatives (678.3563, 637.3299, 621.3348, 621.3378, 605.3411 m/z) is very interesting, but the authors have provided few explanations (even speculating) regarding the alternative enzymatic steps for the biosynthesis of these products.**

   Response: Thank you for this comment. The biosynthetic logic underlying these alternative enzymatic steps has previously been investigated and described in detail in an earlier publication from our research group (DOI: 10.1038/s41467-024-52316-3), which established the core biosynthetic framework for this pathway. In the present study, we therefore focused the biosynthetic pathway section on the mechanistic analysis relevant to the formation of the newly isolated derivatives, rather than reiterating biosynthetic details that have already been reported. We have clarified this point in the revised manuscript and included the appropriate citation to direct readers to the earlier work for a more comprehensive description of the pathway as follows:

5. **Authors should improve the descriptions of the methods. Particularly authors should highlight all values of the parameters used for predictive analyses and bioinformatics mining.**

   Response: The methods section was updated to include the requested parameters.

6. **Conclusions are too generic, please improve**
   Response: Many thanks for the comment – we have revised the Conclusion to accommodate the importance of the overall findings and outline what lies ahead.

7. **Lines 51-54 - "*Amycolatopsis* sp. PS_44_ISF1 derived from the UG of the toxic bird *Pachycephala schlegelii*, that bacterial metabolites such as the antifungal macrolactam ciromicin A,3,4 the co-secreted tensioactive lipopeptide family pachycephalamides, and a highly modified hexapeptide, named demiguisin A (1) can directly contribute to the bird's defense against microbial pathogens" – Please rephrase, missing verbs/confusing sentence.**

   Response: Many thanks for indicting unclear statements, the sentence and paragraph has been rephrased.

8. **Line 75 – "using a targeted nBLAST (NCBI)" - Please details what the authors mean with "targeted"?**
9.

   Response: We have rephrased the unclear sentence and placed it into more context.

10. **Line 79 – "Detailed pairwise comparison using clinker (CAGECAT webserver) revealed extensive synteny and high sequence identity (>70%) across the core NRPS loci (Fig. 2A)" – Please add percentage identities in supplementary table.**

Response: A supplementary table with the percent identities of the sequence similarities between the core NRPS genes of the two BGCs was included in the supplementary file (Fig. S2C).

11. **in-house *Amycolatopsis* strains (M39 and DSM 44993) containing experimentally validated BGCs for comparison – please explain this choice (e.g. positive control)?**
Response: These in-house *Amycolatopsis* strains (M39 and DSM 44993) were included as reference strains for BGC dereplication and comparative analysis of the newly identified NBC_01488 strain. Both strains contain experimentally validated biosynthetic gene clusters that have been characterized in previous publications by our research group, making them suitable positive references for assessing BGC similarity and metabolite annotation. This was reiterated in the manuscript text.

12. **Authors should be more consistent along the entire manuscript descripting the similarities between BGCs :Fore examples, in the first part of the manuscript "Although demiguisin BGCs, *dmg* (*Amycolatopsis* sp. PS_44_ISF1) and *dem* clusters (*Amycolatopsis* sp. NBC_01488), harbored similar core genes associated with amino acid biosynthesis and a NRPS assembly line, they did not cluster together in the BiG-SCAPE network under standard cutoffs (Fig. S2), likely due to variability in flanking or auxiliary genes outside the NRPS region (Fig. S1) – then "the minor detectable differences within the encoded demiguisin-related pathways were reflected".**
Based on these evaluations the manuscript certainly represents a valuable contribution to Communication in Chemistry, but the present version appeared confusingly written and preliminary. My suggestion is to invite the authors to submit a new and revised version of the manuscript before proceeding with the peer-review process.

Response: We thank the reviewer for this detailed and thoughtful evaluation of our manuscript. We appreciate the concern regarding consistency in how similarities between the *dem* (*Amycolatopsis* sp. NBC_01488) and *dmg* (*Amycolatopsis* sp. PS_44_ISF1) biosynthetic gene clusters were described throughout the text.

In the revised manuscript, we have carefully restructured and standardized the description of the BGC comparisons to improve clarity and consistency. In particular, we have harmonized the language used to describe shared core NRPS architecture and amino acid biosynthetic genes, while more clearly distinguishing these conserved features from variability observed in flanking or accessory regions. We have also revised statements that previously referred to "minor detectable differences" to ensure they are consistently framed in the context of NRPS core conservation versus peripheral genomic variation.

Additionally, we have clarified the interpretation of the BiG-SCAPE analysis by explicitly stating that the lack of clustering under standard thresholds reflects variability in non-core regions rather than divergence of the central biosynthetic logic. These revisions are intended to improve readability, reduce ambiguity, and strengthen the overall coherence of the comparative genomic analysis.

Reviewer 3 remarks:

I co-reviewed this manuscript with one of the reviewers who provided the listed reports. This is part of the Communications Chemistry initiative to facilitate training in peer review and to provide appropriate recognition for Early Career Researchers who co-review manuscripts.

Reviewer 4 remarks:

This manuscript is certainly interesting and includes a substantial amount of experimental work. The combination of genome mining, comparative metabolomics, and pathway analysis is potentially valuable, and the identification of new demiguisin analogues contributes to the expanding family of piperazic acid-containing peptides. However, the manuscript often appears unfocused and somewhat chaotic in its organization. Several secondary aspects are discussed in considerable detail despite being only marginally connected to the main topic, whereas important structural and biosynthetic questions are not sufficiently addressed.

Overall, the manuscript contains interesting findings and a large amount of experimental work. However, in its current form, it suffers from problems of focus and from insufficiently rigorous structural and biosynthetic characterization, particularly for the minor congeners. I believe that substantial revision and a more coherent presentation are necessary before the manuscript can be considered for publication.

1. **Overall focus and organization - The manuscript would benefit from a clearer focus. In several sections, side topics are discussed extensively without a direct connection to the central theme of the work. In particular, the discussion on the rifamycin-like PKS cluster appears largely outside the main scope of the manuscript, which should primarily concern the demiguisins and their biosynthesis.**

   Response: We thank the reviewer for this helpful suggestion. We agree that the discussion of the rifamycin-like PKS cluster was not central to the main focus of the manuscript, which is the discovery, structural characterization, and biosynthetic analysis of the demiguisins. In line with the reviewer's recommendation, also above, we have removed the section on the rifamycin biosynthetic gene cluster from the revised manuscript to improve focus and overall coherence.

2. **Feather-based media and keratin degradation - I do not clearly understand the connection between:**
   feather-based media,
- **keratin hydrolysis,**
- **keratinases**
- **and demiguisin biosynthesis.**

   iii. The manuscript includes an extended discussion on kerA/kerC-like genes and keratin degradation, but the relevance of these observations to secondary metabolite biosynthesis remains speculative and experimentally unsupported. In addition, the concept of "feather-based media" should be explained more clearly for the reader. At present, it is not obvious whether these conditions are biologically meaningful for metabolite production or simply intended to mimic the ecological niche of the bird-associated strain.

Response: Thank you for this constructive comment. We agree that the connection between feather-based media, keratin degradation, keratinases, and demiguisin biosynthesis was not sufficiently clearly articulated in the original version.

In the revised manuscript, we have clarified that the analysis of keratinase-encoding genes (KerA- and KerC-like subtilisin proteases) was not intended to directly link keratin degradation to demiguisin biosynthesis, but rather to assess the ecological and physiological potential of the strains to utilize keratin as a nitrogen-rich substrate, and by this contribute to metabolite production as well as triggering of potentially cryptic metabolites as a side effect. In this context, feather-based media serves as a defined keratin-rich growth condition, where intact feathers represent a highly recalcitrant form of β-keratin requiring active keratinolytic enzymes for degradation, whereas feather meal provides partially hydrolysed keratin peptides that are more readily accessible.

Accordingly, we have repositioned and restructured this section to make clear that the presence of keratinase homologues was evaluated as an indicator of the ability of the strains to grow on keratin-containing media, which may in turn support secondary metabolite production under nutrient-limited conditions. This analysis is therefore ecological and physiological in scope, rather than a direct mechanistic link to demiguisin biosynthesis. We have revised the text (to improve clarity and avoid overstating any direct connection between keratin degradation pathways and metabolite biosynthesis).

3. **Structural characterization of the minor compounds - Except perhaps for compound 2, the structural characterization of the minor congeners appears rather limited and, in some cases, not sufficiently rigorous. In particular:**
   • a clear carbon numbering scheme for the demiguisins is missing;
   • key NMR correlations supporting the peptide backbone assembly are not adequately presented;
   • several assignments appear to rely mainly on HRMS/MS data and biosynthetic analogy. More complete HMBC/COSY/ROESY information and a clearer discussion of the structural assignments would significantly strengthen this section.

4. **Relative and absolute stereochemistry - The stereochemical discussion is rather weak for peptide natural products of this type. The manuscript essentially assumes identical stereochemistry for all derivatives based on BGC homology and the absence of epimerase domains, but this alone is not sufficient to support the absolute stereochemical assignments. Given the peptidic nature of these compounds, one would expect:**
   • complete and clearly discussed Marfey analyses,
   • stronger ROESY/J-based analysis,
   • or more convincing biosynthetic evidence.
   In addition, the relative stereochemistry of the hydroxylated Piz residues is barely discussed.

Response: Many thanks for pointing out these analytical challenges. We have now included atom numbering for compound **2** in Figure 5, which is representative of compounds **1–6**.
The structures of the minor compounds were assigned based on a combination of high-resolution MS/MS fragmentation patterns, key 1D and 2D NMR correlations where available (including COSY and HMBC), and comparison of chemical shifts with the fully characterized major congeners, compounds **1** and **2**, within the demiguisin scaffold. Please see here the comparative NMR Tables and HRMS/MS analyses within the Supporting Information.

The absolute and relative stereochemistry of demiguisin A was previously established using Marfey's analysis and ROESY NMR experiments (DOI: 10.1038/s41467-024-52316-3; and referenced within the manuscript). However, it should be noted that the peptide contains several non-canonical highly modified peptides, thus Marfey's analysis remains limited in its interpretation. Although we attempted to crystallize compounds **1** and **2**, no crystals suitable for conclusive diffraction analysis were obtained. Given that all demiguisin derivatives are produced by a conserved NRPS assembly line lacking epimerase domains and exhibit highly consistent NMR shift patterns, biosynthetic logic, and fragmentation profiles, we assigned their stereochemistry by analogy to the previously characterized scaffold.

5. **Hydroxylated Piz biosynthesis - Although the section title suggests mechanistic insight, the manuscript does not clearly explain the origin of the different hydroxylation patterns observed in the Piz residues. The proposed involvement of DemAA3/DmgAA3 and/or the CYP450 enzyme remains highly speculative and is not supported by genetic or biochemical evidence. A clearer biosynthetic scheme for hydroxylated Piz formation would be very useful, together with a more detailed discussion of β-OH versus γ-OH Piz formation, sequence of hydroxylation, and comparison with known piperazic acid biosynthetic pathways.**

   Response: We thank the reviewer for this insightful comment. The proposed biosynthetic pathway for piperazic acid (Piz) has been described previously by our research group (DOI: 10.1038/s41467-024-52316-3). In addition, a comprehensive review of the biosynthesis and tailoring of Piz-containing natural products by Morgan et al. (DOI: 10.1039/c8np00076j) is cited in the manuscript. Importantly, while the core *dmg* biosynthetic gene cluster was experimentally validated through gene knockout studies, the functions of the proposed tailoring enzymes are currently inferred from sequence similarity searches and comparative sequence analyses. We acknowledge that the associated transformations are complex and may not be immediately apparent to readers. To improve clarity and facilitate discussion of the proposed biosynthetic steps, we have revised Figure 5 to include representative examples of related enzymatic transformations reported in the literature.

6. **Biological activity - The antimycobacterial activity is interesting, particularly against *Mycobacterium intracellulare*, but the biological evaluation remains relatively limited. A more detailed discussion of possible structure–activity relationships, especially concerning the role of the terminal Gly residue, would improve the biological part of the manuscript.**

   Response: Thank you for this suggestion. We agree that a more detailed discussion of the preliminary structure–activity relationships strengthen the biological evaluation of the manuscript. We have therefore expanded the discussion to more clearly address the apparent contribution of the terminal Gly residue to antimycobacterial activity. In particular, compounds 1 and 2, which contain the Gly residue, exhibited the strongest activity against Mycobacterium intracellulare infection, whereas derivatives 4 and 6 lacking Gly showed substantially reduced activity, and compounds 3 and 5 were inactive. These observations suggest that the terminal Gly residue may play an important role in maintaining the bioactive conformation or physicochemical properties required for antimycobacterial activity. At the same time, the absence of detectable cytotoxicity toward the HepG2 cell line indicates that the Piz residue itself is unlikely to be responsible for the cytotoxic effects often associated with other Piz-containing natural products. We have incorporated this discussion into the revised manuscript to better contextualize the observed bioactivity trends and the potential SAR of the demiguisin family (lines 331-337).

We very much appreciate the reviewers time to carefully evaluate the manuscript and for their constructive feedback that helped to improve the manuscript. Please find comments addressing revision recommendations for the manuscript COMMSCHEM-26-0294A in a point-to-point response.

Reviewer 1 remarks:

My comments have been addressed.

Reviewer 2 remarks:

I co-reviewed this manuscript with one of the reviewers who provided the listed reports. This is part of the Communications Chemistry initiative to facilitate training in peer review and to provide appropriate recognition for Early Career Researchers who co-review manuscripts.

Reviewer 3 remarks:

Recommendation: Major Revision

The revised manuscript is substantially improved compared with the original submission. The authors have carefully addressed most of my previous comments, and the manuscript is now considerably more focused, better organized, and easier to follow. I particularly appreciate the removal of sections that were not directly relevant to the main objective and the improved discussion of the structural characterization and biosynthetic hypotheses. Overall, the work now represents a much stronger contribution.Nevertheless, I believe that a few important issues still deserve attention before the manuscript can be considered for publication.

Major comments

1. Further improvement of the manuscript presentation

Although the manuscript has been significantly improved, the document would still benefit from careful text editing. There are several typographical errors that should be corrected (for example, on page 27, line 571, "Fig. SXX" should probably refer to "Fig. S40"). In addition, the narrative occasionally remains somewhat fragmented, giving the impression that sections have been inserted or modified independently rather than fully integrated into a coherent story. A final editorial revision would greatly improve readability.

Response: Thank you for the suggestions. We have conducted an in-depth editorial revision throughout the manuscript to correct typographical errors and improve the flow of the narrative. Specifically, we have addressed the incoherence by moving the targeted mining for keratinolytic enzymes into the metabolomics section to interconnect the sections.

2. Relationship between keratin degradation and demiguisin biosynthesis

I still remain unconvinced that the keratinase description is sufficiently justified within the context of this manuscript. Although the authors have clarified that this part aims to provide

ecological context rather than directly explain demiguisin biosynthesis, the experimental data presented do not convincingly support a relationship between keratin utilization and production of the demiguisins.

The growth experiments on feather-containing media are interesting, but metabolite production is mainly discussed for PDB and PDA cultures. If the authors wish to maintain the ecological argument, they should also report secondary metabolite production, particularly demiguisins, under the feather-based media (FA and FMA). It would be very informative to perform the same GNPS/SIRIUS analysis under these conditions and determine whether keratin-rich substrates actually influence metabolite production or congener distribution.

Response: Thank you for the comment. The 'Evaluation of cultivation conditions on demiguisin production' section of the manuscript was revised to detail the production of demiguisin on the feather media, as well as to more clearly highlight the GNPS analysis of all five media types detailed in the Supplementary Figures.

Moreover, while the manuscript compares the ability of the two strains to grow on feather-derived substrates, no direct measurement of keratin degradation or keratinase activity is presented. Therefore, the conclusion that one strain possesses a superior keratinolytic capacity remains largely indirect.

Response: For this revision, we conducted additional keratin degradation assays to validate the conclusions of superior keratinolytic capacity of the bird-derived strain compared to the soil-derived strain. The data was included in the supplementary data, and included in the manuscript.

3. Use of the term "metabolomics"

From a methodological point of view, I am not convinced that the work actually performs a metabolomic analysis in the strict sense. Rather, the study consists of MS-based molecular annotation using GNPS, SIRIUS and database-assisted dereplication. I would encourage the authors to adopt more precise terminology throughout the manuscript and avoid referring to this approach simply as "metabolomics."

Response: The full metabolomics analysis was conducted with the four types of media for both strains (Figure S3), and this has been clarified in the results and discussion section. However, the authors agree that majority of the manuscript has focused on targeted metabolomics or metabolomic profiling of specifically demiguisins, and thus this has been revised in the manuscript in several occasions.

4. Structural characterization

The structural characterization has been substantially improved and is now considerably more convincing. Likewise, the discussion concerning the origin of the different demiguisin congeners remains partly speculative, but the authors clearly acknowledge these limitations. I also appreciate that the stereochemical assignments are now explicitly presented as being based on comparison with the known compound together with biosynthetic considerations, which is a more balanced and scientifically rigorous presentation.

Response: Thank you for the positive feedback.

5. Biological activity and ecological interpretation

The biological section has also improved, but I still find the discussion only partially convincing.

Overall, the demiguisins display relatively modest antimicrobial activity, with rather high MIC values against most tested microorganisms. The only particularly interesting result is the activity against Mycobacterium intracellulare. However, the manuscript does not report the activity of the other major secondary metabolites produced by *Amycolatopsis* sp. PS_44_ISF1 and NBC_01488 (for example, the rifamycin congeners or the pachycephalamides) against this microorganism. Without these comparisons, it is difficult to evaluate whether demiguisins truly represent the biologically relevant antimicrobial metabolites produced by these strains.

Similarly, although the authors discuss the possible ecological relevance of the compounds in the context of avian-associated mycobacteria, I could not find data on the activity of the demiguisins against Mycobacterium avium subsp. avium, which is cited in the manuscript as the major causative agent of avian tuberculosis. These data would considerably strengthen the ecological interpretation. In their absence, the discussion should be moderated further.

Overall, I believe that additional experimental evidence or a more cautious interpretation is necessary before meaningful conclusions regarding the antimicrobial and ecological significance of these compounds can be drawn.

Response: Thank you for these helpful suggestions. We previously undertook extensive upscaling efforts to isolate the rifamycin derivatives for comparative antimicrobial testing. However, these compounds were present at substantially lower abundance in the extracts than the demiguisins. Despite repeated attempts using cultures at scales exceeding 10 L, we were unable to obtain sufficient quantities of analytically pure material for biological evaluation or complete structural elucidation. However, we would also like to clarify that rifamycin and congeners are known to be active against ***Mycobacterium tuberculosis*** and certain non-tuberculous mycobacteria by blocking bacterial transcription. Rifampicin, a semi-synthetic variant is recommended for the treatment of ***Mycobacterium avium*** complex pulmonary disease. As suggested we have now included this information into the revision.

Additionally, pachycephalamides A–F were previously tested against a panel of test strains, including ***Mycobacterium vaccae*** 10670 (Mv) (see reference Seibel et al.). Pachycephalamide F showed weak activity against ***M. vaccae***. Although we acknowledge that activity against other ***Mycobacterium*** species may be species-specific, the results of the previous testing were not sufficiently encouraging to justify further evaluation of these compounds. Moreover, the previously reported assessment of the interaction between demiguisin A and the pachycephalamides did not reveal enhanced activity. We have now included this information in the revised manuscript to provide a more complete and coherent account of the available data.

We would further like to clarify that our experiments were not intended to demonstrate greater antimicrobial activity of the demiguisins relative to other secondary metabolites produced by the same strains, such as antifungal macrolactams, rifamycins, or pachycephalamides, but a selective activity was performed due to the reported actvity of Piz-containing natural products. Therefore, we aim to highlight the additional activity observed for the demiguisins against the two mycobacterial pathogens investigated in this study.

The complete panel of bacterial strains, including the ***M. avium*** ATCC 700898 (MAC101) reference strain, used in the herein presented study is provided in Table S10 and offers a relevant initial indication of activity against the ***M. avium*** complex. As no activity was observed against this reference strain, and given the limited availability of the compounds, we did not pursue further testing against additional ***Mycobacterium avium*** subsp. ***avium*** strains.

Overall, we have revised the manuscript to ensure that our interpretation remains appropriately cautious. Specifically, we present the findings as suggestive of potential ecological roles rather than implying that the demiguisins are directly involved in avian host defense.

We very much appreciate the reviewers time to carefully evaluate the manuscript and for their constructive feedback that helped to improve the manuscript. Please find comments addressing revision recommendations for the manuscript COMMSCHEM-26-0294A in a point-to-point response.

Reviewer 1 remarks:

My comments have been addressed.

Reviewer 2 remarks:

I co-reviewed this manuscript with one of the reviewers who provided the listed reports. This is part of the Communications Chemistry initiative to facilitate training in peer review and to provide appropriate recognition for Early Career Researchers who co-review manuscripts. The improved version of the manuscript by Decker et al. has undergone extensive revision following the first round of peer review. The authors have sufficiently addressed all the points raised.

I still have reservations regarding the presence of sections that are disconnected from the original aim; however, the authors have significantly reduced these parts and improved the overall flow of the manuscript. Therefore, given that the authors consider these sections necessary to validate their hypothesis (and in the absence of substantial errors) I believe it is reasonable to accept the manuscript in its current version.

Response: Thank you for the time and effort to help improving our manuscript. As stated in detail in the responses to reviewer 3, we have further revised the narrative and refined the statements to improve the readability. For details, please see responses to reviewer 3.

Regarding the biosynthetic logic underlying the alternative enzymatic steps involved in demiguisin derivatives, the citation of Seibel et al. (2024; DOI: 10.1038/s41467-024-52316-3) partially explains why this aspect was not evaluated in the present manuscript. However, for the purposes of the peer-review process the authors should provide (at least, partially) details on their hypothesis concerning this alternative biosynthetic pathway. To this end, the authors should draw upon both past and current data to improve the present manuscript

Response: Thank you for the time and effort to help improving our manuscript. We refined the statement within the biosynthesis section and carefully discussed the current knowledge within the literature and included further insilco studies as well as a concluding statement.

Reviewer 3 remarks:

Recommendation: Major Revision

The revised manuscript is substantially improved compared with the original submission. The authors have carefully addressed most of my previous comments, and the manuscript is now considerably more focused, better organized, and easier to follow. I particularly appreciate the removal of sections that were not directly relevant to the main objective and the improved discussion of the structural characterization and biosynthetic hypotheses. Overall, the work now represents a much stronger contribution.Nevertheless, I believe that a few important issues still deserve attention before the manuscript can be considered for publication.

Major comments

1. Further improvement of the manuscript presentation

Although the manuscript has been significantly improved, the document would still benefit from careful text editing. There are several typographical errors that should be corrected (for example, on page 27, line 571, "Fig. SXX" should probably refer to "Fig. S40"). In addition, the narrative occasionally remains somewhat fragmented, giving the impression that sections have been inserted or modified independently rather than fully integrated into a coherent story. A final editorial revision would greatly improve readability.

Response: Thank you for the suggestions. We have conducted an in-depth editorial revision throughout the manuscript to correct typographical errors and improve the flow of the narrative. Specifically, we have addressed the incoherence by moving the targeted mining for keratinolytic enzymes into the metabolomics section to interconnect the sections.

2. Relationship between keratin degradation and demiguisin biosynthesis

I still remain unconvinced that the keratinase description is sufficiently justified within the context of this manuscript. Although the authors have clarified that this part aims to provide ecological context rather than directly explain demiguisin biosynthesis, the experimental data presented do not convincingly support a relationship between keratin utilization and production of the demiguisins.

The growth experiments on feather-containing media are interesting, but metabolite production is mainly discussed for PDB and PDA cultures. If the authors wish to maintain the ecological argument, they should also report secondary metabolite production, particularly demiguisins, under the feather-based media (FA and FMA). It would be very informative to perform the same GNPS/SIRIUS analysis under these conditions and determine whether keratin-rich substrates actually influence metabolite production or congener distribution.

Response: Thank you for the comment. The 'Evaluation of cultivation conditions on demiguisin production' section of the manuscript was revised to detail the production of demiguisin on the feather media, as well as to more clearly highlight the GNPS analysis of all five media types detailed in the Supplementary Figures.

Moreover, while the manuscript compares the ability of the two strains to grow on feather-derived substrates, no direct measurement of keratin degradation or keratinase activity is presented. Therefore, the conclusion that one strain possesses a superior keratinolytic capacity remains largely indirect.

Response: For this revision, we conducted additional keratin degradation assays to validate the conclusions of superior keratinolytic capacity of the bird-derived strain compared to the soil-derived strain. The data was included in the supplementary data, and included in the manuscript.

3. Use of the term "metabolomics"

From a methodological point of view, I am not convinced that the work actually performs a metabolomic analysis in the strict sense. Rather, the study consists of MS-based molecular annotation using GNPS, SIRIUS and database-assisted dereplication. I would encourage the authors to adopt more precise terminology throughout the manuscript and avoid referring to this approach simply as "metabolomics."

Response: The full metabolomics analysis was conducted with the four types of media for both strains (Figure S3), and this has been clarified in the results and discussion section. However, the authors agree that majority of the manuscript has focused on targeted metabolomics or metabolomic profiling of specifically demiguisins, and thus this has been revised in the manuscript in several occasions.

4. Structural characterization

The structural characterization has been substantially improved and is now considerably more convincing. Likewise, the discussion concerning the origin of the different demiguisin congeners remains partly speculative, but the authors clearly acknowledge these limitations. I also appreciate that the stereochemical assignments are now explicitly presented as being based on comparison with the known compound together with biosynthetic considerations, which is a more balanced and scientifically rigorous presentation.

Response: Thank you for the positive feedback.

5. Biological activity and ecological interpretation

The biological section has also improved, but I still find the discussion only partially convincing.

Overall, the demiguisins display relatively modest antimicrobial activity, with rather high MIC values against most tested microorganisms. The only particularly interesting result is the activity against Mycobacterium intracellulare. However, the manuscript does not report the activity of the other major secondary metabolites produced by *Amycolatopsis* sp. PS_44_ISF1 and NBC_01488 (for example, the rifamycin congeners or the pachycephalamides) against this microorganism. Without these comparisons, it is difficult to evaluate whether demiguisins truly represent the biologically relevant antimicrobial metabolites produced by these strains.

Similarly, although the authors discuss the possible ecological relevance of the compounds in the context of avian-associated mycobacteria, I could not find data on the activity of the demiguisins against Mycobacterium avium subsp. avium, which is cited in the manuscript as the

major causative agent of avian tuberculosis. These data would considerably strengthen the ecological interpretation. In their absence, the discussion should be moderated further.

Overall, I believe that additional experimental evidence or a more cautious interpretation is necessary before meaningful conclusions regarding the antimicrobial and ecological significance of these compounds can be drawn.

Response: Thank you for these helpful suggestions. We previously undertook extensive upscaling efforts to isolate the rifamycin derivatives for comparative antimicrobial testing. However, these compounds were present at substantially lower abundance in the extracts than the demiguisins. Despite repeated attempts using cultures at scales exceeding 10 L, we were unable to obtain sufficient quantities of analytically pure material for biological evaluation or complete structural elucidation. However, we would also like to clarify that rifamycin and congeners are known to be active against ***Mycobacterium tuberculosis*** and certain non-tuberculous mycobacteria by blocking bacterial transcription. Rifampicin, a semi-synthetic variant is recommended for the treatment of ***Mycobacterium avium*** complex pulmonary disease. As suggested we have now included this information into the revision.

Additionally, pachycephalamides A–F were previously tested against a panel of test strains, including ***Mycobacterium vaccae*** 10670 (Mv) (see reference Seibel et al.). Pachycephalamide F showed weak activity against ***M. vaccae***. Although we acknowledge that activity against other ***Mycobacterium*** species may be species-specific, the results of the previous testing were not sufficiently encouraging to justify further evaluation of these compounds. Moreover, the previously reported assessment of the interaction between demiguisin A and the pachycephalamides did not reveal enhanced activity. We have now included this information in the revised manuscript to provide a more complete and coherent account of the available data.

We would further like to clarify that our experiments were not intended to demonstrate greater antimicrobial activity of the demiguisins relative to other secondary metabolites produced by the same strains, such as antifungal macrolactams, rifamycins, or pachycephalamides, but a selective activity was performed due to the reported actvity of Piz-containing natural products. Therefore, we aim to highlight the additional activity observed for the demiguisins against the two mycobacterial pathogens investigated in this study.

The complete panel of bacterial strains, including the ***M. avium*** ATCC 700898 (MAC101) reference strain, used in the herein presented study is provided in Table S10 and offers a relevant initial indication of activity against the ***M. avium*** complex. As no activity was observed against this reference strain, and given the limited availability of the compounds, we did not pursue further testing against additional ***Mycobacterium avium*** subsp. ***avium*** strains.

Overall, we have revised the manuscript to ensure that our interpretation remains appropriately cautious. Specifically, we present the findings as suggestive of potential ecological roles rather than implying that the demiguisins are directly involved in avian host defense.

Please find comments addressing revision recommendations for the manuscript COMMSCHEM-26-0294C in a point-to-point response.

Reviewer #3 (Remarks to the Author):

The authors have satisfactorily addressed most of my previous concerns, particularly regarding structural characterization, the biosynthetic hypotheses, and the experimental support for keratin degradation. I therefore consider the manuscript suitable for publication.

A few minor issues remain, mainly about the strength and consistency of some statements across different sections. In particular, in my opinion, the proposed role of demiguisins in avian host defence remains speculative while the relationship between the glycine residue and antimycobacterial activity should not be presented as causal. These points do not require additional experiments, but would benefit from minor rewording to ensure that the conclusions remain fully consistent with the limitations appropriately acknowledged elsewhere in the revised manuscript. Specifically:

1) the Abstract should further moderate the proposed role of demiguisins in avian host defence;

The abstract has been updated to replace ‘potential roles in avian host defence’ with ‘promising anti-*Mycobacterium* activity’.

2) the Conclusion should avoid implying a causal dependence of antimycobacterial activity on the glycine residue.

Many thanks for the comment and have now avoided implying causal dependence and by this avoid any overstatement. The conclusions have been updated to 'The herein reported expansion of the demiguisin family further expands the chemical diversity of Piz-modifications and the overall Piz-containing peptides with promising antimycobacterial activity’.

In addition, the Results and Discussion/Methods should use the term “metabolomics” more precisely where the analysis is essentially targeted or comparative metabolite profiling.

The following sentences have been removed from the conclusion: Beyond the demiguisins, our study also establishes a foundation for exploring the co-secreted, species-specific metabolomic diversity of the soil-derived Amycolatopsis isolate.

‘Metabolomic’ was replaced with ‘comparative metabolite profiling’ in line 110.

Revision to

Title: Targeted Pathway Mining Reveals Novel Modified Hexapeptides from the Demiguisin Family

Authors: Tanya Decker, Marie Dayras, Lena Schweizer, Sari Rasheed, Franziska Fries, Bulisa Iova, Kasun H. Bodawatta, Knud Andreas Jønsson, Rolf Müller, Christine Beemelmanns.

Abstract Journal: Communication in chemistry

Manuscript Id: COMMSCHEM-26-0294

General remarks: The manuscript by Decker et al. investigates the **dem** biosynthetic gene cluster (BGC) from a soil-derived *Amycolatopsis* strain. This BGC corresponds to the previously described **dmg** cluster from an avian-associated strain. Using the corresponding BGC as bait, the authors identified a second producer, *Amycolatopsis* sp. NBC01488, from soil, whose cluster displayed high synteny with the original one despite the phylogenetic distance between the two strains. The authors selected combining targeted genome mining, comparative metabolomics, and structural elucidation as main experimental strategies. The authors identified six demiguisin congeners in total, including five previously undescribed derivatives, and linked their production to medium-dependent metabolic profiles. Interestingly, the study is methodologically strong integrating in-silico and wet-lab approaches into a single workflow.

Major points:

- The manuscript is very interesting but at present has different lacking points. The main weakness is that the proposed biosynthetic explanations remain largely hypothetical. The study also lacks genetic validation, such as targeted gene disruption, complementation, or heterologous expression. Overall, the paper is convincing at the level of discovery, but less definitive at the level of biosynthetic mechanism.

- The main findings of the manuscript are truly interesting and were obtained using the correct experimental pipeline. However, the current version of the manuscript is not consistent and confusing in some parts. Several sections of the manuscript appear disconnected from the overall context (see below). These sections stray from the authors' original objectives, which are currently not consistent with the presentation of the results.

- In my opinion, the authors have not fully described (or achieved) the aim of the manuscript "we hypothesized that the structural diversity within the demiguisin

compound family might be broader than originally described". The authors should focus on the true purpose of the manuscript - the comparison between BGC and an explanation of the biosynthetic capabilities of the selected microorganisms. At present, however, the manuscript consists of parts that are either completely or partially unrelated to its true purpose:

- Lines 120-159: Conditions included nutrient-rich and nutrient-poor media, such as peptone dextrose broth (PDB) and agar (PDA), M9 minimal medium (M9 broth), as well as habitat specific growth conditions using feather-based media, including cut pheasant feather agar (FA) and feather-meal agar (FMA). Here, it is worth noting that the bird-derived strain PS_44_ISF1 exhibited robust growth on both feather based substrates (FA and FMA), whereas the soil-derived strain NBC_01488 grew only on pre-processed FMA and failed to grow on non-processed pheasant feathers indicating differences within their encoded capacity to degrade biopolymers (Fig. 3A). In general, metabolites were extracted after 14 d using HP20 resin and partially purified on a solid-phase extraction (SPE) C18 column. Crude extracts were dissolved in MeOH and analyzed by LC–HRMS/MS in positive ionization mode. MS data were dereplicated using web-based Global Natural Products Social Molecular Networking (GNPS) V1.0, SIRIUS V6.1, and the Natural Product Atlas (NPAtlas) database, and visualized in Cytoscape V3.10.15,16,17,18 GNPS molecular networking revealed a diverse repertoire of secondary metabolites produced by both strains (PS_44_ISF1 and NBC_01488) when grown in PDB and PDA media, with dereplication identifying multiple annotated metabolites grouped into distinct molecular families (Fig. 3B). Overall, pronounced media-dependent differences in the metabolome were observed between PDB and PDA.

  Do the authors intend to investigate the overall metabolomic capabilities of the strains under study or do they want to focus on the BGCs related to demiguisin? At the moment, this section is too disconnected from the rest of the manuscript.

Lines 170-181: we mined the genomes for keratinases, commonly assigned as subtilisin-like serine proteases of the S8 family.Among these, KerA (GenBank ID:176 AAB34259.1) from *Bacillus licheniformis* and KerC (GenBank ID: ABY65903.1) from *Bacillus subtilis* are among the best-characterized keratinases and were used as reference sequences in a dedicated BLASTp –

Why do the authors move the focus from the biosynthesis of Demiguisin to keratinase family?

Lines 290 – 306: Intrigued by the presence of the antibiotic-acting rifamycin congeners, we pursued a clinker alignment analysis using the rifamycin reference rif BGC from A. mediterranei S699 with the rfm BGC identified in PS_44_ISF1 (Table S11, Fig. S38)22 to estimate its structural relatedness to previously reported pathways. Overall, an extensive conservation between the rif and rfm BGCs was

observed, including strong alignments across the type I polyketide synthase (PKS) genes (rifA–rifF), starter unit biosynthesis genes (rifG–rifI and rifK–rifN), and post-biosynthetic modification genes (rifS, rifT, and ORF4), which led us to conclude that the diversity of observed rifamycin congeners, while not yet annotated, likely encompass mostly structurally explored rifamycin congeners – Why the authors add this comparison rfm/rif BGC???

The manuscript should be thoroughly revised by the authors, focusing on their original objectives and avoiding the inclusion of information that, while certainly valid, is misleading in the context of the analysis comparing BGCs.

- The authors compare the BGCs in first part of the manuscript but at line 300 they compare the architecture of the BGC homolog *dem* (NBC_01488) and *dmg* (PS_44_ISF1) **<u>in greater detail.</u>** Maybe it would be better to present a single comparison of the BGCs rather than splitting them into one in "less detail" and another in "greater detail." The authors should reorganize the manuscript and revise it accordingly.

- The identification and characterization of the six demiguisin derivatives (678.3563, 637.3299, 621.3348, 621.3378, 605.3411 m/z) is very interesting, but the authors have provided few explanations (even speculating) regarding the alternative enzymatic steps for the biosynthesis of these products.

- Authors should improve the descriptions of the methods. Particularly authors should highlight all values of the parameters used for predictive analyses and bioinformatics mining.

- Conclusions are too generic, please improve

Minor point:

Lines 51-54 - *"Amycolatopsis* sp. PS_44_ISF1 derived from the UG of the toxic bird *Pachycephala schlegelii*, that bacterial metabolites such as the antifungal macrolactam ciromicin A,3,4 the co-secreted tensioactive lipopeptide family pachycephalamides, and a highly modified hexapeptide, named demiguisin A (**1**) can directly contribute to the bird's defense against microbial pathogens" – Please rephrase, missing verbs/confusing sentence.

Line 75 – "using a targeted nBLAST (NCBI)" - Please details what the authors mean with "targeted"?

Line 79 – "Detailed pairwise comparison using clinker (CAGECAT webserver) revealed extensive synteny and high sequence identity (>70%) across the core NRPS loci (Fig. 2A)" – Please add percentage identities in supplementary table.

in-house *Amycolatopsis* strains (M39 and DSM 44993) containing experimentally validated BGCs for comparison – please explain this choice (e.g. positive control)?

Authors should be more consistent along the entire manuscript descripting the similarities between BGCs :

Fore examples, in the first part of the manuscript "Although demiguisin BGCs, *dmg* (*Amycolatopsis* sp. PS_44_ISF1) and *dem* clusters (*Amycolatopsis* sp. NBC_01488), harbored similar core genes associated with amino acid biosynthesis and a NRPS assembly line, they did not cluster together in the BiG-SCAPE network under standard cutoffs (Fig. S2), likely due to variability in flanking or auxiliary genes outside the NRPS region (Fig. S1) – then "the minor detectable differences within the encoded demiguisin-related pathways were reflected"

Based on these evaluations the manuscript certainly represents a valuable contribution to Communication in Chemistry, but the present version appeared confusingly written and preliminary. My suggestion is to invite the authors to submit a new and revised version of the manuscript before proceeding with the peer-review process.

Rev #2 to

Title: Targeted Pathway Mining Reveals Novel Modified Hexapeptides from the Demiguisin Family

Authors: Tanya Decker, Marie Dayras, Lena Schweizer, Sari Rasheed, Franziska Fries, Bulisa Iova, Kasun H. Bodawatta, Knud Andreas Jønsson, Rolf Müller, Christine Beemelmanns.

Abstract Journal: Communication in chemistry

Manuscript Id: COMMSCHEM-26-0294

The improved version of the manuscript by Decker et al. has undergone extensive revision following the first round of peer review. The authors have sufficiently addressed all the points raised.

I still have reservations regarding the presence of sections that are disconnected from the original aim; however, the authors have significantly reduced these parts and improved the overall flow of the manuscript. Therefore, given that the authors consider these sections necessary to validate their hypothesis (and in the absence of substantial errors) I believe it is reasonable to accept the manuscript in its current version.

Regarding the biosynthetic logic underlying the alternative enzymatic steps involved in demiguisin derivatives, the citation of Seibel et al. (2024; DOI: 10.1038/s41467-024-52316-3) partially explains why this aspect was not evaluated in the present manuscript. However, for the purposes of the peer-review process the authors should provide (at least, partially) details on their hypothesis concerning this alternative biosynthetic pathway. To this end, the authors should draw upon both past and current data to improve the present manuscript.

Based on these considerations, the manuscript will be accepted for publication after minor revision.
