# Supplementary material for: Targeted pathway mining reveals novel modified hexapeptides from the demiguisin family
Source: Commun Chem. 2026 Oct 17;9:327. doi: 10.1038/s42004-026-02250-w (PMC13676617; doi:10.1038/s42004-026-02250-w)
Supplement: Supplementary file 2 [file 42004_2026_2250_MOESM2_ESM.pdf]

Supplementary Information

Targeted Pathway Mining Reveals Novel Modified Hexapeptides from the Demiguisin Family

Tanya Decker, [a] *Marie Dayras,* [a, b] *Lena Schweizer,* [a] *Sari Rasheed,* [a] *Franziska Fries,* [a,c] *Bulisa Iova,* [d] *Kasun H. Bodawatta,* [e] *Knud Andreas Jønsson,* [f] *Rolf Müller,* [a,c,g] *Christine Beemelmanns* *[a,g,h]

[a] *Helmholtz Institute for Pharmaceutical Research Saarland (HIPS), Campus E8.1, 66123 Saarbrücken, Germany*

[b] *Université d'Orléans, CNRS, ICOA, UMR 7311, Orléans, France*

[c] *German Center for Infection Research (DZIF), Partner Site Hannover-Braunschweig, 38124 Braunschweig, Germany*

[d] *PNG National Museum and Art Gallery, Port Moresby, Papua New Guinea*

[e] *Section for Molecular Ecology and Evolution, Globe Institute, University of Copenhagen, Copenhagen, Denmark*

[f] *Department of Bioinformatics and Genetics, Swedish Museum of Natural History, Stockholm, Sweden*

[g] *Pharma Science Hub, Saarland University, Campus E8.1, 66123, Saarbrücken, Germany*

[h] *Faculty of Medicine, Saarland University, Saarbrücken, Germany*

Table of Contents

1. Media and microbial strains 2
2. Whole genome analysis and genome mining 3
3. Metabolomics 7
4. NMR and MS/MS analysis 10
5. Analysis of MS/MS fragmentation pattern 38
6. Biological activity 44
7. Biosynthetic pathway analysis 46
8. References 49

1. Media and microbial strains

Table S1. Recipes of media used in this study. Prior to use, all media were autoclaved at 121 °C for 20 min.

Medium	Composition (per L)	Purpose	Company/ reference
PDB / PDA	26.5 g potato extract glucose broth, for PDB; 2% agar (w/v) for PDA	Metabolite production medium & co-cultivation assays	Carl Roth GmbH + Co. KG
M9 minimal medium	6 g Na_2HPO_4, 3 g KH_2PO_4, 1 g NH_4Cl, 0.5 g NaCl. Addition of sterile solutions: 1 mL of 1M $MgSO_4.7H_2O$, 10 mL of 20% Glucose, 100 µL of 0.4% thiamine HCl, 5 mL of 20% casamino acids	Metabolite production medium	1
Mueller Hinton broth/agar	21 g Mueller Hinton broth for MHB; 2% agar (w/v) for MHA	Antimicrobial activity assays	Difco Laboratories
Feather broth/ agar	0.5 g NH_4Cl, 0.5 g NaCl, 0.3 g K_2HPO_4, 0.4 g KH_2PO_4, 0.1 g $MgCl_2.6H_2O$, 10 g pheasant feathers (or duck feathers for Keratin azure assay), for FB; 2% agar (w/v) for FA.<br>Feather preparation: pheasant feathers were cut into pieces and washed twice with MeOH, then rinsed twice with deionised water. After drying they were cut into smaller pieces with a blender.	Eco-mimetic culture media; Keratin azure assay	2
Feather meal broth/ agar	0.5 g NH_4Cl, 0.5 g NaCl, 0.3 g K_2HPO_4, 0.4 g KH_2PO_4, 0.1 g $MgCl_2.6H_2O$, 10 g commercially available feather meal, for FMB; 2% agar (w/v) for FMA.	Eco-mimetic culture media; Keratin azure assay	2

Table S2. Strains used in this study. All strains are stored in glycerol stocks in the culture collection of the MICA department at HIPS, in 25% glycerol at −80 °C and were restreaked onto on peptone dextrose agar (PDA).

Strain	Isolation source	Use	Reference
Amycolatopsis sp. PS_44_ISF1	Uropygial gland (UG) of *Pachycephala schlegelii* from Papua New Guinean	Metabolite producer	3
Amycolatopsis sp. NBC_01488	Soil collected in Denmark	Metabolite producer	4
Amycolatopsis sp. M39	Fungal comb material of a *Macrotermes natalensis* colony in South Africa	Genome mining	5
Amycolatopsis saalfeldensis DSM 44993	Acidic and heavy-metal-containing rocks in the central grotto of the medieval alum slate mine Feengrotten in Germany	Genome mining	6
Streptomyces sp. M56	Fungal comb material of a *Macrotermes natalensis* colony in South Africa	Co-culture	5
Rhodococcus sp. Zoo49	Faecal sample of *Aldabrachelys gigantea* in Saarbrücken Zoo	Co-culture	7
Kocuria sp. LS23	UG of *Pachycephala schlegelii* in Papua New Guinean	Co-culture	8
Kocuria sp. LS19	UG of *Pachycephala schlegelii* in Papua New Guinean	Activity assay	8
Curtobacterium sp. LS33	UG of *Pachycephala schlegelii* in Papua New Guinean	Activity assay	8
Curtobacterium sp. LS34	UG of *Pachycephala schlegelii* in Papua New Guinean	Activity assay	8
Kocuria sp. LS38	UG of *Pachycephala schlegelii* in Papua New Guinean	Activity assay	8

2. Whole genome analysis and genome mining

Table S3. Genome accession numbers.

Strain	Accession number	Data	Reference
Amycolatopsis sp. PS_44_ISF1	JANUXO000000000	ONT, PB, I	3
Amycolatopsis sp. NBC_01488	CP109434.1	ONT and I	4
Amycolatopsis saalfeldensis DSM 44993	FOEF00000000	I	NCBI (reference genome)
Amycolatopsis sp. M39	LWSF00000000	I	9

ONT: Oxford Nanopore, PB: Pac Bio, I: Illumina

Table S4. GenBank assembly numbers used for fastANI analysis (data obtained from NCBI).

Strain	Accession number
Amycolatopsis sp. NBC_01488	GCA_036227105.1
Amycolatopsis sp. PS_44_ISF1	GCA_032335455.1
Amycolatopsis saalfeldensis DSM 44993	GCA_900110575.1
Amycolatopsis rhabdoformis NCIMB 14900	GCA_035918295.1
Amycolatopsis carbonis 2-15	GCA_030285625.1
Amycolatopsis sulphurea DSM 46092	GCA_002564045.1
Amycolatopsis benzoatilytica DSM 43387	GCA_000383915.1
Amycolatopsis albidoflava JCM 11300	GCA_039525725.1
Amycolatopsis echigonensis JCM 21831	GCA_014174495.1
Amycolatopsis nivea KTCT 39515	GCA_004522235.1
Amycolatopsis dendrobii DR6-1	GCA_014145675.1
Amycolatopsis melonis NEAU NG30	GCA_040064235.1
Amycolatopsis vastitatis H5	GCA_002234595.1
Amycolatopsis balhimycina DSM 44591	GCA_000384295.1
Amycolatopsis mediterranei ATCC 13685	GCA_000220945.1
Amycolatopsis tolypomycina DSM 44544	GCA_900105945.1
Amycolatopsis rifamycinica DSM 46095	GCA_000695625.1
Amycolatopsis solani MEP2-6	GCA_033441515.1
Amycolatopsis lexingtonensis DSM 44653	GCA_014873755.1
Amycolatopsis eburnean GLM-1T	GCA_003937945.1
Amycolatopsis australiensis DSM 44654	GCA_900119165.1
Amycolatopsis iheyensis OK19-0408	GCA_024703995.1
Amycolatopsis pretoriensis DSM 44654	GCA_900107925.1
Amycolatopsis mongoliensis 4-36	GCA_030285665.1
Amycolatopsis camponoti A23T	GCA_902497555.1
Amycolatopsis ponsaeliensis RTGN1	GCA_030182635.1

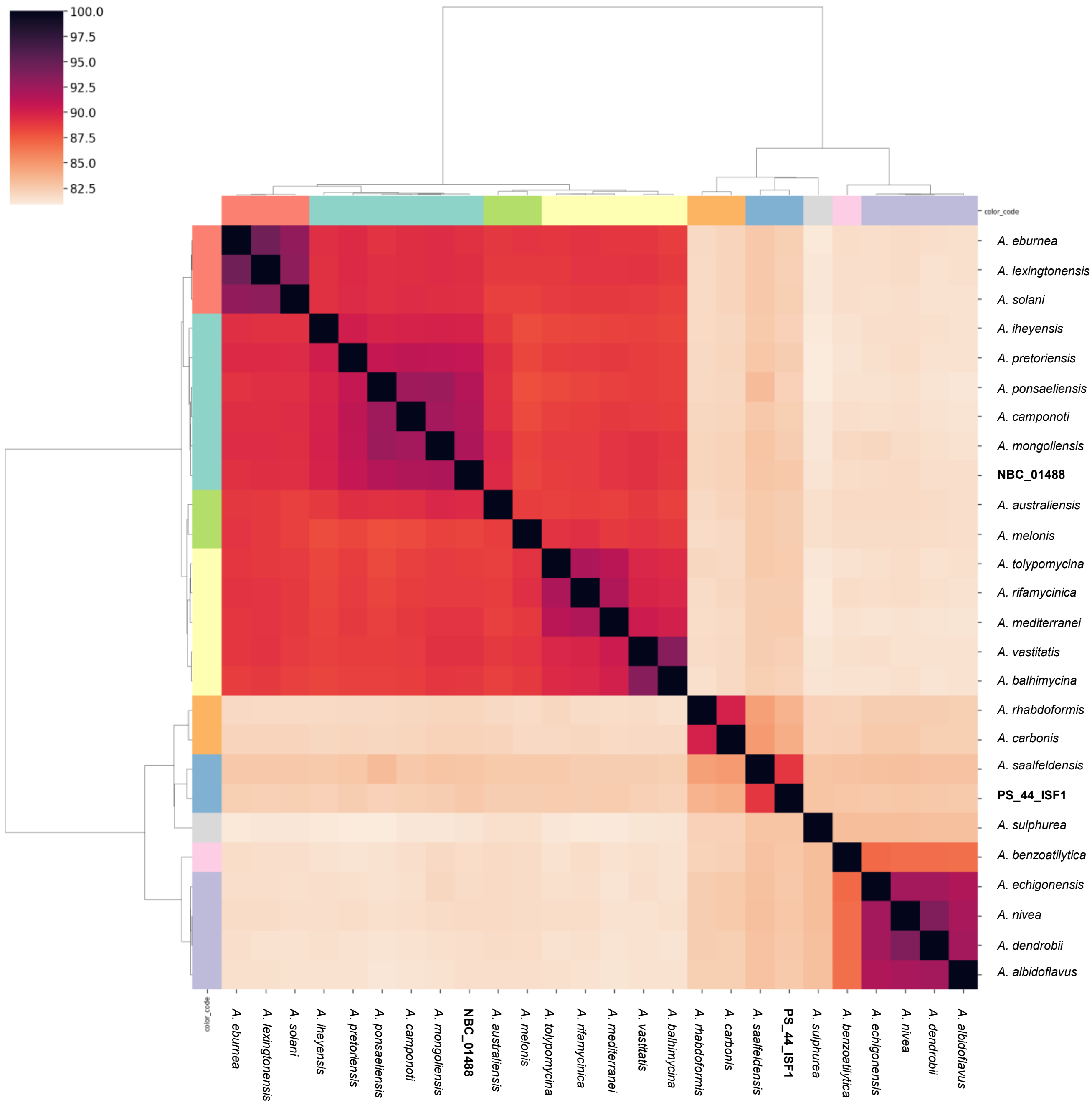

Figure S1. Heatmap of pairwise average nucleotide identity (ANI) values derived from fastANI analysis, showing the phylogenomic relationships among *Amycolatopsis* species. Darker colours represent higher ANI values, indicating closer genomic relatedness. Hierarchical clustering of both genomes and ANI values illustrates distinct species-level groupings, including the placement of *Amycolatopsis* sp. PS_44_ISF1 and *Amycolatopsis* sp. NBC_01488 relative to other *Amycolatopsis* taxa. Strains included in this analysis were obtained from the TYGS analysis (indicating the closest relative to the two strains).

A

	BGC count	NRPS	T1-PKS	NAPAA	RiPP	Terpene	T2-PKS	Other/hybrid
M39	38	6	2	2	4	4	2	18
PS_44_ISF1	35	7	2	3	1	1	0	21
NBC_01488	33	3	1	1	1	1	0	26
DSM 44993	36	3		2	4	2	0	25

B

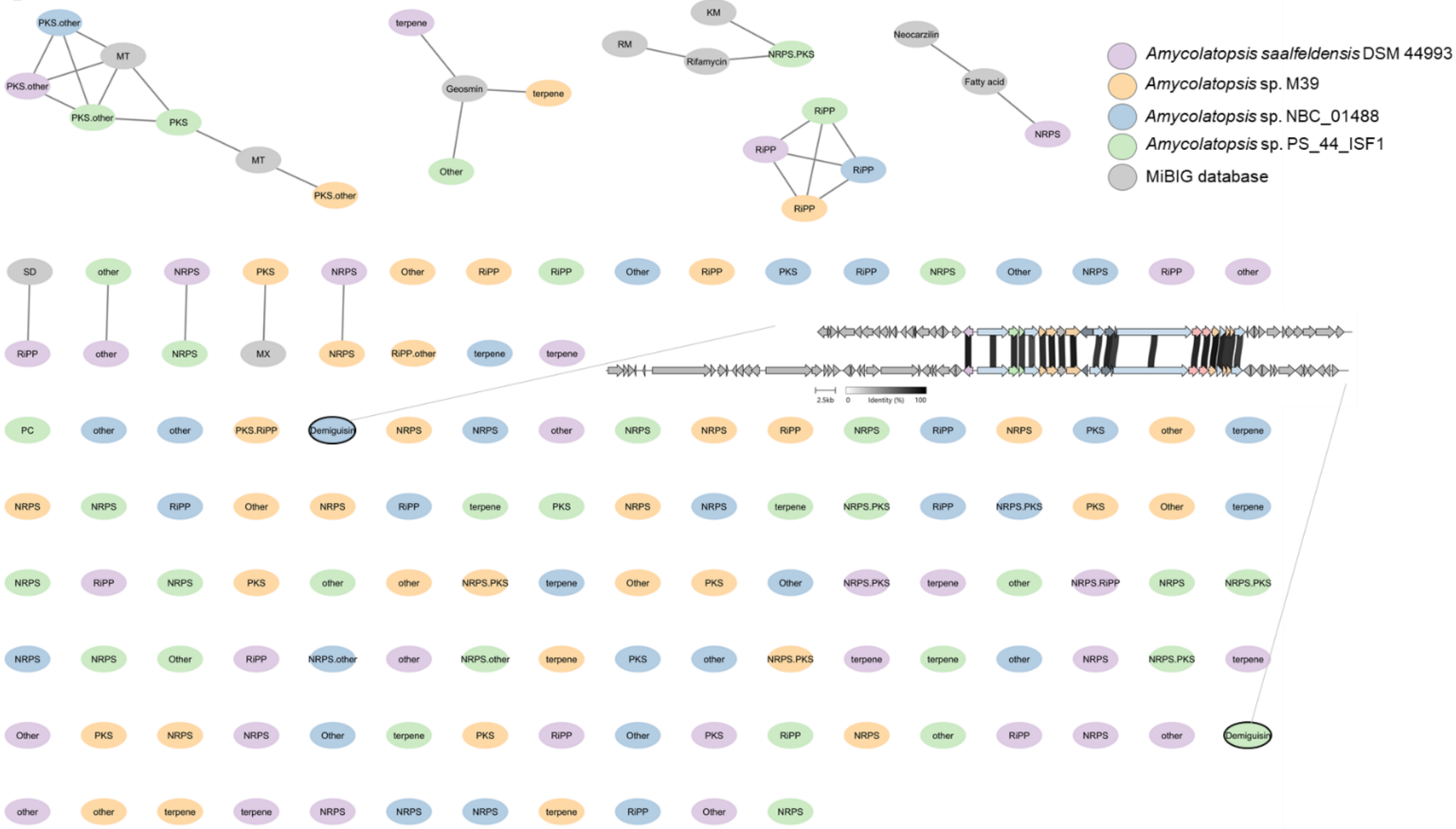

C

NRPS core genes (NBC_01488/PS_44_ISF1)	Sequence alignment (%)
demP1/dmgP1	85
demP2/dmgP2	91
demP3/dmgP3	83
demP4/dmgP4	81
demP5/dmgP5	85
demP6/dmgP6	88
demP7/dmgP7	80

Figure S2. **(A)** A summary table is provided for the antiSMASH-detected secondary metabolite classes in the genomes of these four strains. (B) The BiG-SCAPE network of BGCs present in the genomes of *Amycolatopsis* sp. PS_44_ISF1, *Amycolatopsis* sp. NBC_01488, *Amycolatopsis saalfeldensis* DSM 44993, and *Amycolatopsis* sp. M39, with reference BGCs incorporated from the MiBIG database. (C) The sequence similarity percentage between the core NRPS genes between the *dem* and *dmg* BGCs. Abbreviations include, MT: Macrotermycin; RM: Rifamorpholines; KM: Kanglemycin; PC: Pachycephalamides; SD: Saalfelduracin, MX: Mutaxanthene, PKS: Polyketide synthases, NRPS: Non-ribosomal peptide synthetases; RiPP: Ribosomally synthesized and posttranslationally modified peptides. The demiguisin BGCs present in PS_44_ISF1 and NBC_01488 were aligned using clinker (CAGECAT web tool), and the alignments of the complete BGC clusters are indicated.

Table S5. BLASTp cluster analysis of the *kerC* gene (Query ID: ABY65903.1) from *Amycolatopsis* sp. PS_44_ISF1 and NBC_01488.

Representative sequence	Max Score	Total Score	Query Cover	E value	Per. ident	Acc. Len	Accession
S8 family peptidase [*Amycolatopsis* sp. PS_44_ISF1]	113	113	98%	6.00E-28	27.9	482	WP_315660576.1
type VII secretion-associated serine protease mycosin [*Amycolatopsis* sp. PS_44_ISF1]	109	109	70%	6.00E-27	34.16	442	WP_315656874.1
S8 family peptidase [*Amycolatopsis* sp. PS_44_ISF1]	107	107	92%	5.00E-26	28.54	468	WP_315653680.1
S8 family peptidase [*Amycolatopsis solani*]	102	102	69%	2.00E-24	29.57	464	WP_290057759.1
S8 family peptidase [*Amycolatopsis mongoliensis*]	102	102	65%	2.00E-24	33.01	483	WP_285998519.1
S8 family peptidase [*Amycolatopsis* sp. NBC_01488]	85.9	85.9	61%	3.00E-19	33.76	295	WP_329056513.1
S8 family serine peptidase [*Amycolatopsis* sp. PS_44_ISF1]	74.7	74.7	56%	1.00E-14	31.3	1067	WP_315660536.1
S8 family serine peptidase [*Amycolatopsis* sp. NBC_01488]	73.2	73.2	61%	2.00E-14	25.07	567	WP_329056738.1
S8 family serine peptidase [*Amycolatopsis* sp. NBC_01488]	68.2	68.2	56%	1.00E-12	33.21	1078	WP_329044174.1
S8 family peptidase [*Amycolatopsis* sp. NBC_01480]	53.5	97.8	76%	5.00E-08	25.51	1113	WP_329057526.1
S8 family peptidase [*Amycolatopsis* sp. CA-126428]	52.4	99	68%	1.00E-07	24.65	1130	WP_199191060.1
S8 family serine peptidase [*Amycolatopsis* sp. NBC_01488]	38.9	77	61%	0.002	29.56	542	WP_329052424.1

Table S6. BLASTp cluster analysis of the *kerA* gene (Query ID: AAB34259.1) from *Amycolatopsis* sp. PS_44_ISF1 and NBC_01488.

Representative sequence	Max Score	Total Score	Query Cover	E value	Per. ident	Acc. Len	Accession
type VII secretion-associated serine protease mycosin [*Amycolatopsis* sp. PS_44_ISF1]	122	122	65%	1.00E-31	37.12	442	WP_315656874.1
S8 family peptidase [*Amycolatopsis* sp. PS_44_ISF1]	118	118	83%	6.00E-30	32.18	468	WP_315653680.1
S8 family peptidase [*Amycolatopsis solani*]	115	115	88%	5.00E-29	28.23	464	WP_290057759.1
S8 family peptidase [*Amycolatopsis* sp. PS_44_ISF1]	114	114	63%	2.00E-28	35.38	482	WP_315660576.1
S8 family peptidase [*Amycolatopsis mongoliensis*]	108	108	65%	3.00E-26	32.36	483	WP_285998519.1
S8 family peptidase [*Amycolatopsis* sp. NBC_01488]	84.7	84.7	60%	8.00E-19	32.77	295	WP_329056513.1
S8 family serine peptidase [*Amycolatopsis* sp. NBC_01488]	83.2	83.2	61%	1.00E-17	26.21	567	WP_329056738.1
S8 family serine peptidase [*Amycolatopsis* sp. PS_44_ISF1]	77	77	41%	1.00E-15	35.23	537	WP_315653771.1
S8 family serine peptidase [*Amycolatopsis* sp. NBC_01488]	56.2	96.3	60%	6.00E-09	33.33	542	WP_329052424.1
S8 family peptidase [*Amycolatopsis* sp. NBC_01480]	53.5	53.5	57%	6.00E-08	27.15	1113	WP_329057526.1
S8 family peptidase [*Amycolatopsis* sp. CA-126428]	45.4	45.4	57%	2.00E-05	25.52	1130	WP_199191060.1

3. Metabolomics

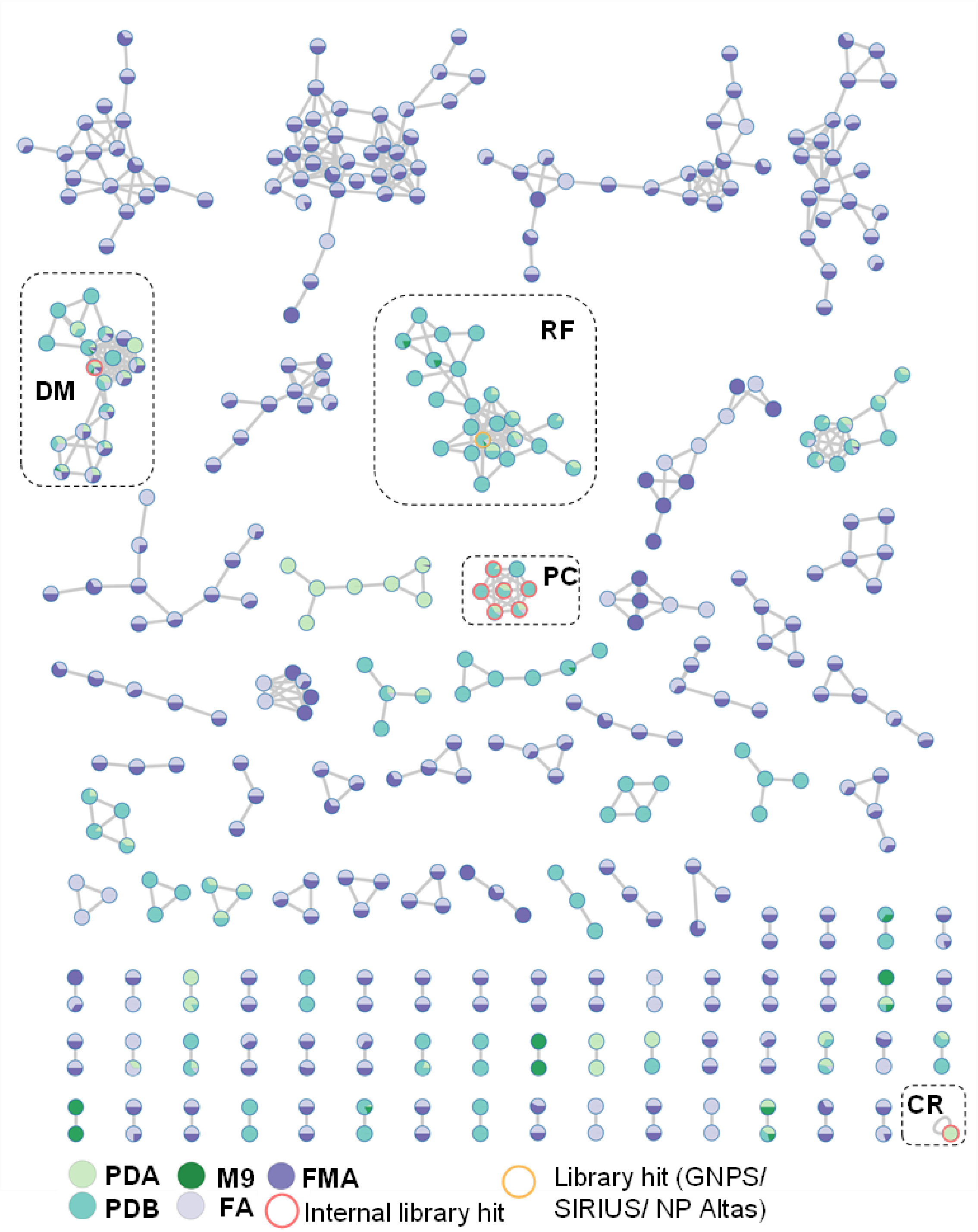

Figure S3. GNPS molecular network reveals metabolomic repertoire of *Amycolatopsis* sp. PS_44_ISF1 cultivated on PDA, PDB, M9, FMA, and FA for 14 days. Node colors indicate media type, and edge thickness reflects cosine similarity between MS/MS spectra.

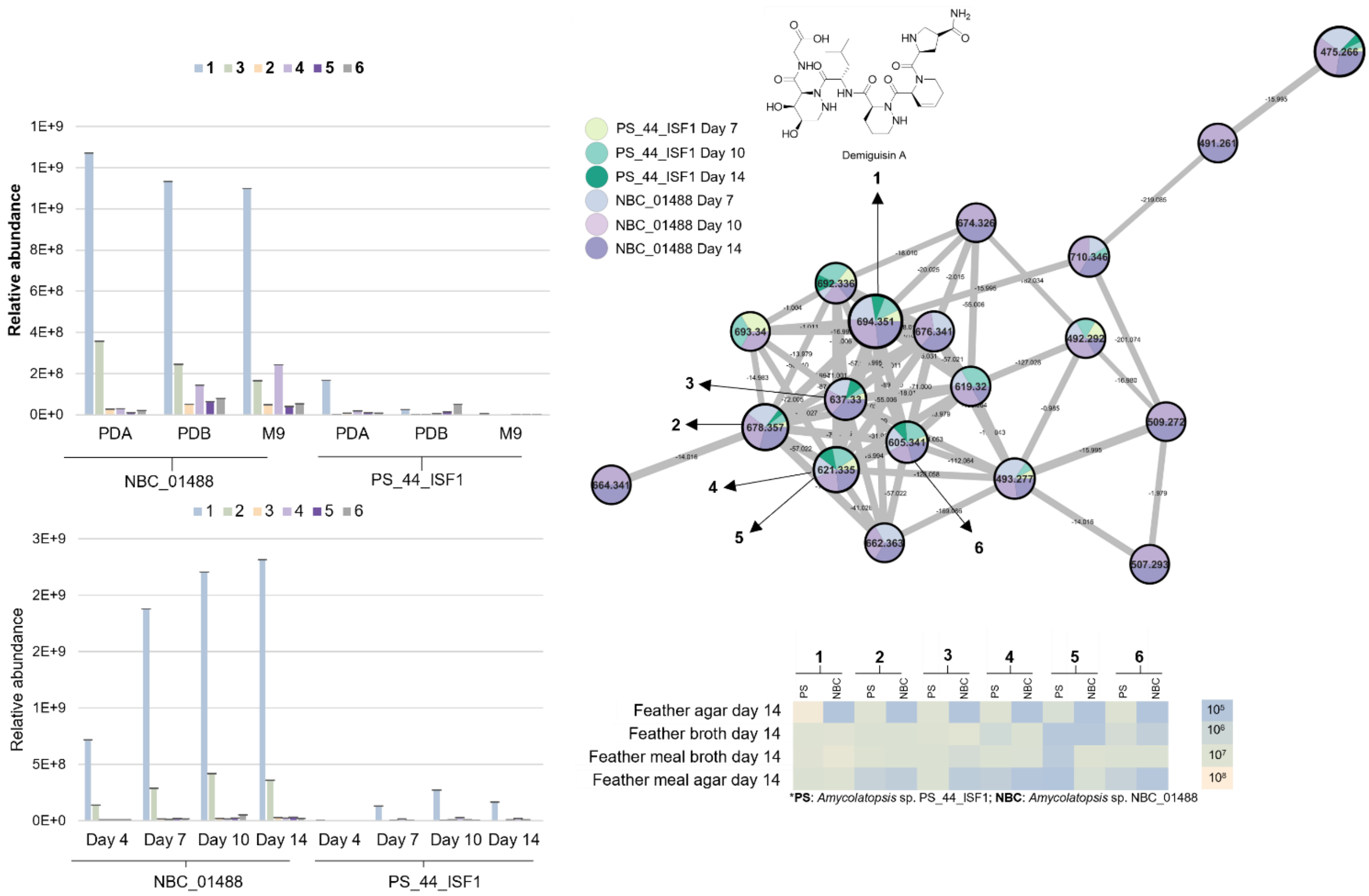

Figure S4. GNPS molecular network and relative abundance profiles of demiguisin derivatives (compounds **1**–**6**) produced by *Amycolatopsis* sp. PS_44_ISF1 and *Amycolatopsis* sp. NBC_01488 cultivated on PDA for 4, 7, 10, and 14 days. Left: Relative abundance of each demiguisin analogue across the cultivation time course for both strains on PDA (bottom), showing strain-specific production patterns and temporal changes in metabolite accumulation as well as relative abundance changes dependent on media alternations at 14 days (top). The relative abundance of the demiguisin derivatives (compounds **1**–**6**) showing strain-specific production patterns when grown on feather agar, feather broth, feather meal agar or feather meal broth after 14 days (bottom). Right: GNPS molecular network (top) highlighting nodes corresponding to demiguisin A and related analogues (**1**–**6**). Node colors indicate strain and cultivation time, and edge thickness reflects cosine similarity between MS/MS spectra.

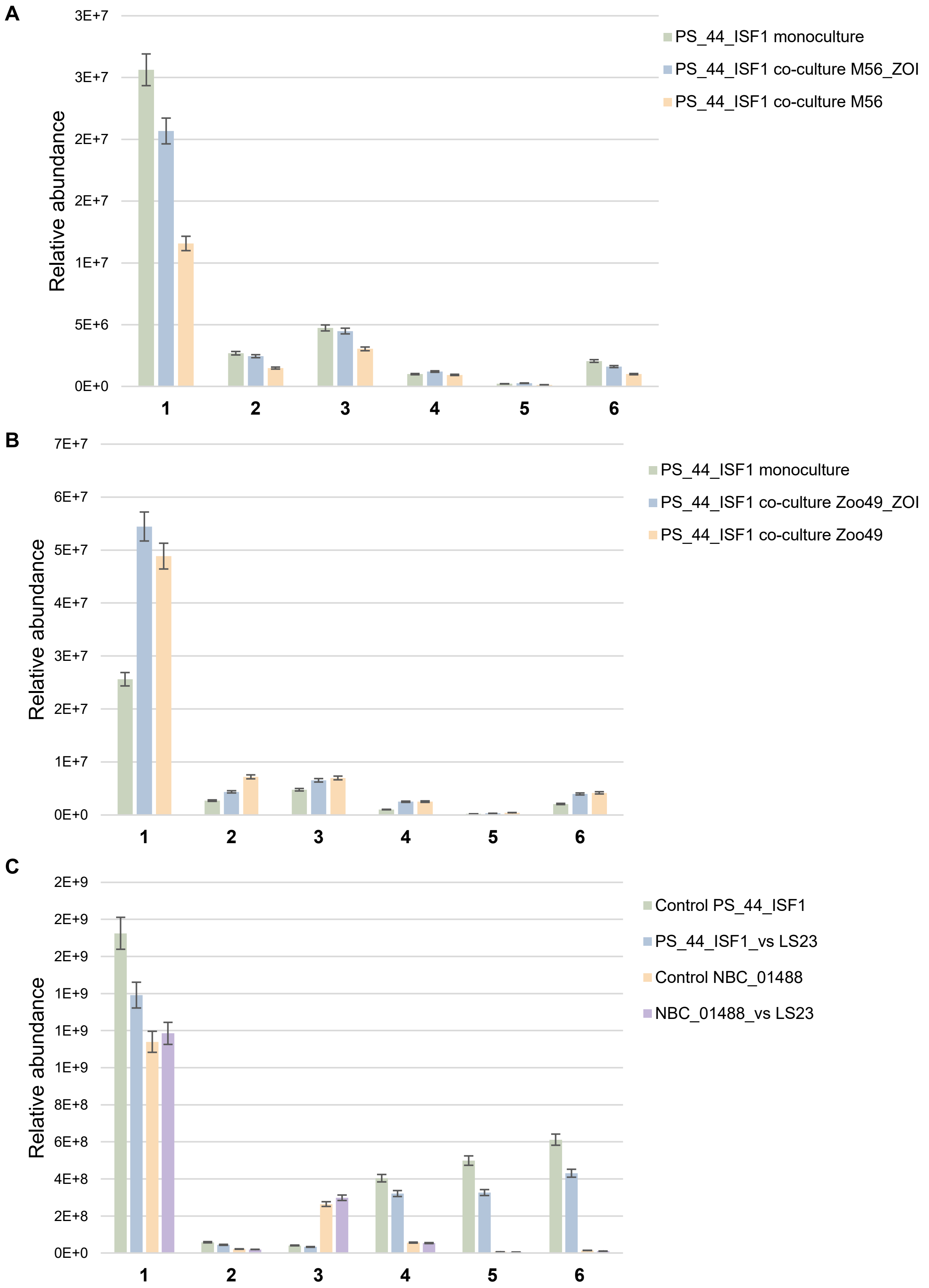

Figure S5. Relative abundance of demiguisins (compounds **1**-**6**) in co-culture with A) *Streptomyces* sp. M56, B) *Rhodococcus* sp. Zoo49 on PDA and C) *Kocuria* sp. LS23. ZOI: Zone of inhibition.

4. NMR and MS/MS analysis

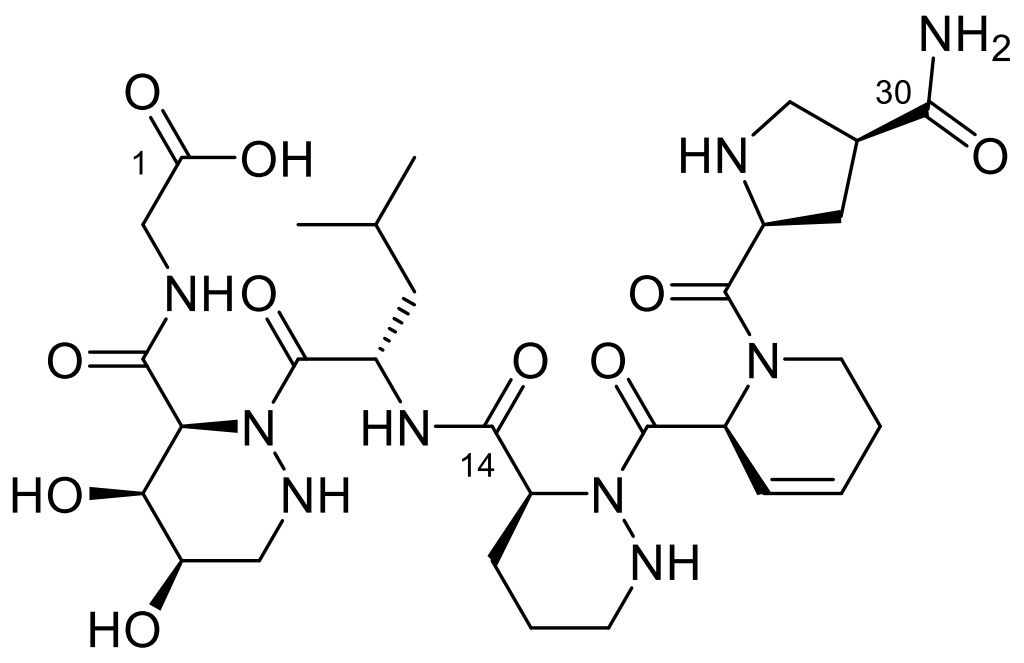

Demiguisin A (**1**)

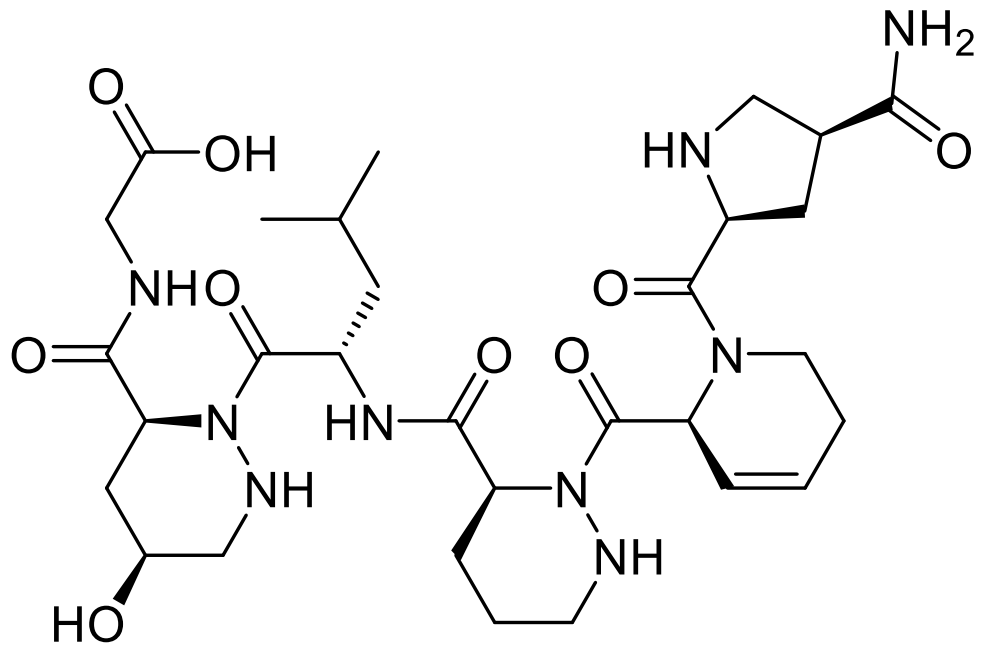

Demiguisin B (**2**)

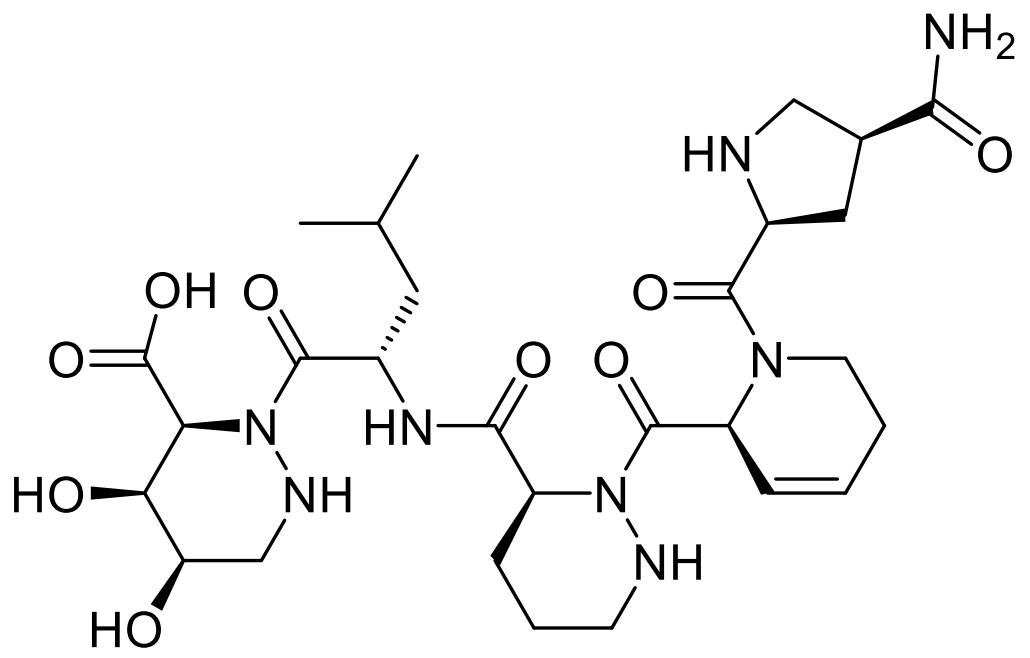

Demiguisin C (**3**)

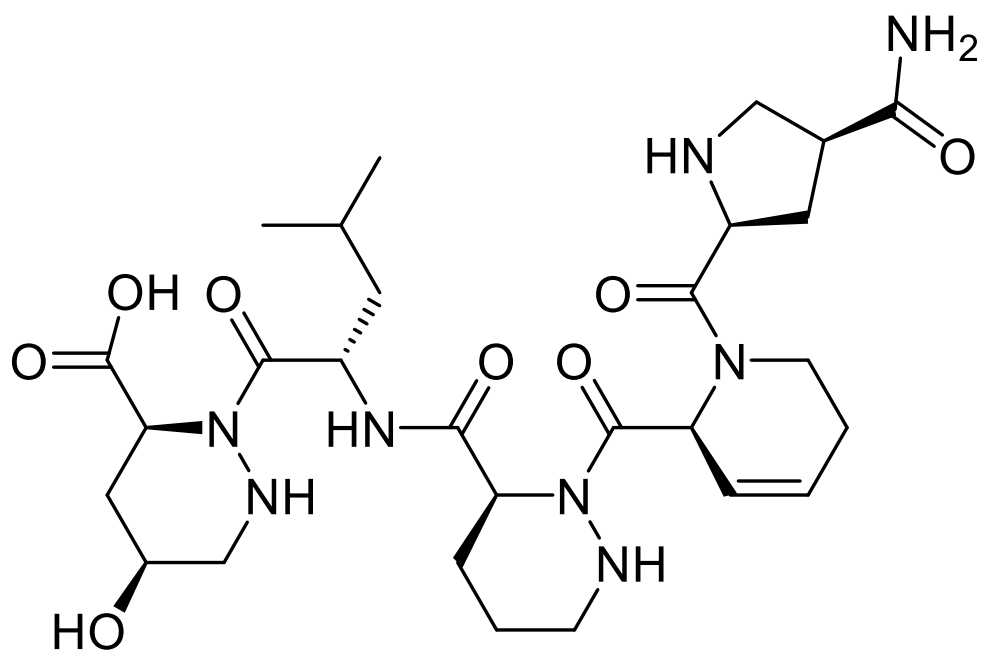

Demiguisin D (**4**)

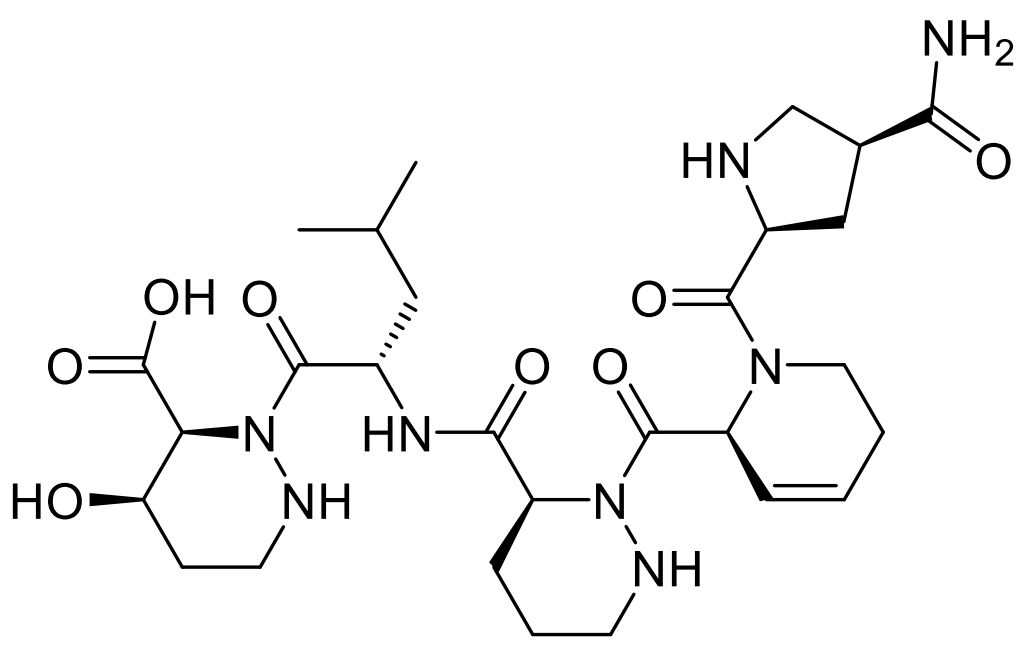

Demiguisin E (**5**)

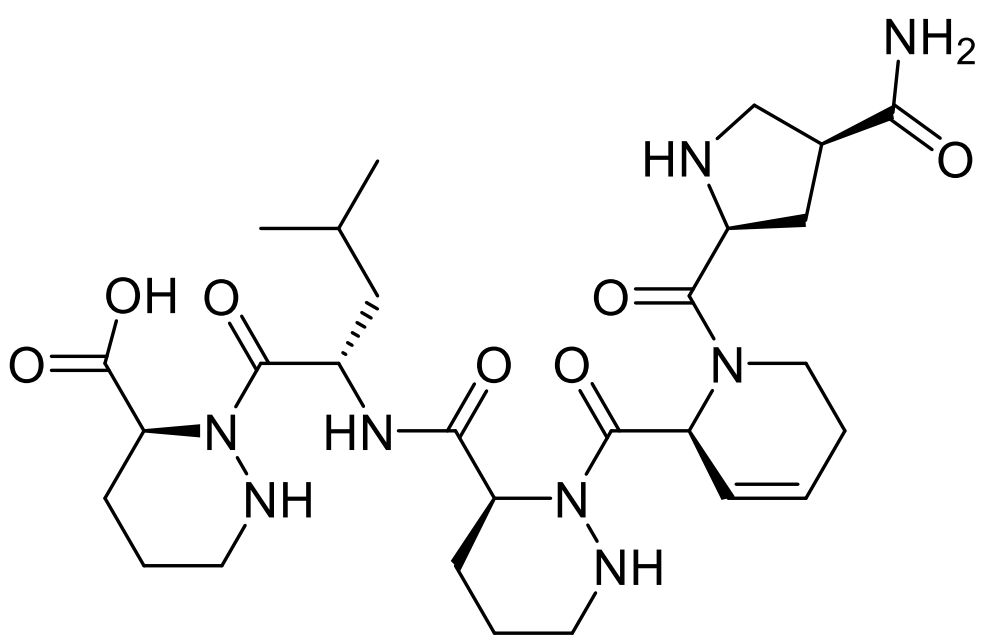

Demiguisin F (**6**)

Figure S6. Structures of the six demiguisin derivatives.

Table S7. ^1H and ^{13}C NMR data for demiguisins A (**1**) and B (**2**) in DMSO-d_6.

		Compound 1		Compound 2	
	No.	$\delta_C{}^a$	$\delta_H{}^b$	$\delta_C{}^{a,c}$	$\delta_H{}^b$
**Gly**	1	171.6		n.d	
	2	42.2	3.60, d (4.7)	43.5	3.64, m
					3.42, m
	2-NH		7.97, s		7.61, s
**β,γ-OH-Piz (1)**	3	168.8		170.8	
**γ-OH-Piz (2)**	4	56.2	4.77, s	48.0	4.77, d (6.5)
	5	68.1	4.12, s	33.6	2.39, m
					1.91, m
	6	65.0	3.36, m	60.4	3.64, m
	7	48.1	3.14, t (12.9)	52.8	2.80, m
			2.60, d (12.9)		2.72, m
**Leu**	8	174.3		n.d	
	9	47.3	5.30, m	47.1	5.21, m
	10	40.5	1.55, m	49.9	1.54, m
			1.33, t (9.5)		1.34, m
	11	24.7	1.56, m	24.3	1.62, m
	12	23.4	0.86, t (5.2)	23.1	0.86, m
	13	21.6	0.86, m	21.3	0.86, m
	9-NH		7.97, d (9.0)		8.01, d (8.8)
**Piz**	14	170.7		170.9	
	15	49.7	4.95, dd (17.4, 10.0)	49.6	4.91, d (5.8)
	16	26.6	2.07, m	26.2	2.08, m
			1.76, m		1.76, m
	17	20.9	1.45, m	20.6	1.46, m
	18	46.6	2.99, t (9.6)	46.3	2.98, m
			2.70, m		2.72, m
	18-NH		5.11, d (12.9)		5.11, d (15.0)
**DHPA**	19	170.0		170.0	
	20	52.0	5.75, s	51.6	5.73, s
	21	122.6	5.89, m	122.6	5.88, m
	22	126.6	5.93, m	126.6	5.91, m
	23	24.6	2.20, m	24.6	2.19, m
			2.06, m		2.02, m
	24	40.1	3.93, m	39.6	3.94, m
			3.36, m		3.35, m
**γ-c-Pro**	25	170.9		171.9	
	26	58.6	4.12, m	58.6	3.88, m
	27	33.6	2.40, dt (14.2, 7.8)	33.9	2.27, m
			1.68 (12.4, 9.6)		1.64, m
	28	44.3	2.88, m	44.6	2.80, m
	29	50.0	3.07, m	50.3	2.99, m
			2.99, t (9.6)		2.80, m
	30	174.6		175.6	
	30-NH_2		7.42, s		7.35, s
			6.93, s		6.82, s

Measured in DMSO-d_6, [a]125 MHz, [b]500 MHz, [c]^{13}C NMR data were assigned based on HSQC and HMBC experiments.

Table S8. ^1H and ^{13}C NMR data for demiguisins C-F (**3**-**6**) in DMSO-d_6.

		Compound 3		Compound 4		Compound 5		Compound 6	
	**No.**	δ_C[a,b]	δ_H[c]	δ_C[c,d]	δ_H[e]	δ_C[a,c]	δ_H[b]	δ_C[a,c]	δ_H[b]
**β,γ-OH-Piz (3)**	1	n.d.[f]		173.1		n.d.		n.d.	
**γ-OH-Piz (4)**	2	55.6	4.70, s	47.6	4.76, d (7.2)	60.6	4.46, m	53.3	4.51, m
**β-OH-Piz (5)**	3	67.5	4.09, s	32.2	2.21, m	64.4	4.21, m	26.7	2.09, m
**OH-Piz (6)**					1.90, m				1.52, m
	4	64.6	3.40, m	60.2	3.69, m	29.2	1.59, m	22.9	1.75, m
							1.37, m		
	5	48.0	3.15, m	52.5	2.87, m	41.9	3.04, m	48.1	2.90, m
			2.61, m		2.81, m				2.87, m
**Leu**	6	n.d.		173.5		n.d.		n.d.	
	7	47.3	5.32, m	47.2	5.21, m	47.2	5.29, m	47.2	5.22, m
	8	40.1	1.57, m	40.1	1.50, m	41.1	1.52, m	41.3	1.47, m
			1.34, m		1.37, m		1.23, m		1.28, m
	9	24.6	1.57, m	24.4	1.61, m	24.4	1.56, m	24.6	1.61, m
	10	23.3	0.87, m	23.3	0.87, m	23.3	0.85, m	23.6	0.85, t (6.9)
	11	21.6	0.87, m	21.4	0.87, m	22.1	0.85, m	22.1	0.85, t (6.9)
	7-NH		7.98, d (8.7)		8.03, d (8.5)		7.82, d (9.1)		7.86, d (8.7)
**Piz**	12	n.d.		171.1		n.d.		n.d.	
	13	49.9	4.94, m	49.9	4.93, m	50.1	4.93, m	50.2	4.93, m
	14	26.3	2.08, m	26.7	2.09, m	26.5	2.09, m	26.8	1.83, m
			1.75, m		1.76, m		1.76, m		
	15	20.8	1.47, m	20.8	1.47, m	20.8	1.47, m	21.9	1.47, m
	16	46.4	2.99, m	46.4	3.01, t (11.5)	46.3	2.95, m	46.9	3.00, m
			2.70, m		2.72, m		2.71, m		2.73, m
	16-NH		5.14, d (13.6)		5.17, d (13.4)		5.08, d (12.2)		5.12, d (12.1)
**DHPA**	17	n.d.		n.d.		n.d.		n.d.	
	18	52.0	5.76, s	52.1	5.76, s	51.6	5.75, s	52.0	5.75, s
	19	121.6	5.90, m	121.6	5.91, m	122.6	5.87, m	122.8	5.88, m
	20	126.6	5.96, m	126.4	5.97, m	126.5	5.92, m	127.0	5.91, m
	21	24.2	2.21, m	24.0	2.21, m	24.1	2.19, m	24.6	2.18, m
			2.08, m		2.11, m		2.04, m		2.04, m
	22	39.5	3.92, m	40.3	3.90, m	39.5	3.95, m	n.d.	3.96, m
			3.36, m		3.38, m		3.39, m		3.22, m
**γ-c-Pro**	23	n.d.		n.d.		n.d.		n.d.	
	24	57.9	4.76, m	58.2	4.77, m	58.8	3.87, m	58.6	3.87, m
	25	31.8	2.67, m	31.7	2.71, m	29.8	2.28, m	34.1	2.27, m
			1.77, m		1.78, m		1.64, m		1.66, m
	26	42.4	3.04, m	42.2	3.10, m	44.6	2.80, m	45.1	2.80, m
	27	48.4	3.15, m	47.7	3.38, m	50.0	3.00, m	50.6	3.00, m
			3.04, m		3.28, m		2.80, m		2.80, m
	28	n.d.		n.d.		n.d.		n.d.	
	28-NH_2		7.57, s		7.63, s		7.32, s		7.33, s
			7.09, s		7.20, s		6.79, s		6.78, s

Measured in DMSO-d_6, [a]125 MHz, [b]^{13}C NMR data were assigned based on HSQC and HMBC experiments, [c]500 MHz
[d]175 MHz, [e]700 MHz, [f]not detected

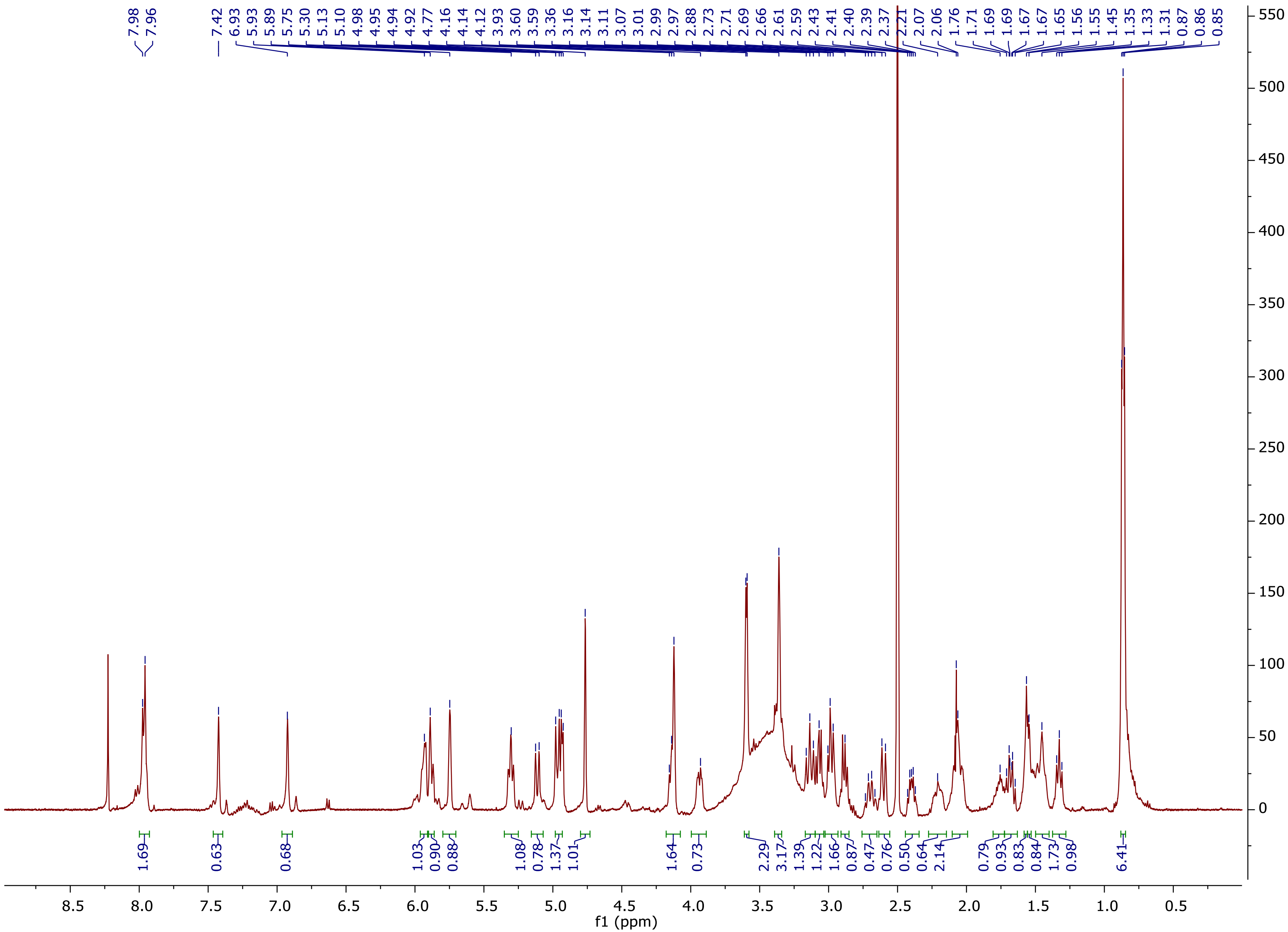

Figure S7. ^{1}H NMR spectrum of compound **1** (500 MHz, DMSO-d_6).

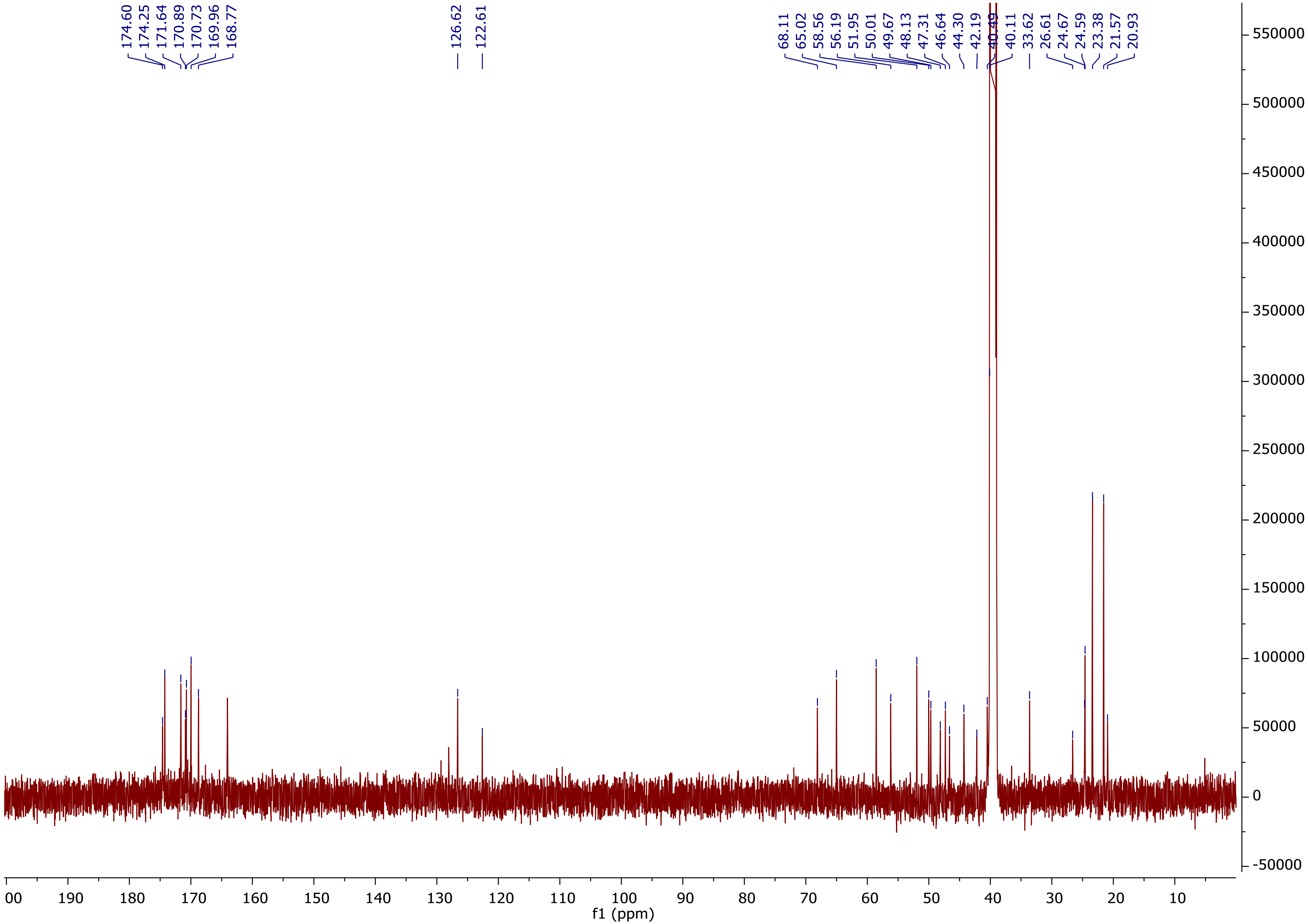

Figure S8. ^{13}C NMR spectrum of compound **1** (125 MHz, DMSO-d_6).

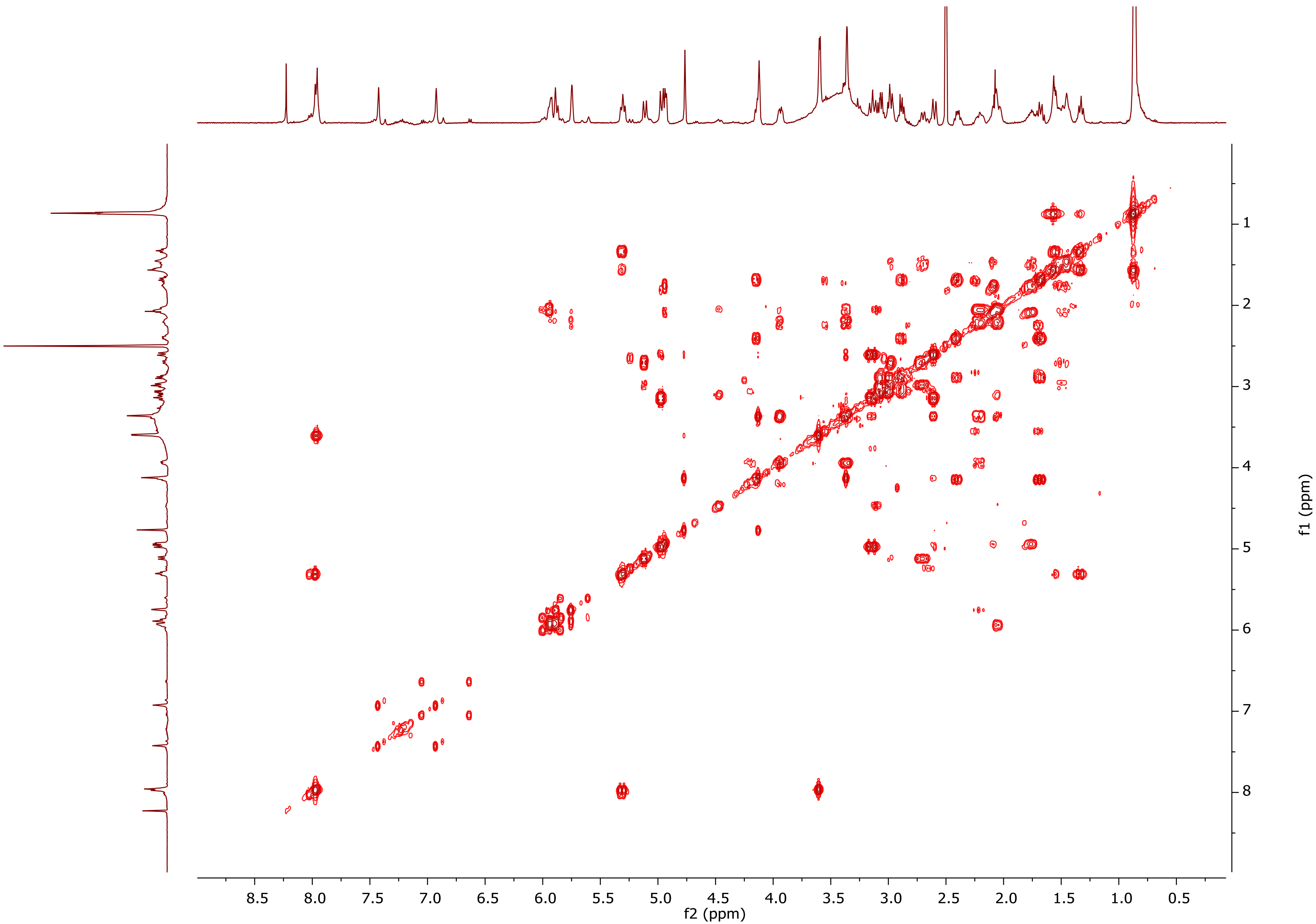

Figure S9. ^1H-^1H COSY NMR spectrum of compound **1** (500/500 MHz, DMSO-d_6).

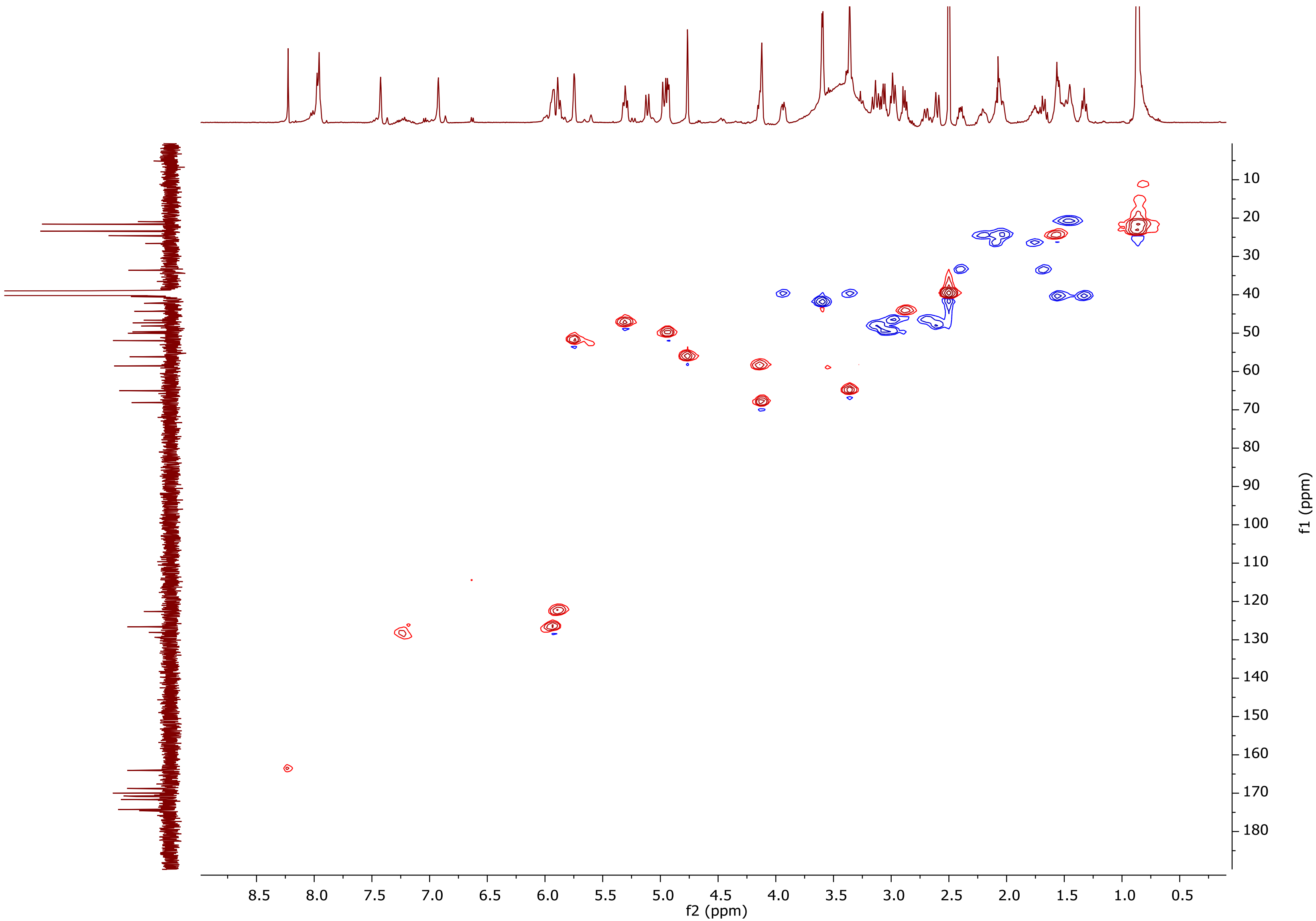

Figure S10. ^{1}H-^{13}C HSQC NMR spectrum of compound **1** (500/125 MHz, DMSO-d_6).

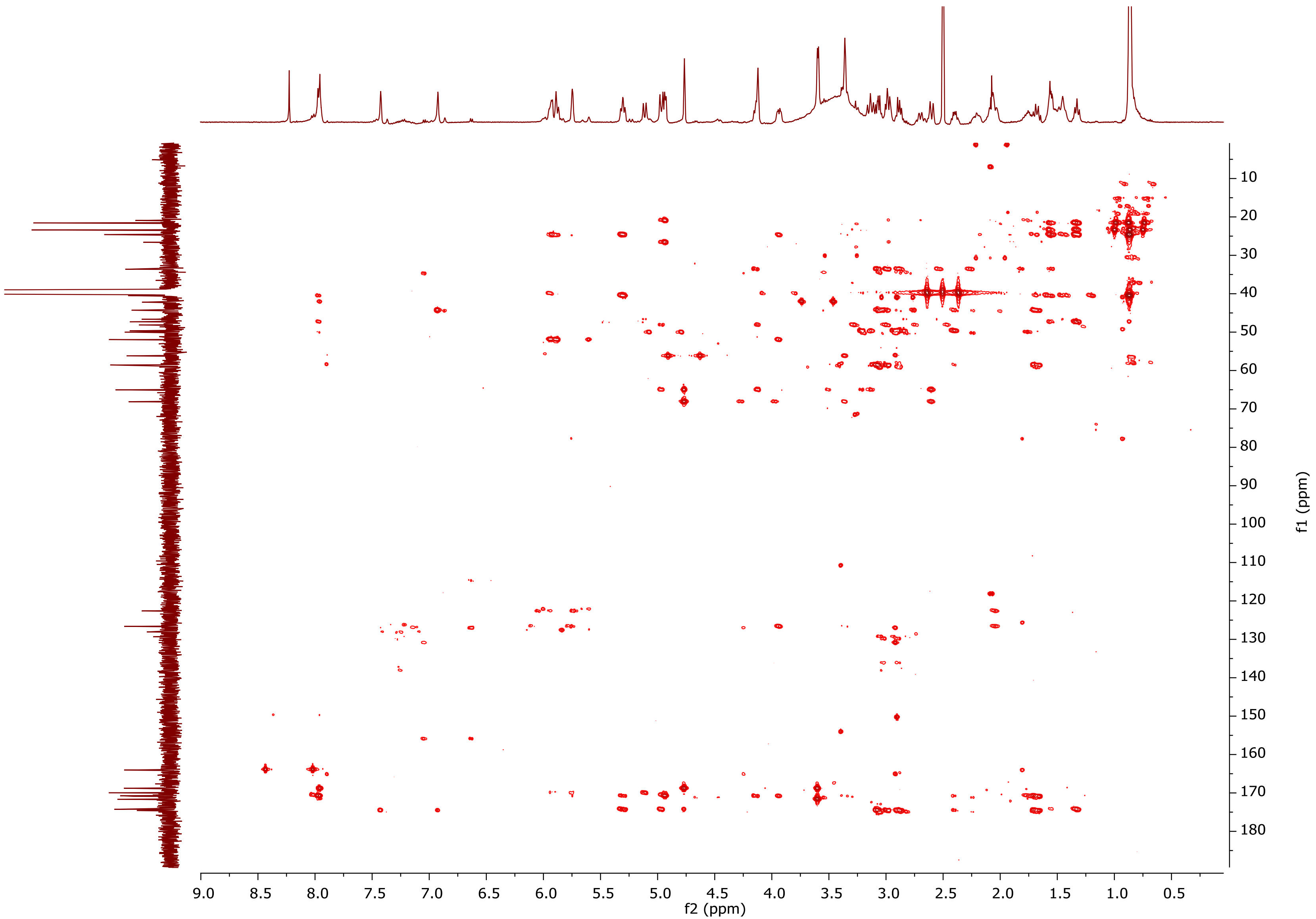

Figure S11. ^{1}H-^{13}C HMBC NMR spectrum of compound **1** (500/125 MHz, DMSO-d_6).

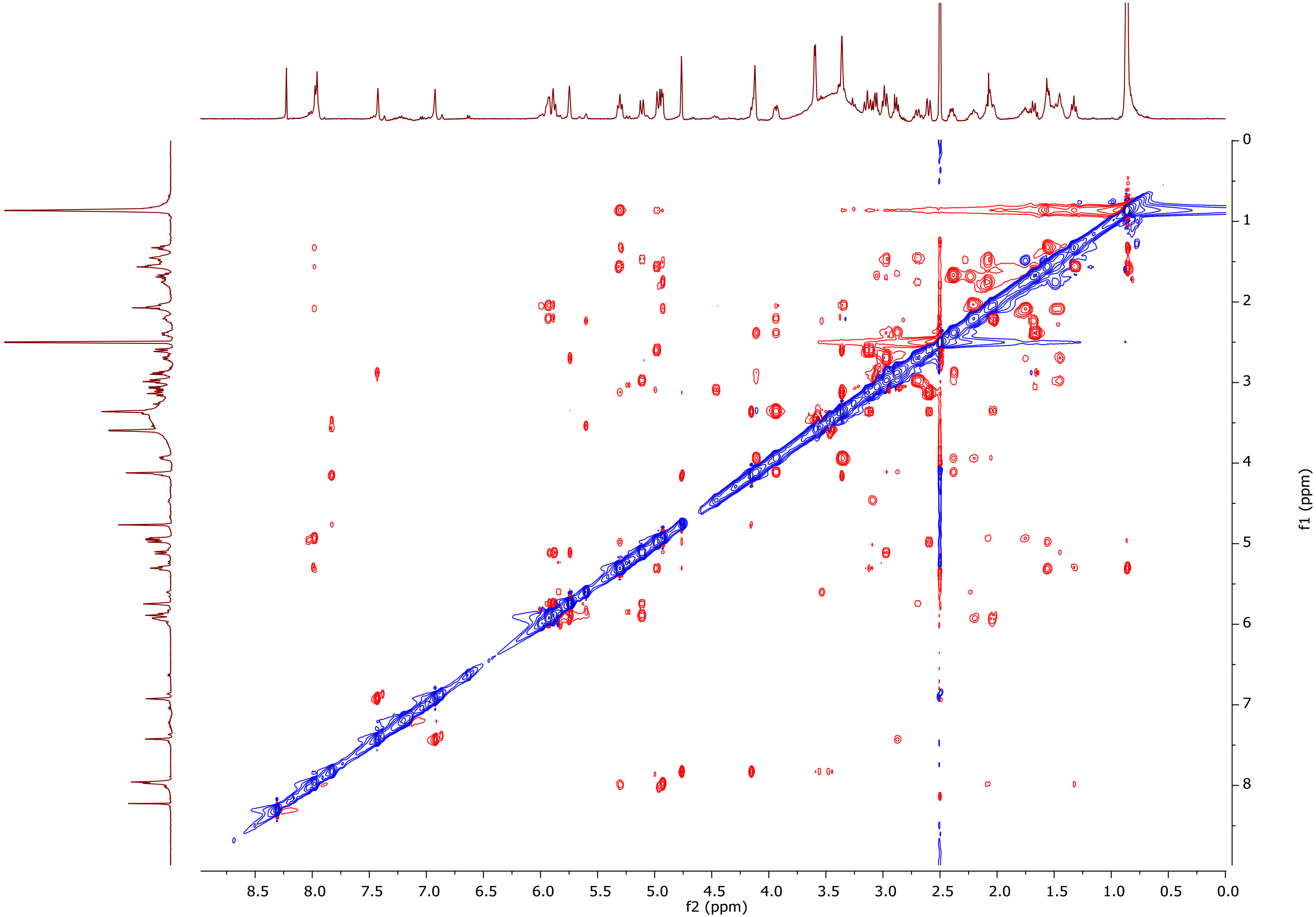

Figure S12. ^{1}H-^{1}H ROESY NMR spectrum of compound **1** (500/500 MHz, DMSO-d_6).

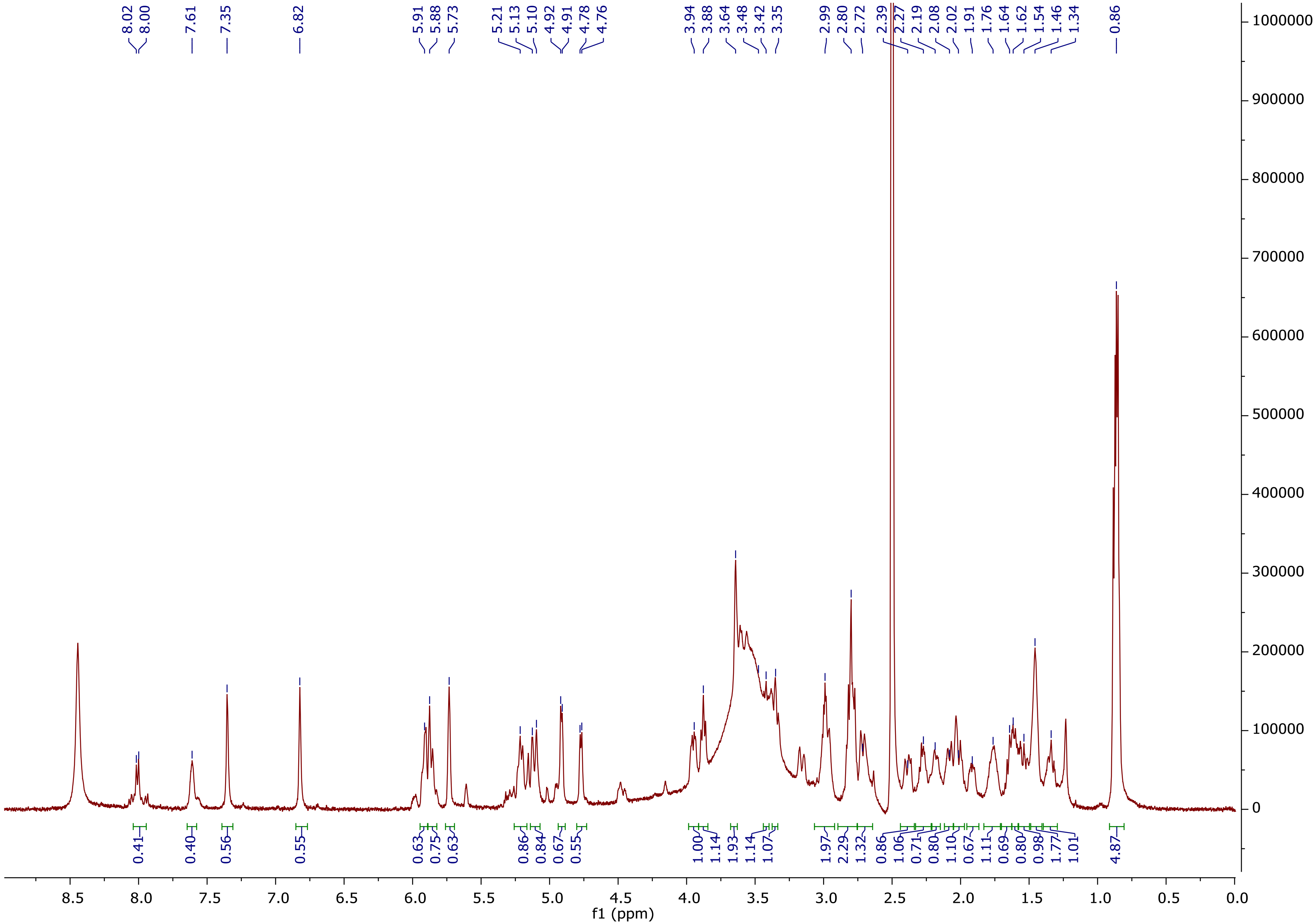

Figure S13. ^{1}H NMR spectrum of compound **2** (500 MHz, DMSO-d_6).

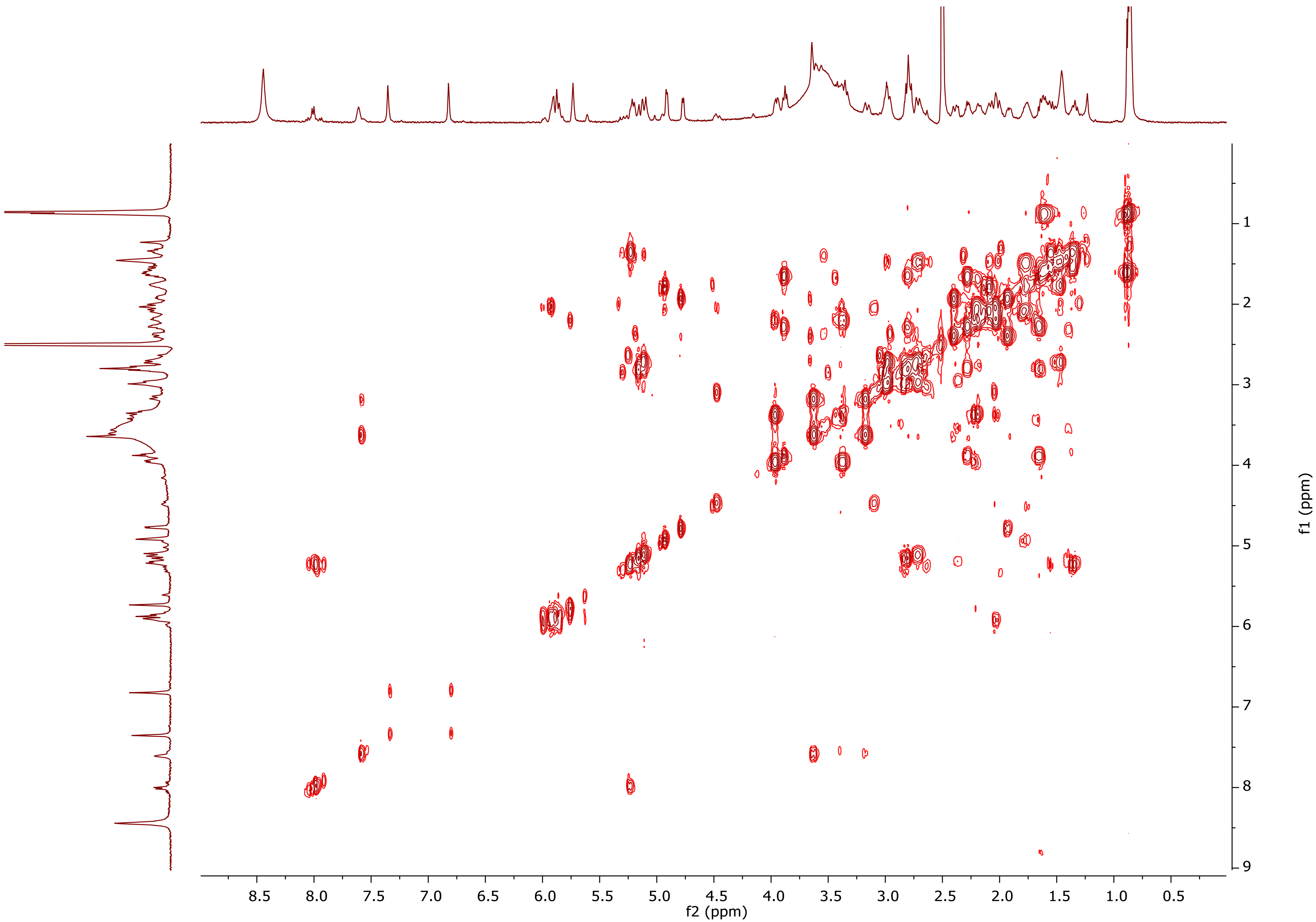

Figure S14. ^{1}H-^{1}H COSY NMR spectrum of compound **2** (500/500 MHz, DMSO-d_6).

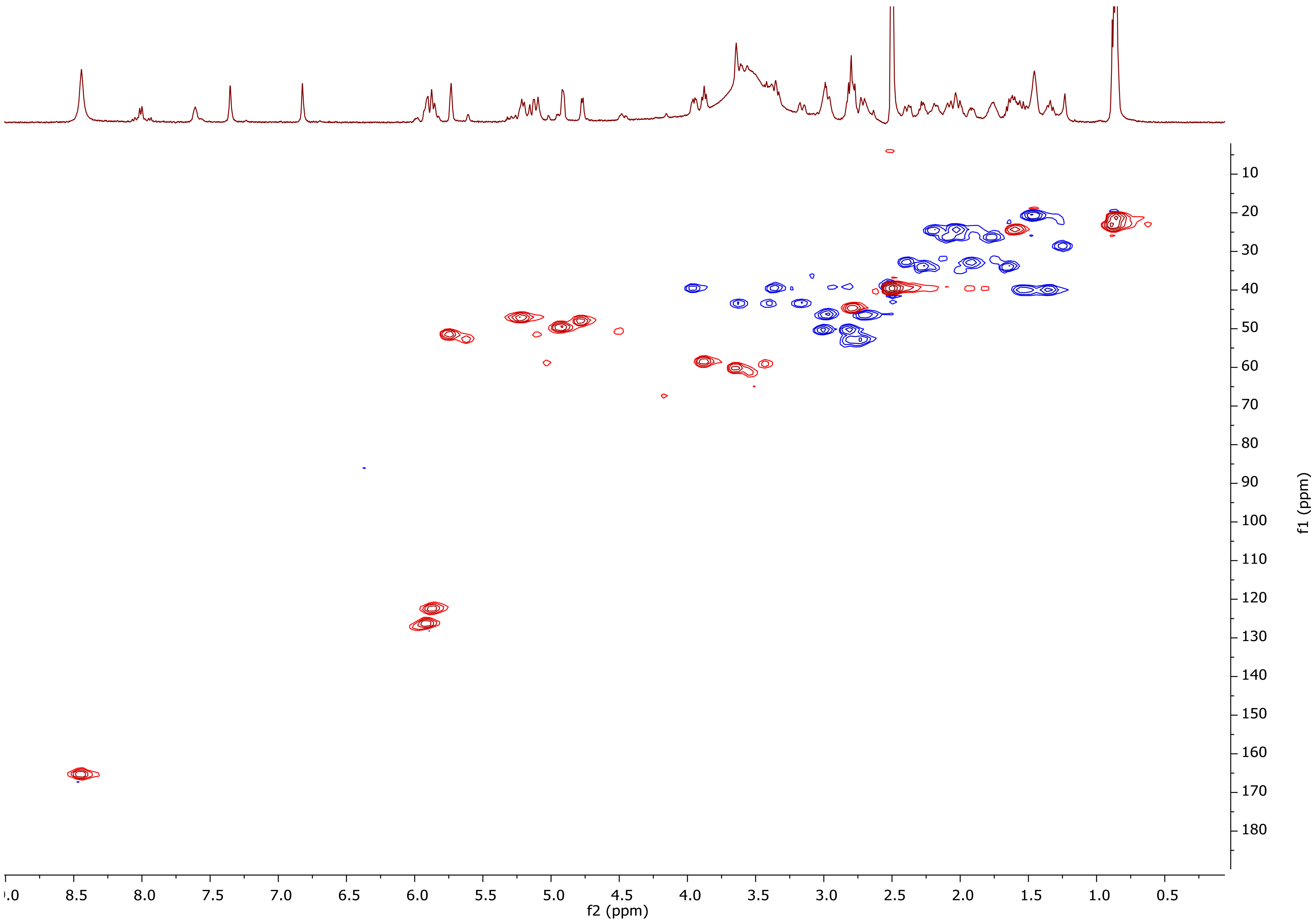

Figure S15. ^{1}H-^{13}C HSQC NMR spectrum of compound **2** (500/125 MHz, DMSO-d_6).

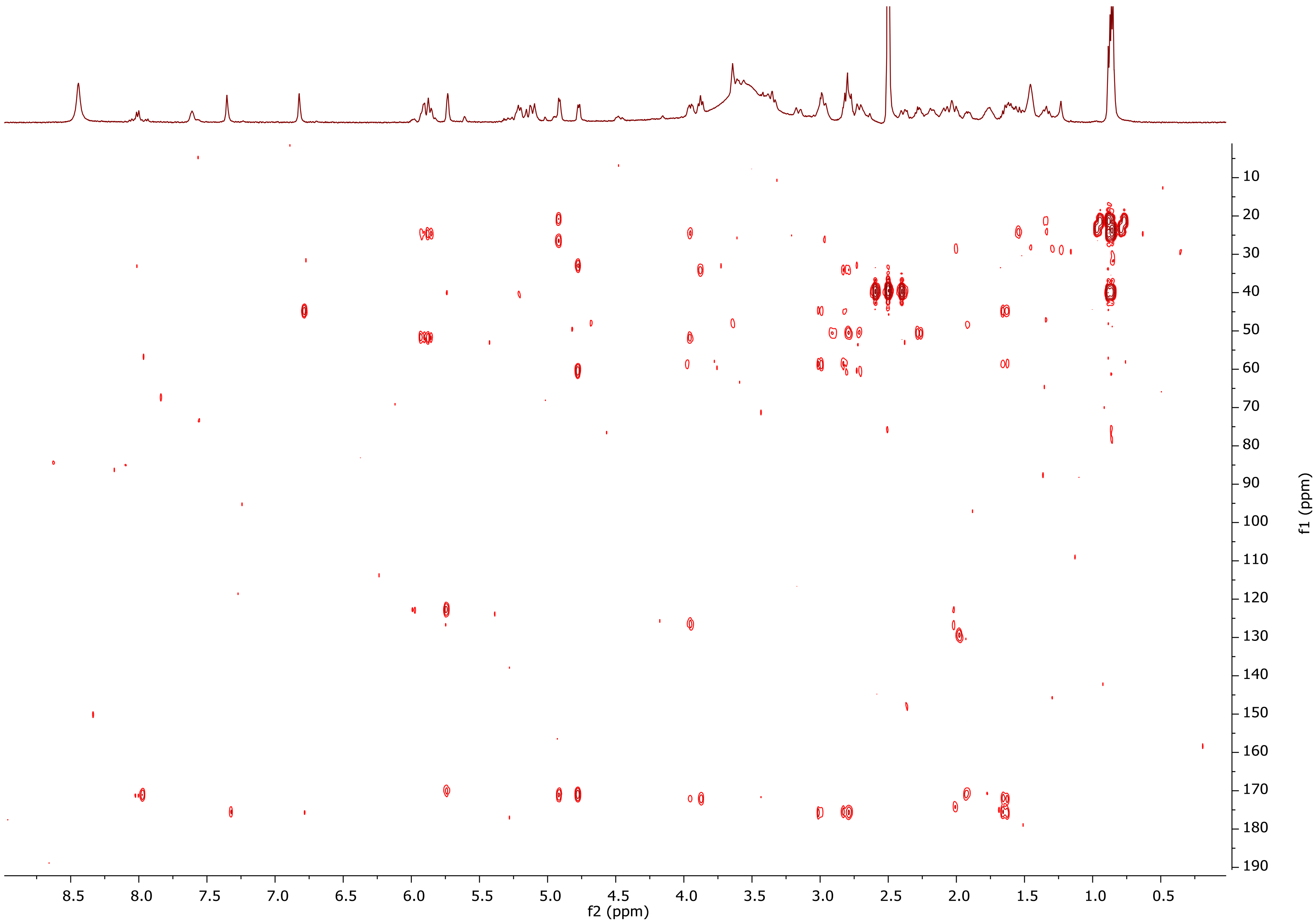

Figure S16. ^{1}H-^{13}C HMBC NMR spectrum of compound **2** (500/125 MHz, DMSO-d_6).

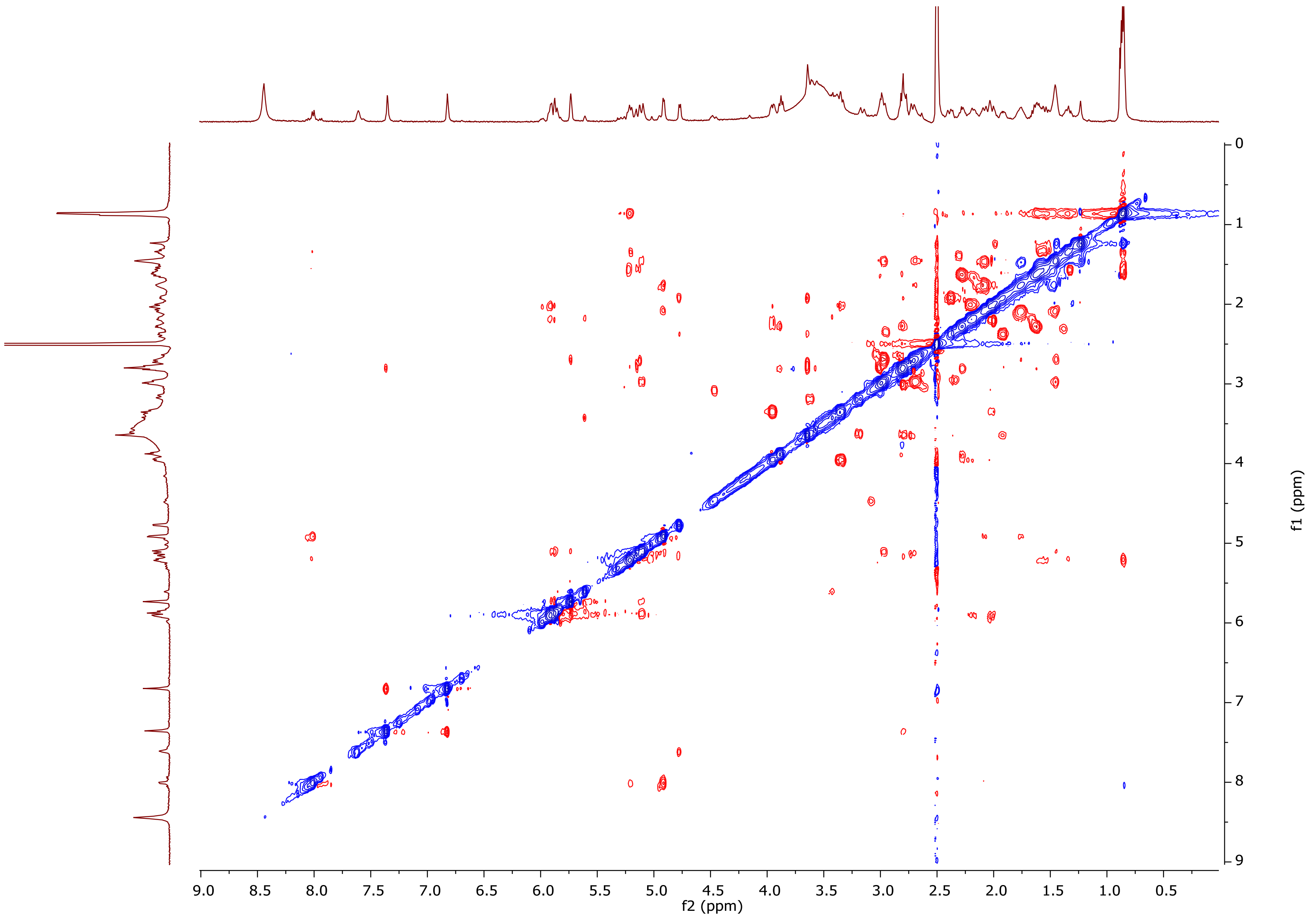

Figure S17. ^{1}H-^{1}H NOESY NMR spectrum of compound **2** (500/500 MHz, DMSO-d_6).

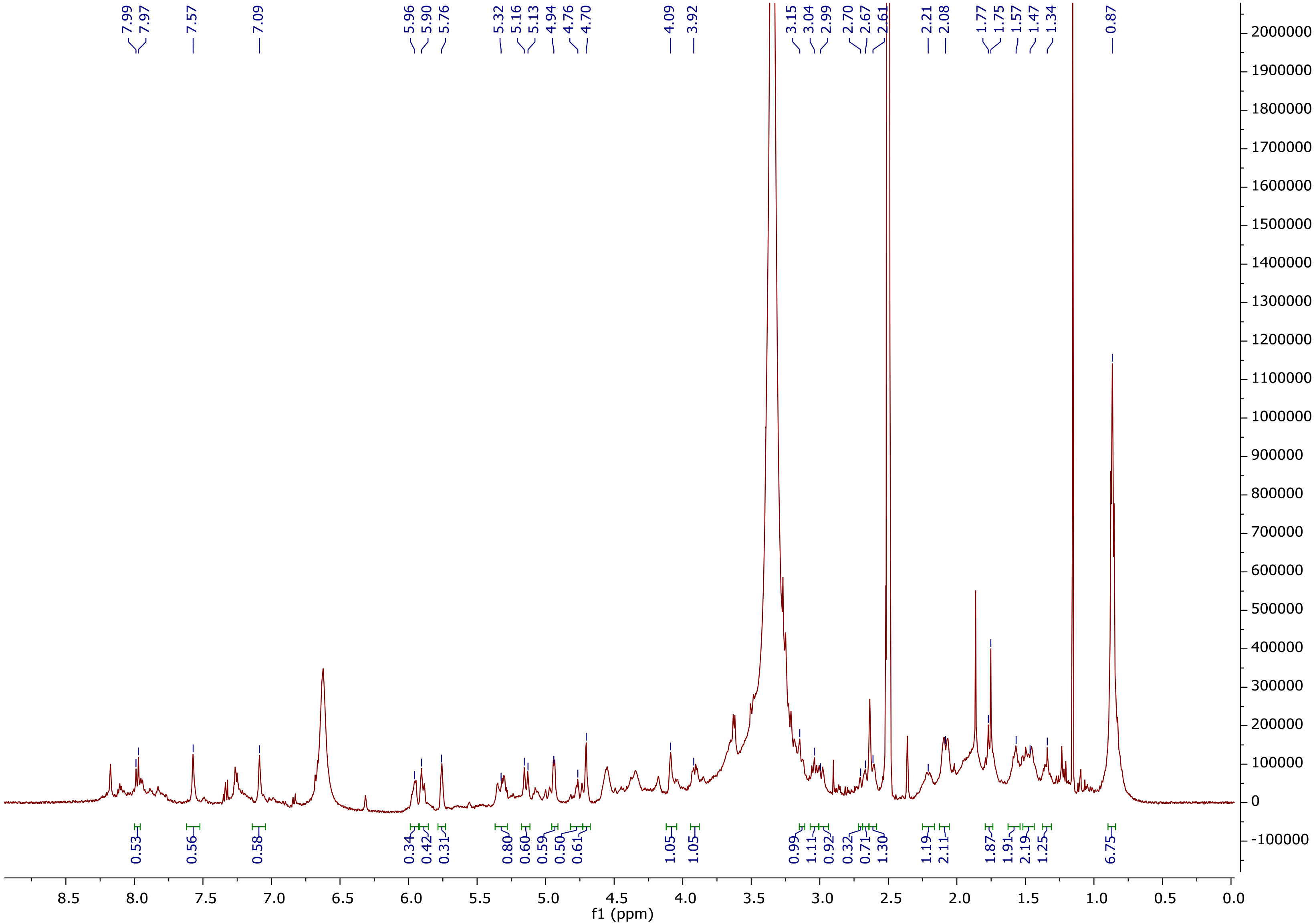

Figure S18. ^{1}H NMR spectrum of compound **3** (500 MHz, DMSO-d_6).

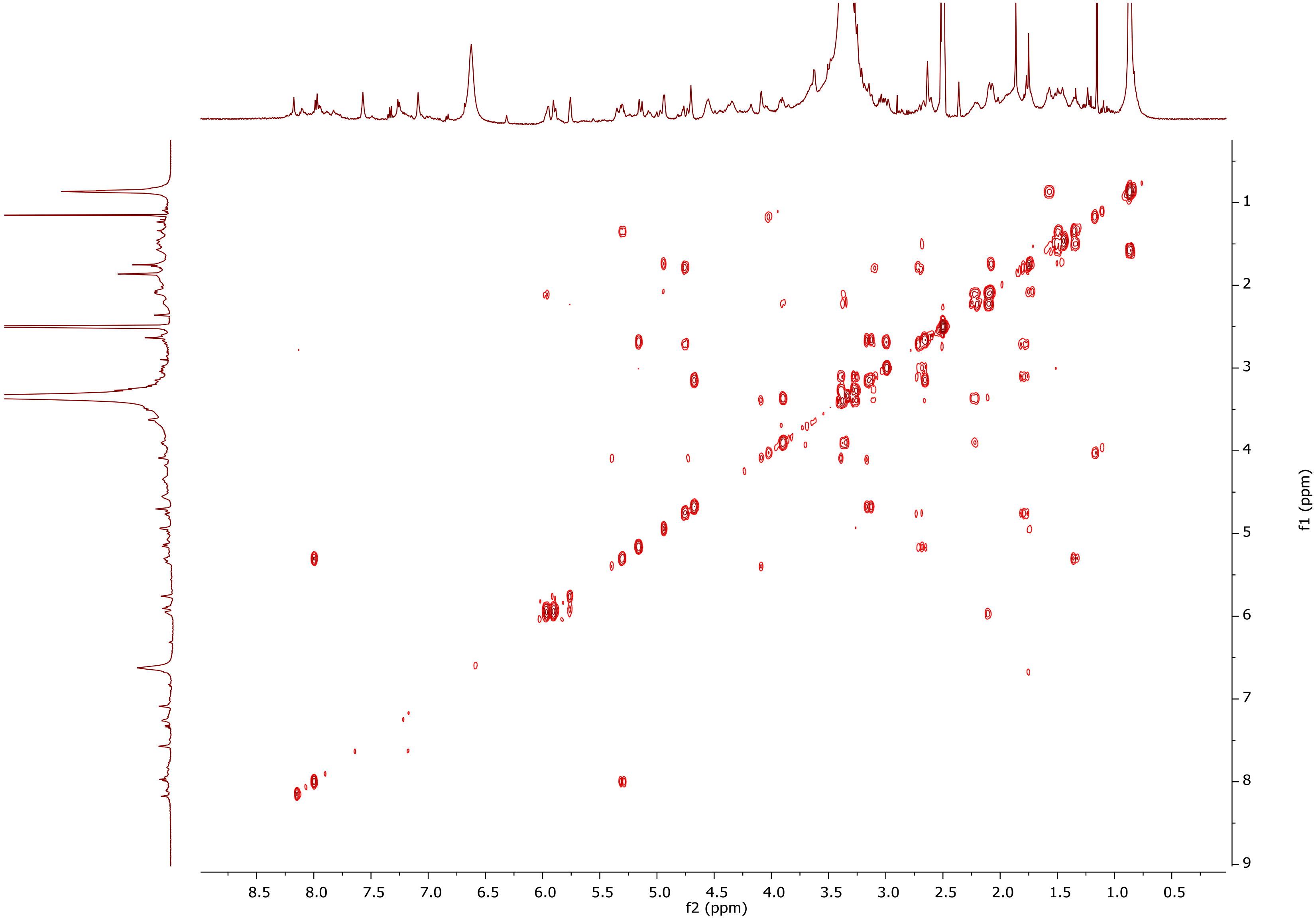

Figure S19. ^{1}H-^{1}H COSY NMR spectrum of compound **3** (500/500 MHz, DMSO-d_6).

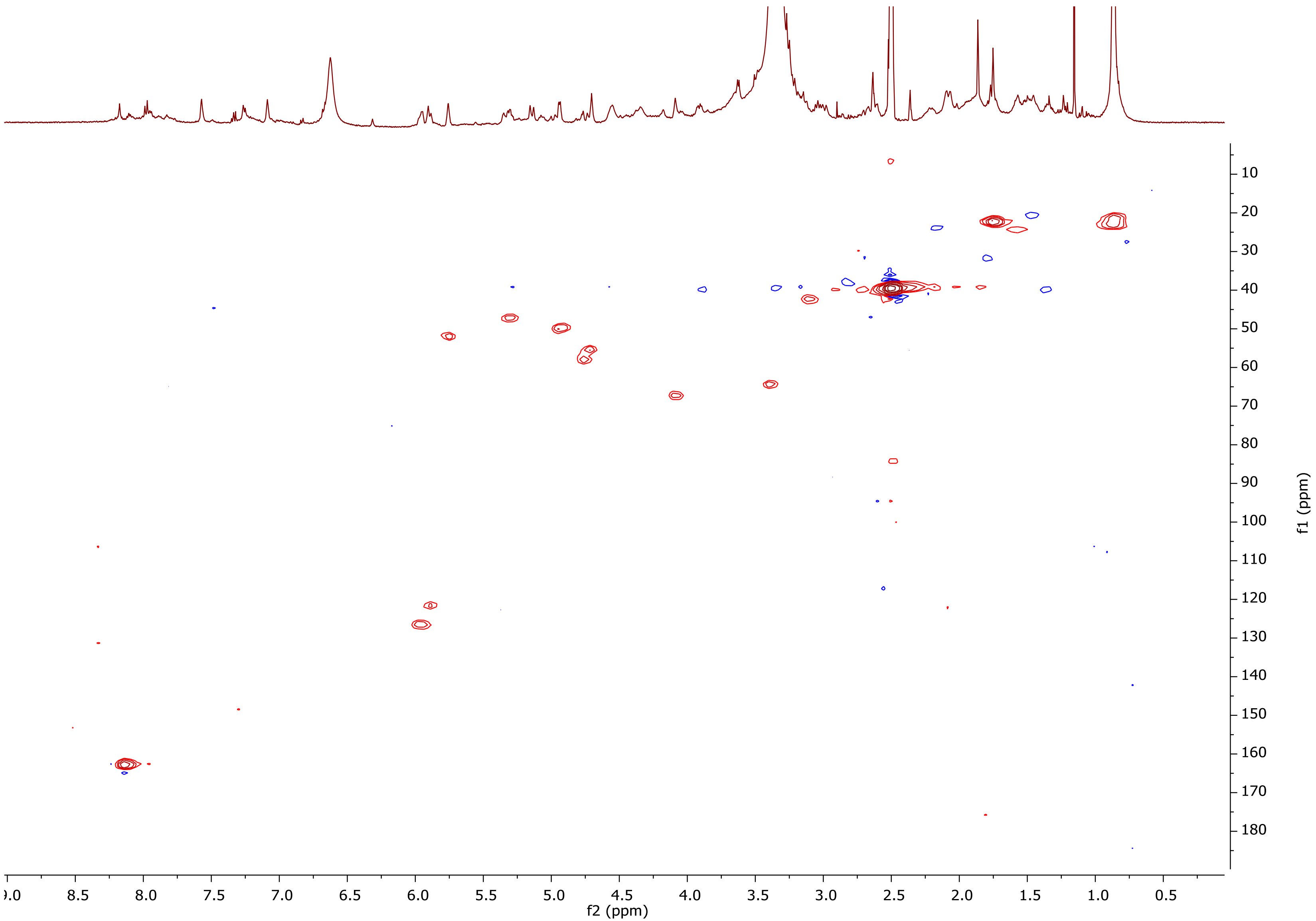

Figure S20. ^{1}H-^{13}C HSQC NMR spectrum of compound **3** (500/125 MHz, DMSO-d_6).

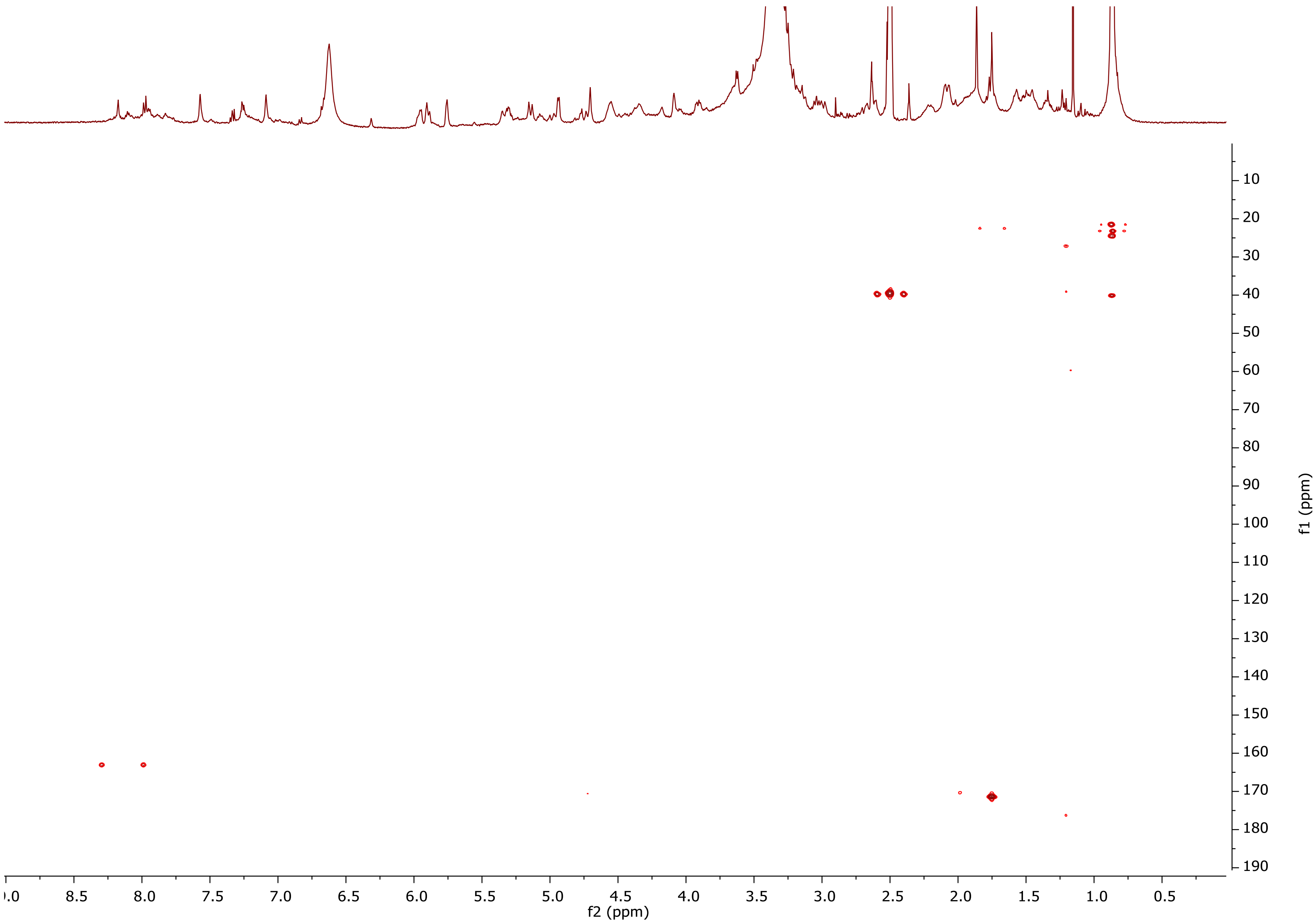

Figure S21. ^{1}H-^{13}C HMBC NMR spectrum of compound **3** (500/125 MHz, DMSO-d_6).

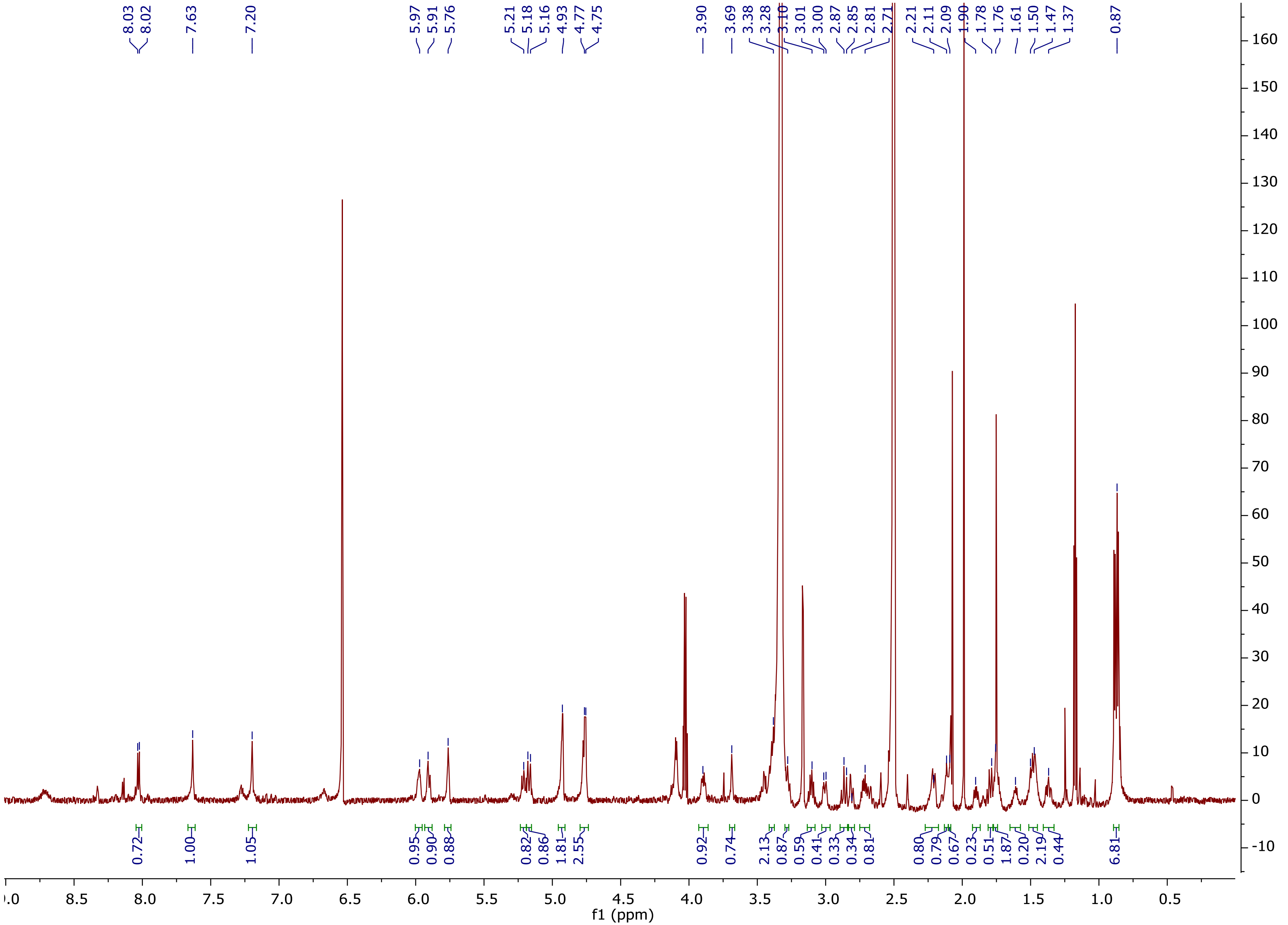

Figure S22. ^{1}H NMR spectrum of compound **4** (700 MHz, DMSO-d_6).

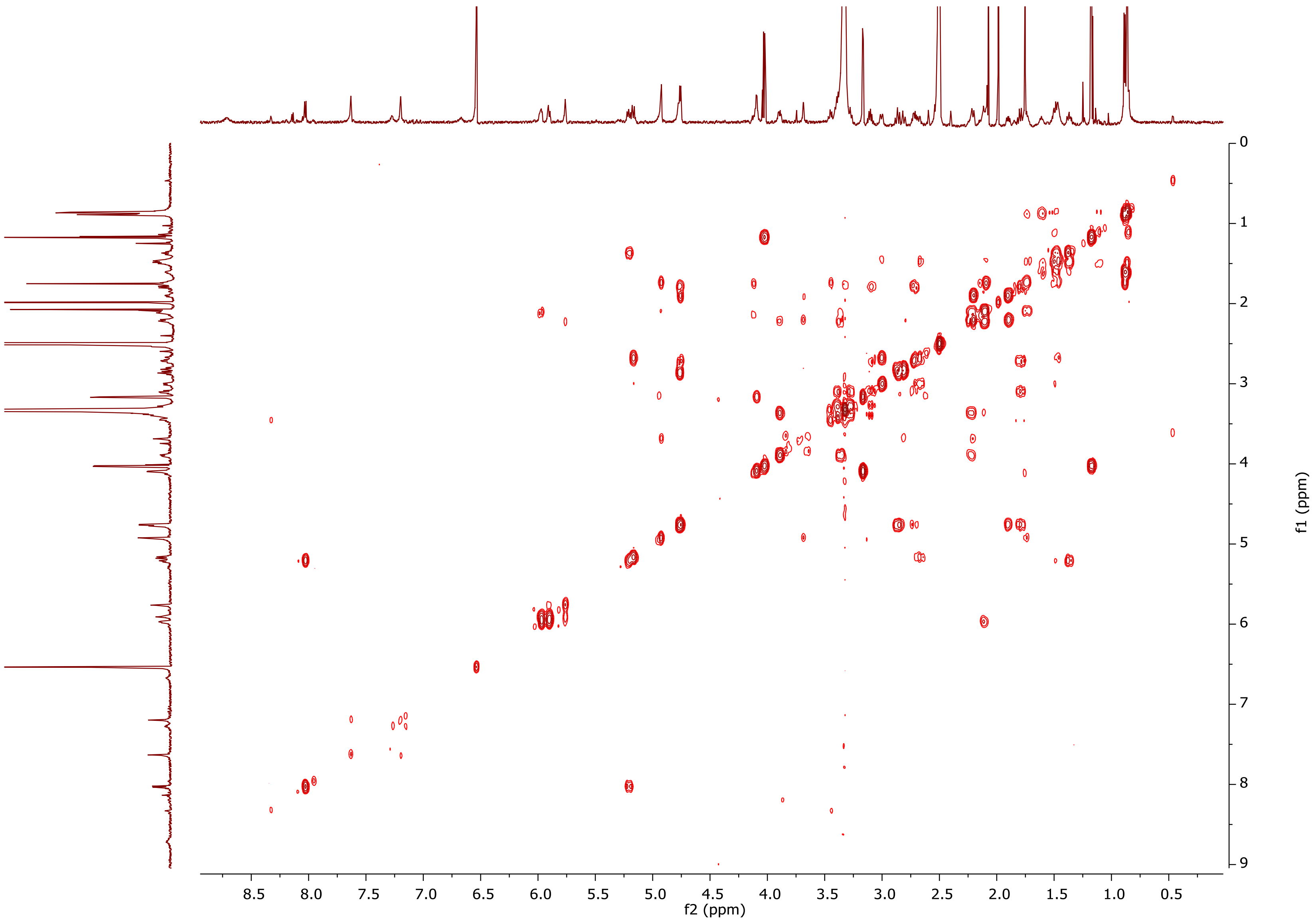

Figure S23. ^{1}H-^{1}H COSY NMR spectrum of compound **4** (700/700 MHz, DMSO-d_6).

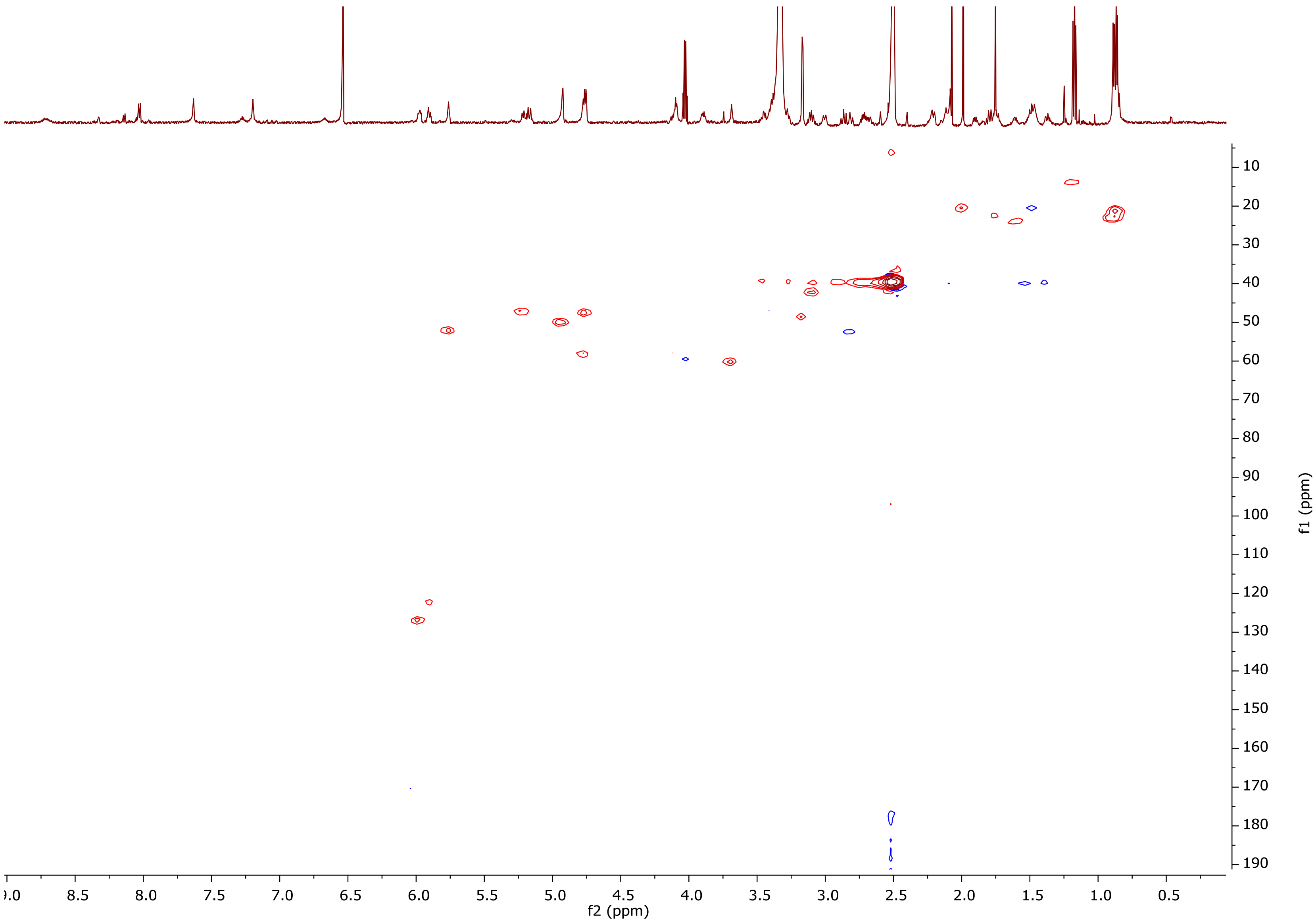

Figure S24. ^{1}H-^{13}C HSQC NMR spectrum of compound **4** (700/175 MHz, DMSO-d_6).

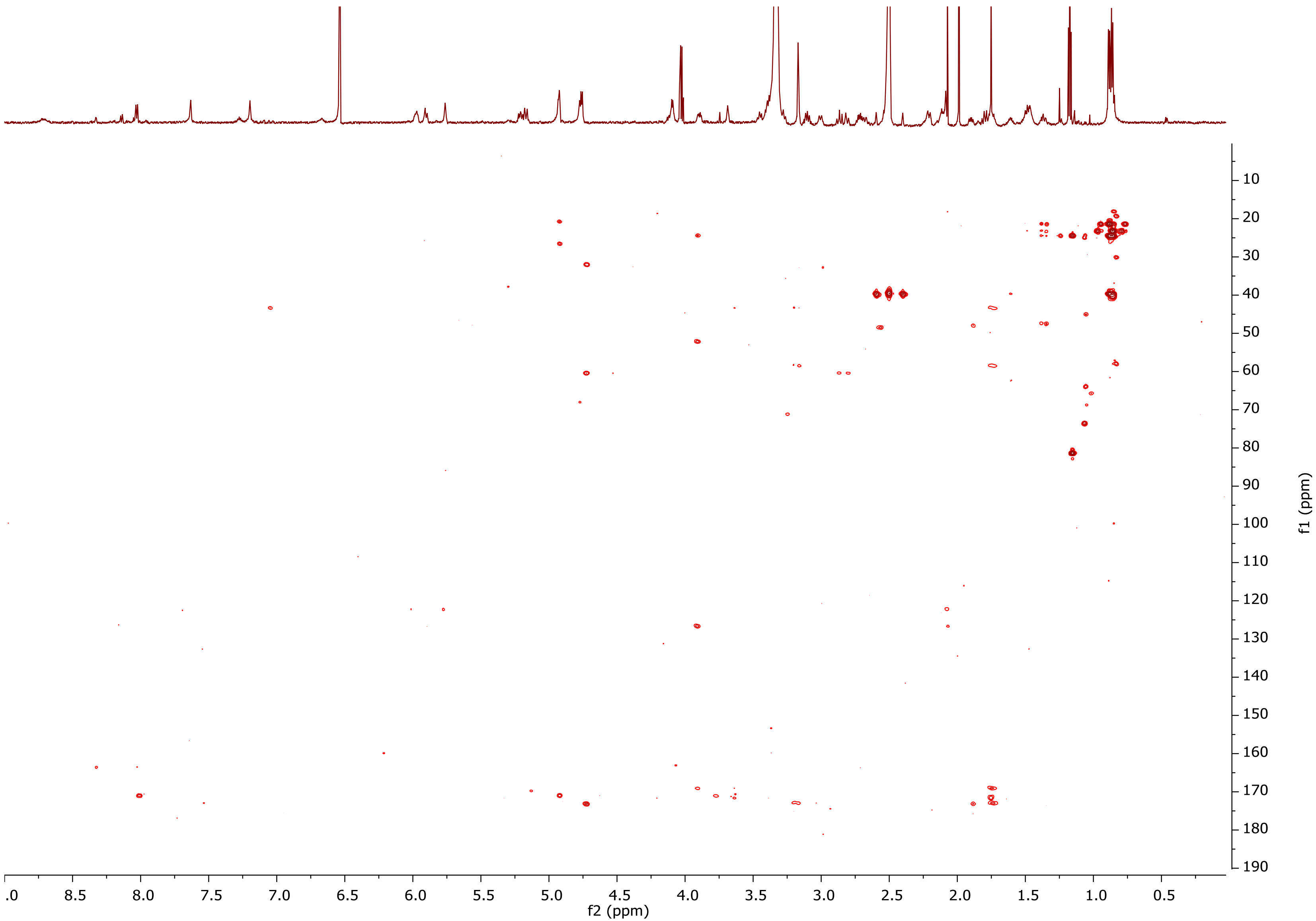

Figure S25. ^{1}H-^{13}C HMBC NMR spectrum of compound **4** (700/175 MHz, DMSO-d_6).

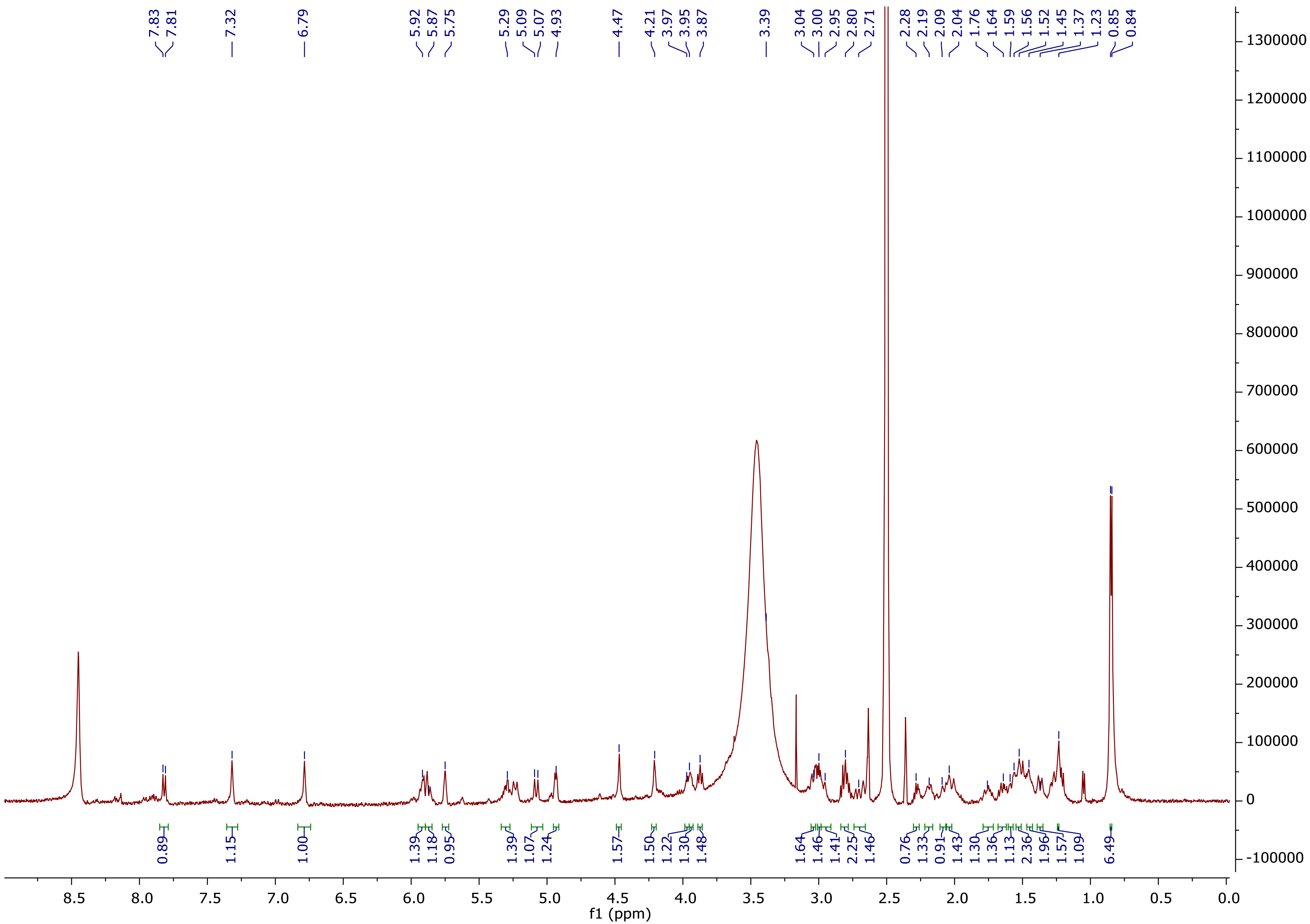

Figure S26. ^{1}H NMR spectrum of compound **5** (500 MHz, DMSO-d_6).

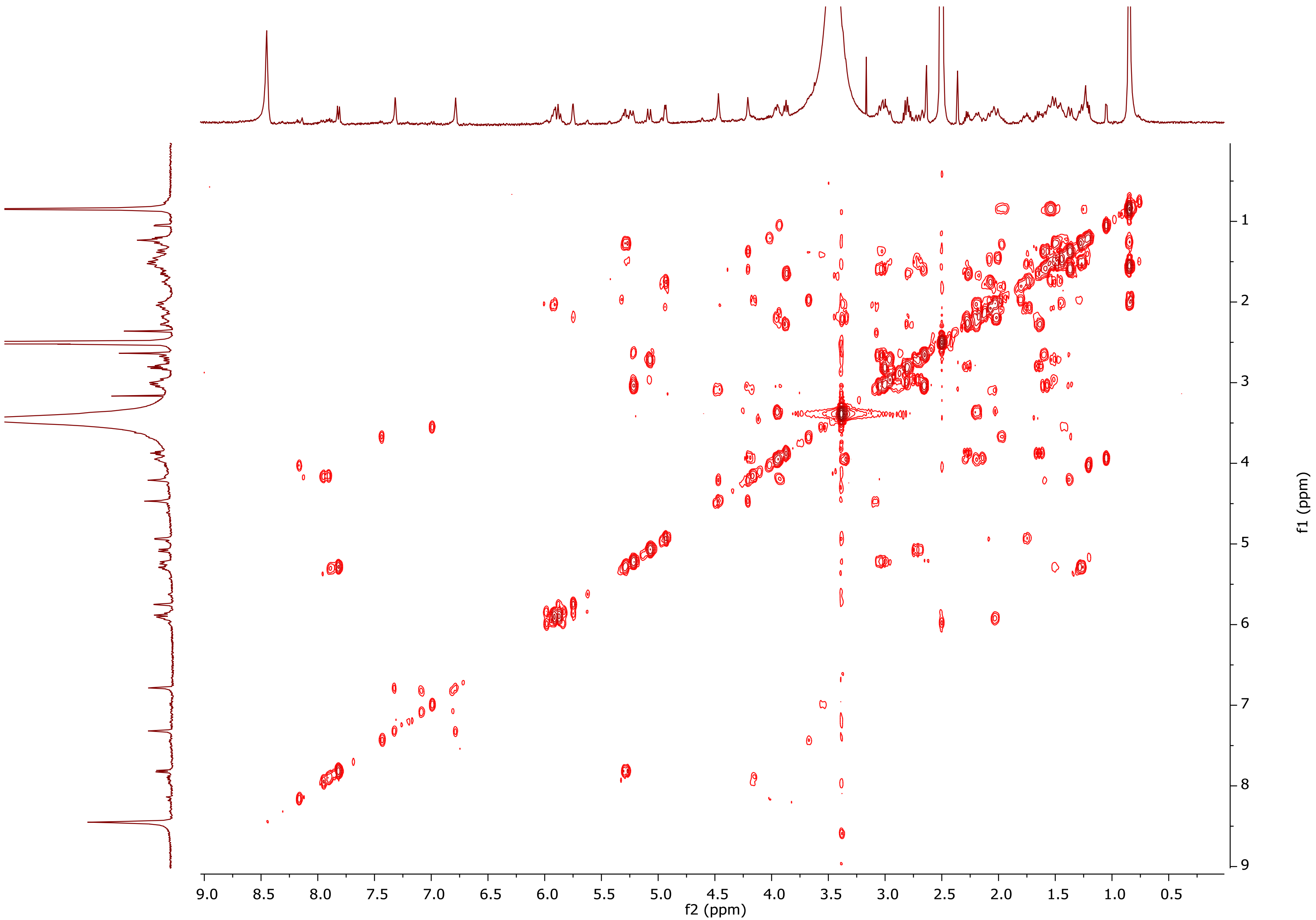

Figure S27. ^{1}H-^{1}H COSY NMR spectrum of compound **5** (500/500 MHz, DMSO-d_6).

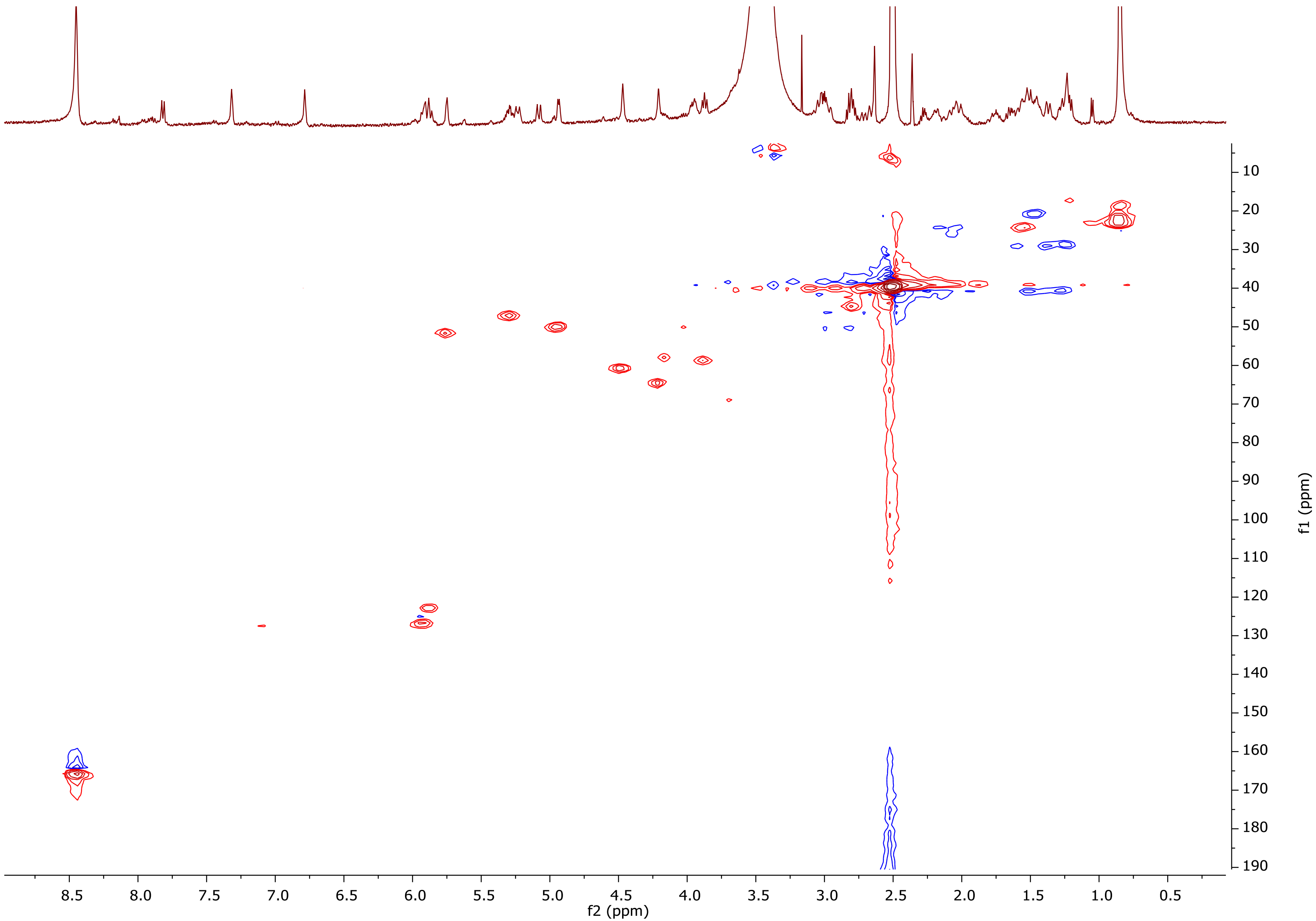

Figure S28. ^{1}H-^{13}C HSQC NMR spectrum of compound **5** (500/125 MHz, DMSO-*d*$_6$).

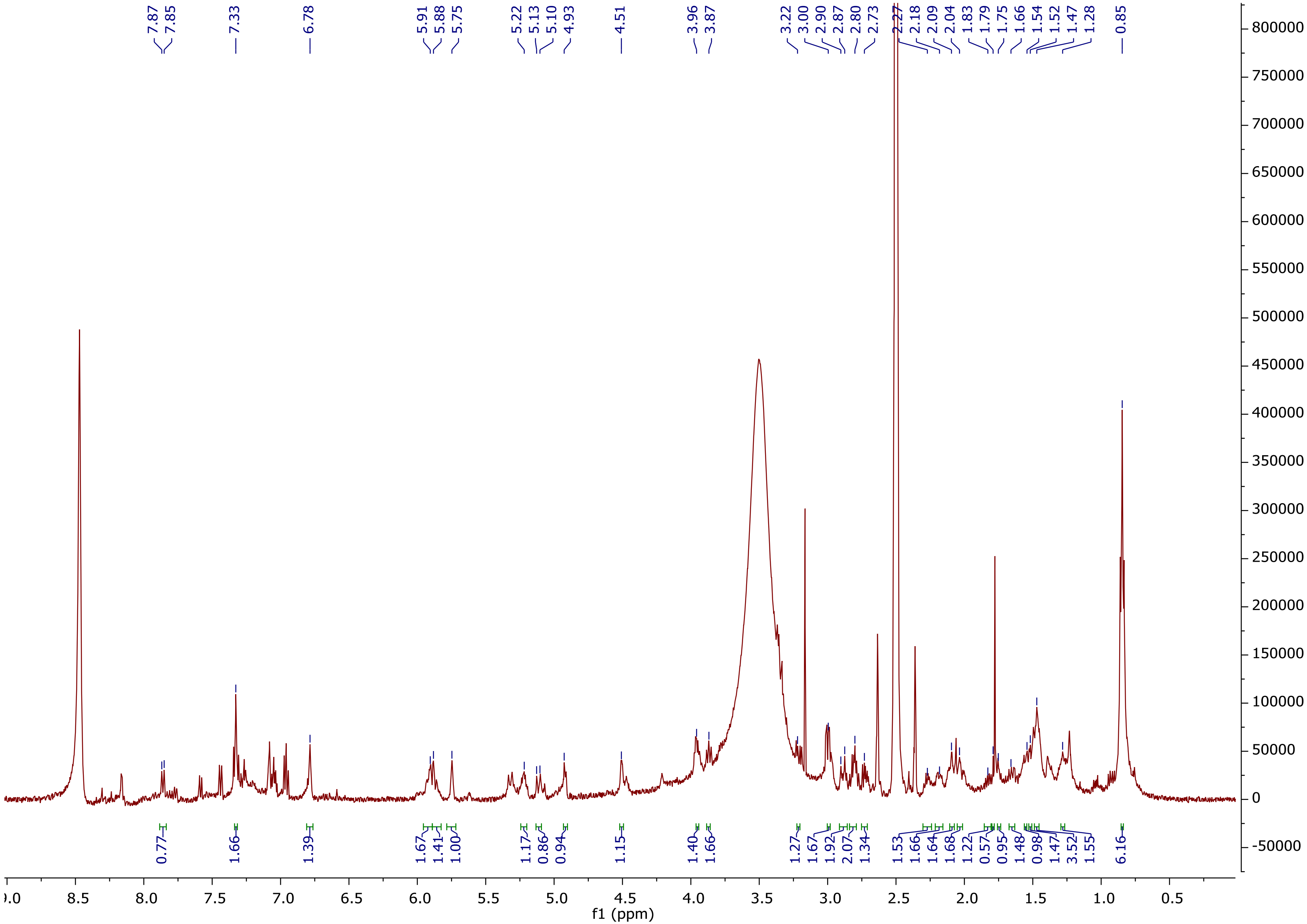

Figure S29. ^{1}H NMR spectrum of compound **6** (500 MHz, DMSO-d_6).

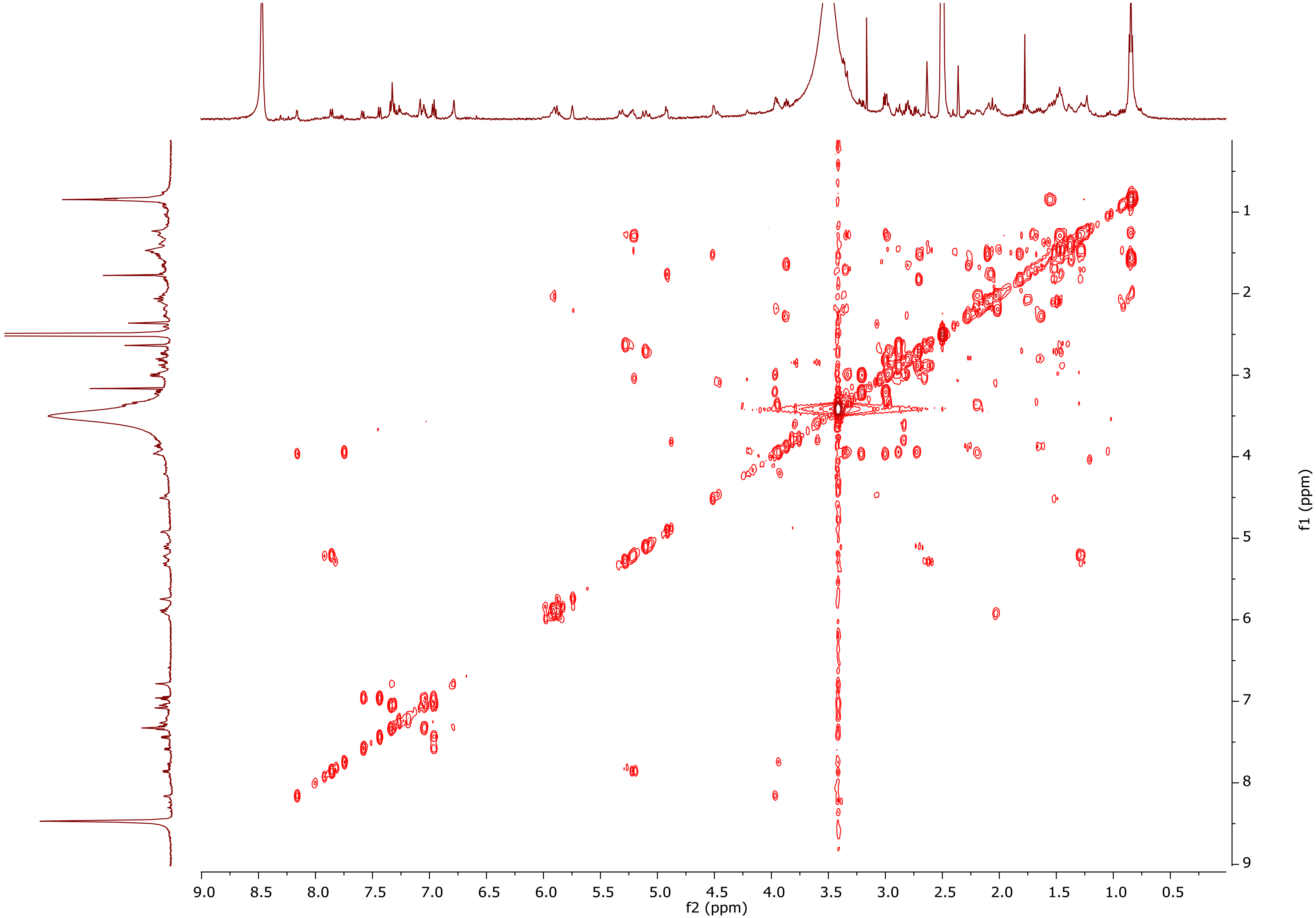

Figure S30. ^{1}H-^{1}H COSY NMR spectrum of compound **6** (500/500 MHz, DMSO-d_6).

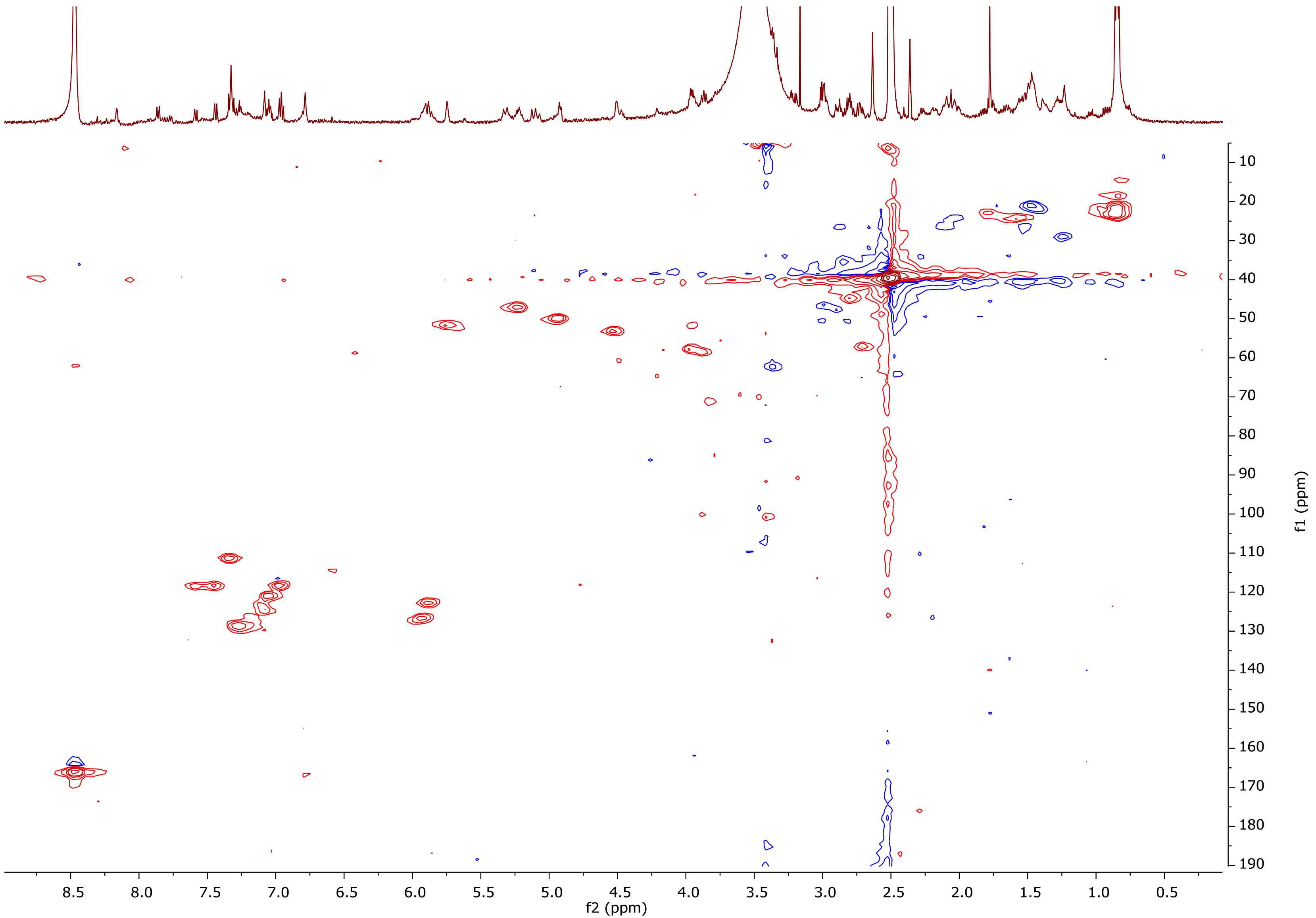

Figure S31. ^{1}H-^{13}C HSQC NMR spectrum of compound **6** (500/125 MHz, DMSO-d_6).

5. Analysis of MS/MS fragmentation pattern

Fragments resulting from peptide cleavage were calculated and matching fragments were highlighted in the spectra. Fragmentation sites along the peptide backbone were assigned using the Roepstorff and Fohlman[10] nomenclature. Given the MS/MS was obtained using Collision-Induced Dissociation (CID), *b*-ions and *y*-ions were predominantly detected.

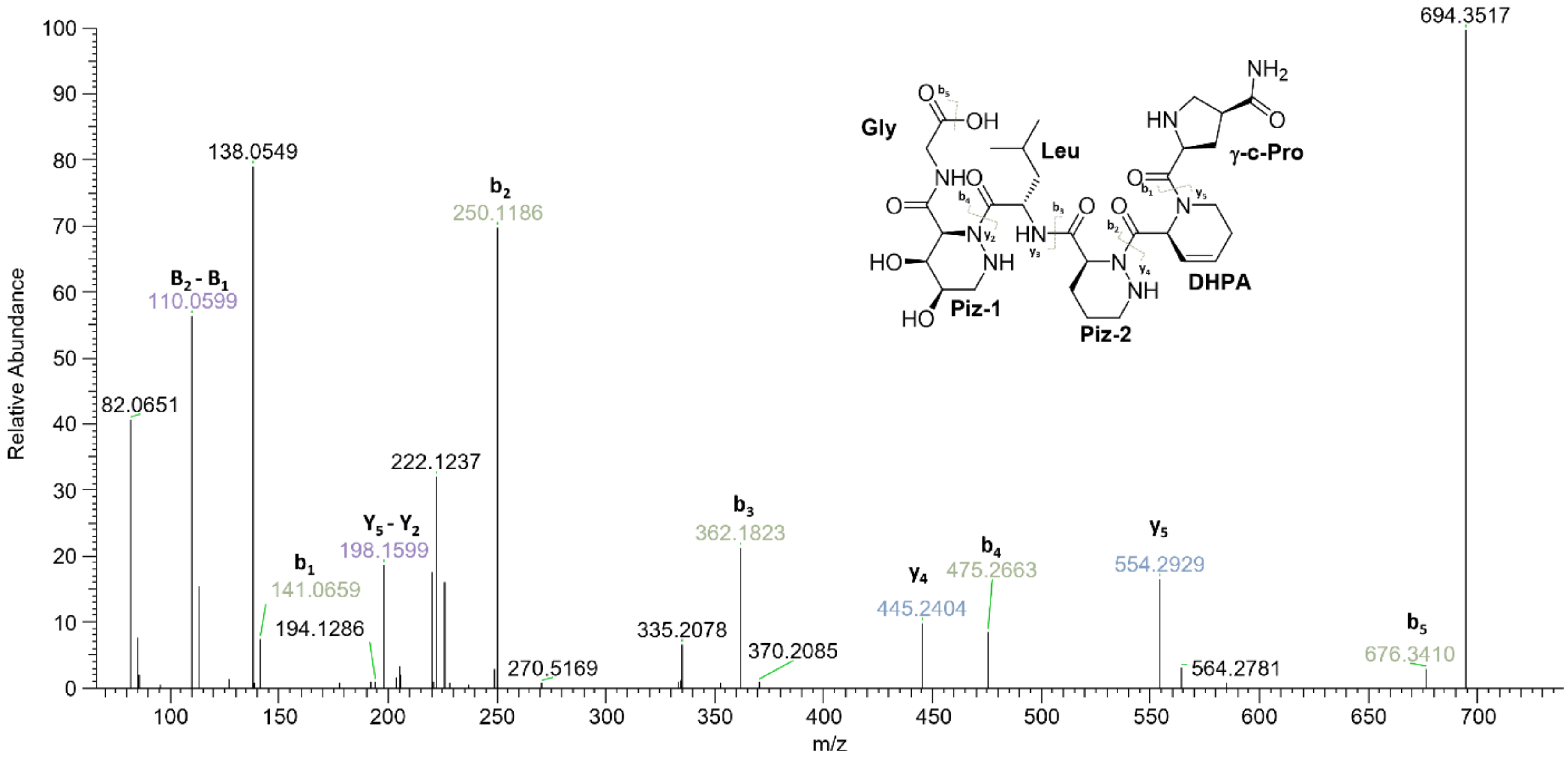

Figure S32. MS/MS spectrum of demiguisin A (**1**). Fragments resulting from peptide cleavage and matching calculated *m/z* values are highlighted.

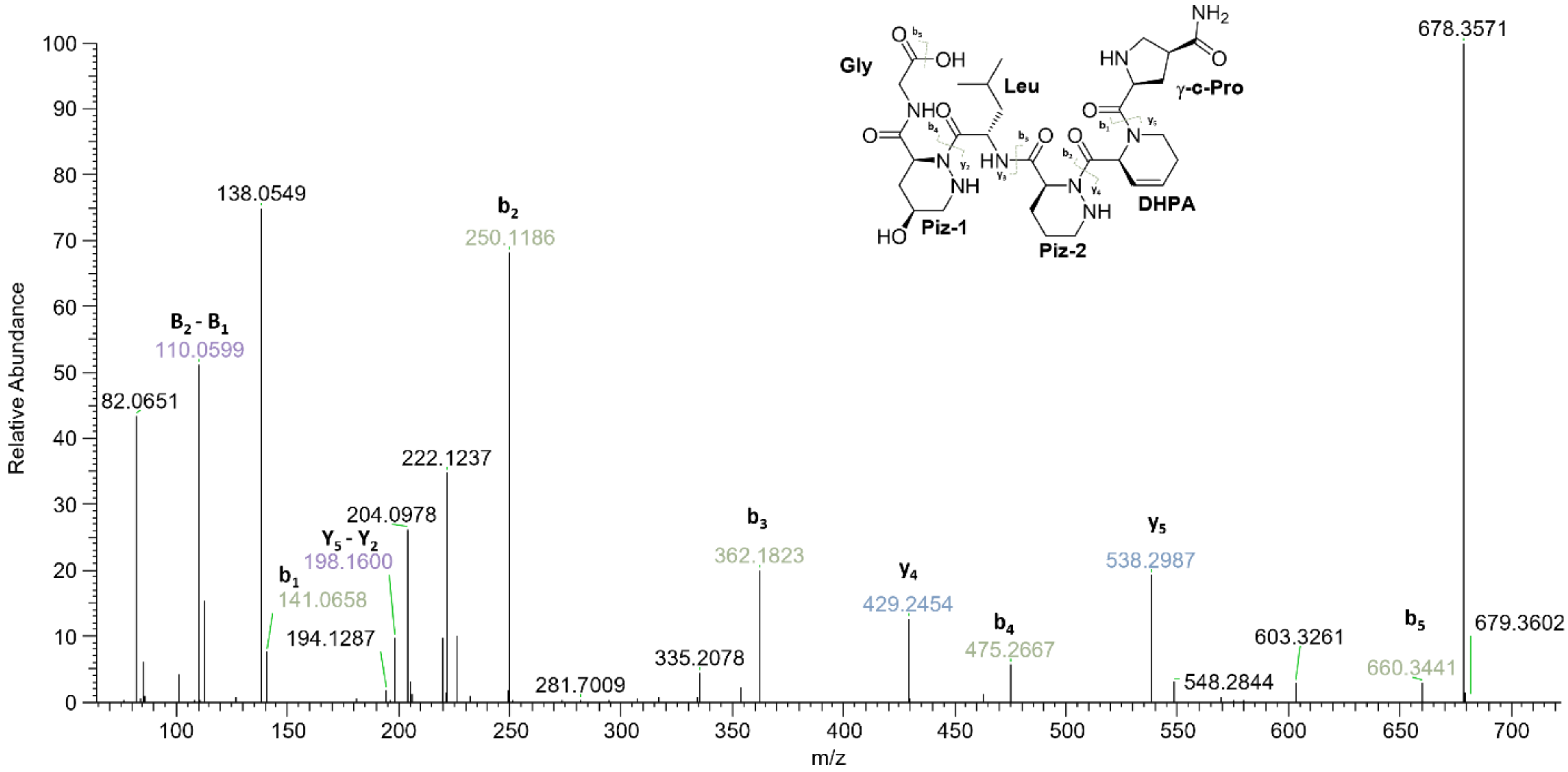

Figure S33. MS/MS spectrum of demiguisin B (**2**). Fragments resulting from peptide cleavage and matching calculated *m/z* values are highlighted.

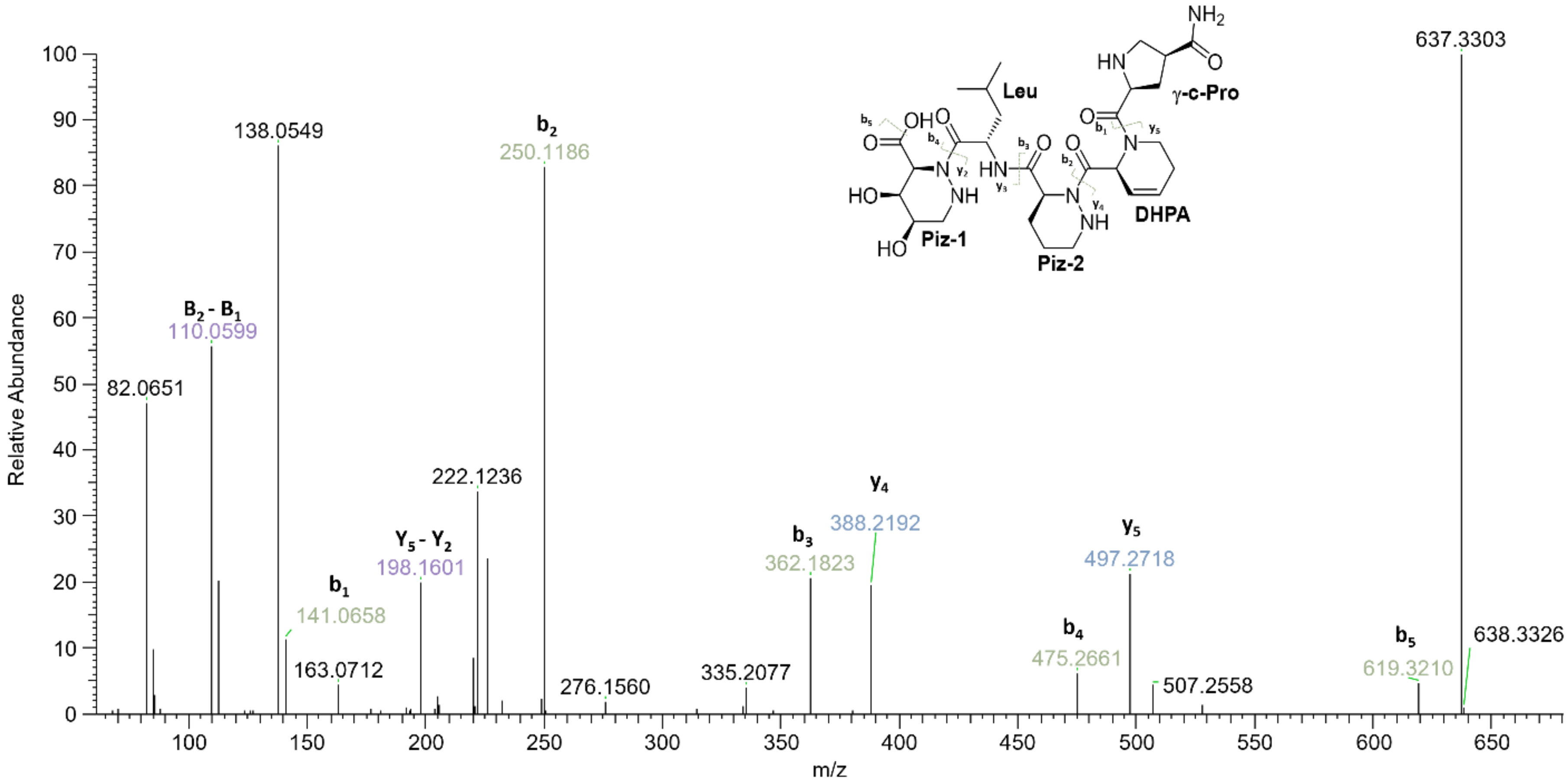

Figure S34. MS/MS spectrum of demiguisin C (**3**). Fragments resulting from peptide cleavage and matching calculated *m/z* values are highlighted.

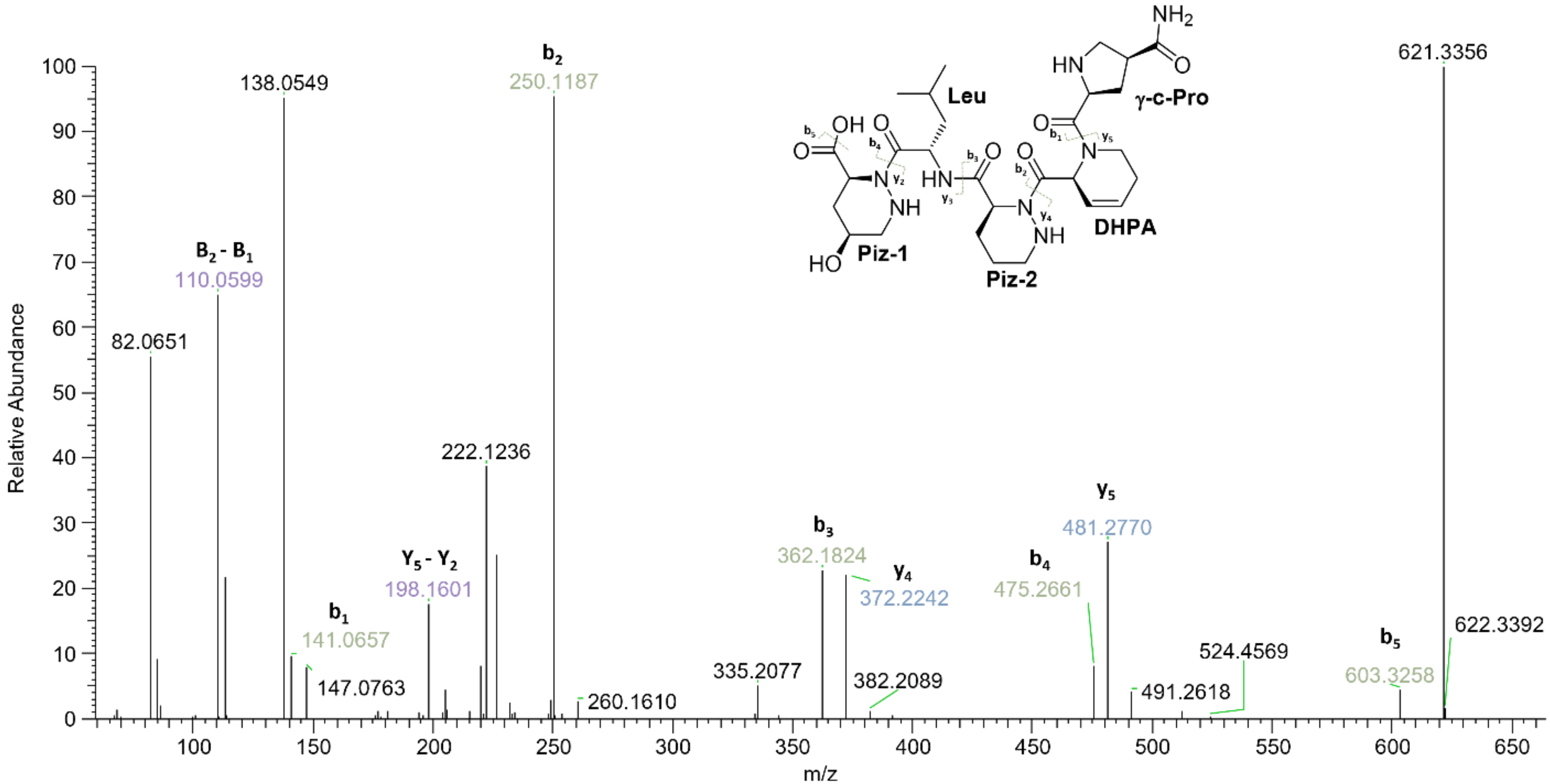

Figure S35. MS/MS spectrum of demiguisin D (**4**). Fragments resulting from peptide cleavage and matching calculated *m/z* values are highlighted.

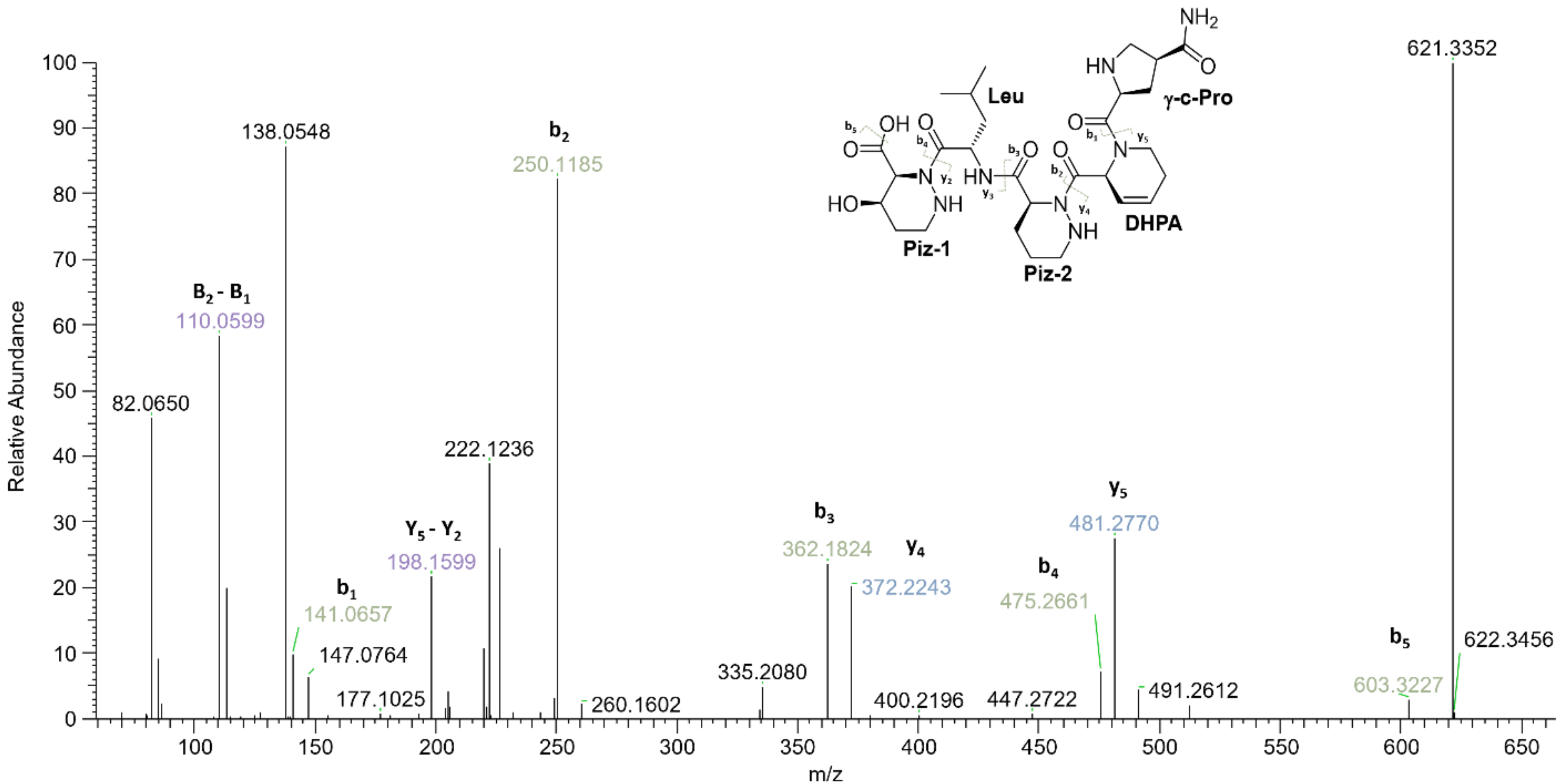

Figure S36. MS/MS spectrum of demiguisin E (**5**). Fragments resulting from peptide cleavage and matching calculated *m/z* values are highlighted.

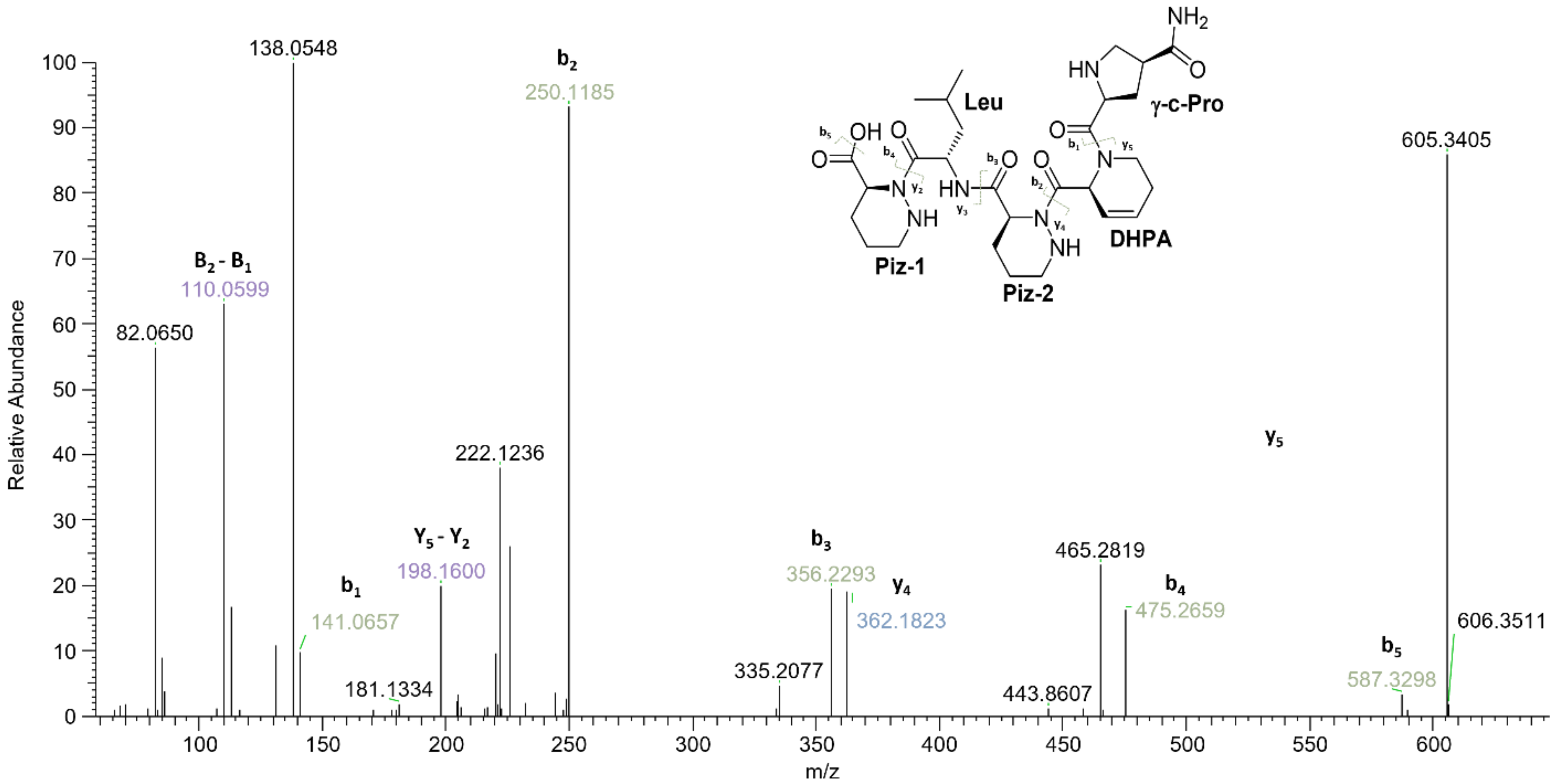

Figure S37. MS/MS spectrum of demiguisin F (**6**). Fragments resulting from peptide cleavage and matching calculated *m/z* values are highlighted.

Table S9. Summary of LC-HRMS/MS data of identified peptide compounds.

No.	LC-HRMS Rt (min)	[a]Experimental *m/z* [M+H]$^+$	[b]Theoretical *m/z* [M+H]$^+$	[c]Molecular formula	[d]Mass error (Δppm)	Amino acid sequence						Proposed demiguisin name
						1	2	3	4	5	6	
1	3.69	694.3510	694.3524	$C_{30}H_{47}N_9O_{10}$	2.02	COOH-Gly	β,γ-OH-Piz	Leu	Piz	DHPA	γ-c-Pro-NH_2	A
2	3.72	678.3571	678.3575	$C_{30}H_{47}N_9O_9$	1.77	COOH-Gly	γ-OH-Piz	Leu	Piz	DHPA	γ-c-Pro-NH_2	B
3	3.65	637.3299	637.3309	$C_{28}H_{44}N_8O_9$	1.57	-	COOH-β,γ-OH-Piz	Leu	Piz	DHPA	γ-c-Pro-NH_2	C
4	3.65	621.3348	621.3360	$C_{28}H_{45}N_8O_8$	1.93	-	COOH-γ-OH-Piz	Leu	Piz	DHPA	γ-c-Pro-NH_2	D
5	3.74	621.3378	621.3360	$C_{28}H_{45}N_8O_8$	2.90	-	COOH-β-OH-Piz	Leu	Piz	DHPA	γ-c-Pro-NH_2	E
6	4.34	605.3400	605.3411	$C_{28}H_{45}N_8O_7$	1.82	-	COOH-Piz	Leu	Piz	DHPA	γ-c-Pro-NH_2	F

[a] Experimental prononated M_r of the compound detected in the MS data on Freestyle version 1.8.

[b] Theoretical prononated M_r of the compound calculated using ChemDraw 22.2 software package.

[c] Theoretical chemical formula of compound calculated using ChemDraw 22.2 software package.

[d] Mass error in ppm = $\left(\frac{Theoretical\ Mr - Experimental\ Mr}{Theorectical\ Mr}\right) \times 10^6$

6. Biological activity

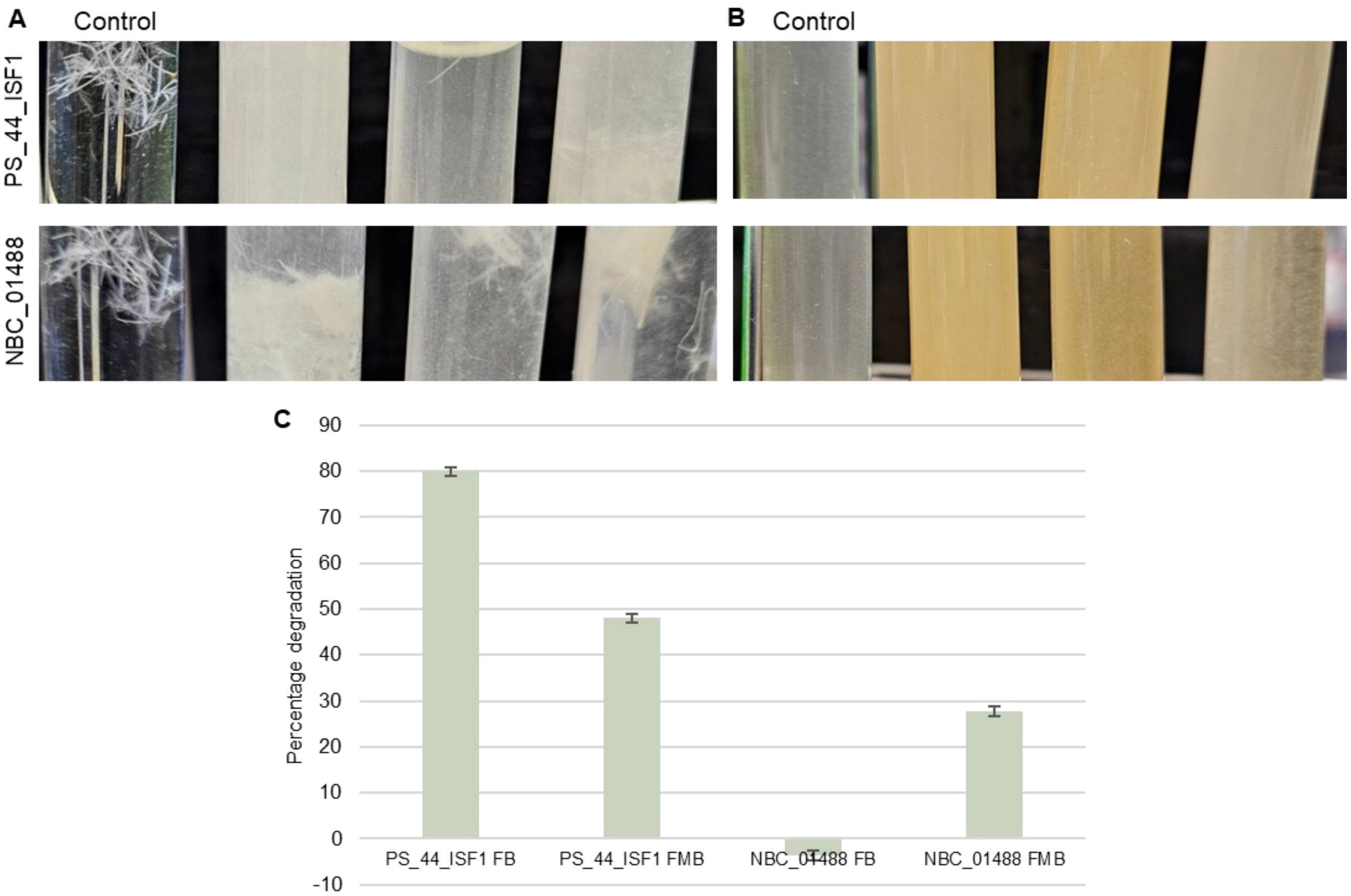

Figure S38. Keratinolytic activity of PS_44_ISF1 (top) and NBC_01488 (bottom) in (A) feather broth and (B) feather meal broth after 7 days. (C) Results of the keratin azure assay for the bird-derived PS_44_ISF1 strain and the soil-derived NBC_01488 strain.

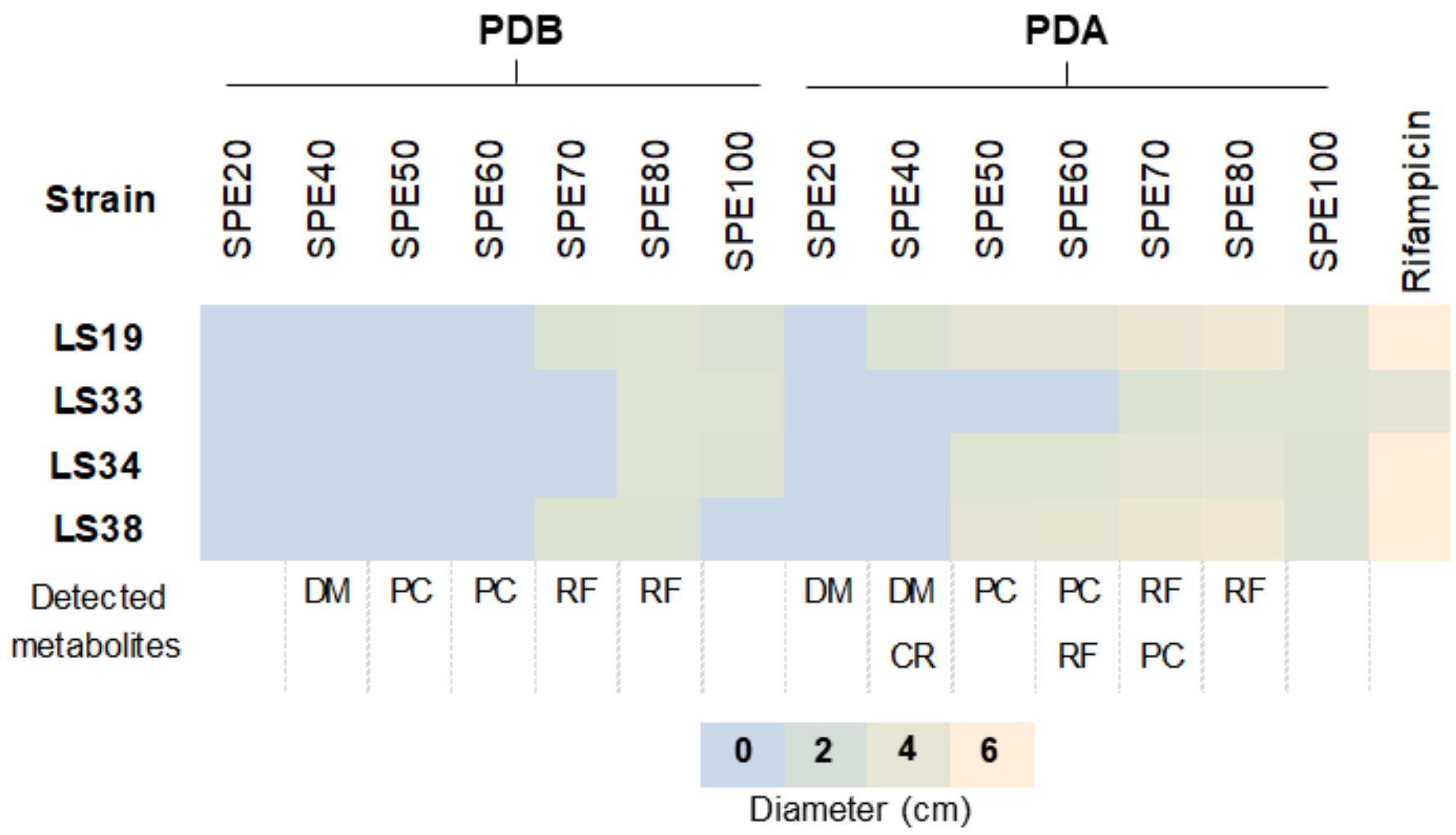

Figure S39. Heat-map summarizing antibacterial activity of fractions determined using a disc diffusion assay (SPE20–SPE100) obtained after PS_44_ISF1 was grown under different conditions (PDB and PDA) and tested against FDB (strains: LS19, LS33, LS34, and LS38), including rifampicin as a control. Shading reflects relative activity; MS-detected metabolite families are indicated below.

Table S10. Activity spectrum of the demiguisins against extended profiling against gram-positive and gram-negative bacteria, and a human cell line.

Indicator organism/ cell line	1	2	3	4	5	6
M. intracellulare ATCC13950	2	2	> 64	32	-	64
M. abscessus ATCC19977	> 64	> 64	> 64	> 64	> 64	> 64
M. avium ATCC700898 (MAC101)	> 64	> 64	> 64	> 64	> 64	> 64
M. tuberculosis H37Ra ATCC25177	64	16	> 64	> 64	> 64	> 64
M. marinum strain M (ATCC BAA-535)	> 64	> 64	> 64	> 64	> 64	> 64
M. luteus DSM 1970	> 64	> 64	> 64	> 64	> 64	> 64
S. aureus ATCC29213	> 64	> 64	> 64	> 64	> 64	> 64
E. coli ATCC 25922	> 64	> 64	> 64	> 64	> 64	> 64
E. coli K12 ΔtolC (tolC-deficient)	> 64	> 64	> 64	> 64	> 64	> 64
E. coli K12 ΔtolC (tolC-deficient) + PMBN	> 64	> 64	> 64	> 64	> 64	> 64
E. coli WO153, (AB1157, recJ asmB1, ΔtolC)	> 64	> 64	> 64	> 64	> 64	> 64
HepG2	>37	>37	>37	>37	>37	>37

Minimal inhibitory concentration (MIC) values are given in µg/mL. Cytotoxic activities are given as half maximal inhibitory concentrations (IC50) in µg/mL.

7. Biosynthetic pathway analysis

Table S11. Homologies and Putative Functions of Dem proteins in the *dem* BGC from *Amycolatopsis* sp. NBC_01488.

Name	Sequence Similarity on MiBIG (Protein)	Identity/ Similarity [%]	NCBI/ MiBIG Accession no.
**DemR1**	MiBIG: TetR family transcriptional regulator	33	ACY06306.1
**DemCYP1**	MiBIG: Cytochrome_P450	40	QDQ37897.1
**DemP1**	NRPS: A-PCP-C-PCP<br>MiBIG: Non-ribosomal peptide synthetase	42	AAG02349.1
**DemAA1**	MiBIG: Putative peptide monooxygenase	58	CAB53328.1
**DemAA2**	MiBIG: FMN-binding negative transcriptional regulator	54	WP_019634558.1
**DemP2**	NRPS: A-PCP<br>MiBIG: Non-ribosomal peptide synthetase	43	AJW65406.1
**DemAA3**	MiBIG: Putative F-420 dependent reductase	49	AOC89011.2
**DemAA4**	Pfam: DUF6002 family protein	-	-
**DemU1**	Pfam: GNAT family *N*-acetyltransferase	-	-
**DemAA5**	MiBIG: Asparagine synthase	53	OWA01599.1
**DemT1**	Pfam: DUF885 domain-containing protein	-	-
**DemP3**	NRPS: C<br>MiBIG: Non-ribosomal peptide synthetase	43	ACY06285.1
**DemT2**	MiBIG: MFS transporter	37	AZF85943.1
**DemP4**	MiBIG: MbtH-like protein	66	CCJ67644.1
**DemP5**	NRPS: C-A-PCP-C-A-PCP-C-A-PCP<br>MiBIG: Non-ribosomal peptide synthetase	43	ACY06285.1
**DemAA6**	MiBIG: L-lysin 2-aminotransferase	59	ALE27497.1
**DemAA7**	MiBIG: FAD-dependent oxidoreductase	24	AFW04572.1
**DemAA8**	MiBIG: Ornithine cyclodeaminase	46	QEO74901.1
**DemP6**	NRPS: TE<br>MiBIG: Type II thioesterase	40	AHB82050.1
**DemAA9a**	MiBIG: Decarboxylase subunit	51	AEO99210.1
**DemAA9a**	MiBIG: Decarboxylase subunit	46	AEO99211.1
**DemP7**	NRPS: A<br>MiBIG: Non-ribosomal peptide synthetase	42	AHB82072.1
**DemU2**	Hypothetical protein	-	-
**DemR3**	MiBIG: Transcriptional regulator	45	AAM78000.1
**DemU3**	Pfam: Carbonic anhydrase	-	-

Table S12. Substrate specificities of adenylation domains encoded within the *dem* and *dmg* pathways for demiguisin in NBC_01488 and PS_44_ISF1 strains.

Domain	NBC_01488		PS_44_ISF1	
	PRISM4 (score)	PARAS	PRISM4 (score)	PARAS
Dem/DmgP1-A1	Pro (357)	Pro (0.154)	Pip (360.3)	Pro (0.096)
Dem/DmgP2-A1	Pip (357.8)	Pro (0.538)	Phe (362.6)	Pro (0.284)
Dem/DmgP5-A1	Pro (494.6)	Pro (0.516)	Pro (482.6)	Pro (0.373)
Dem/DmgP5-A2	Pip (333.3)	Pro (0.151)	Ser (326.5)	Pro (0.094)
Dem/DmgP5-A3	Phe (458.5)	Leu (0.923)	Phe (404.5)	Leu (0.788)
Dem/DmgP7-A1	Gly (268.5)	Gly (0.557)	Gly (240.5)	Gly (0.415)

[a]Stachelhaus codes were predicted on antiSMASH version 8.0.4 according to Stachelhaus et al.[11]

Table S13. Sequence similarity and identity of cytochrome P450 monooxygenases identified in the dem BGC (demCYP450) against other CYP450 protein sequences known to hydroxylate Piz (determined using BLASTp).

Protein	Query coverage	E value	Percentage identity
Dmg_CYP450	100%	0.0	90.20%
PlyA_CYP450	91%	2e-45	32.64%
HmtN_CYP450	83%	3e-39	31.69%

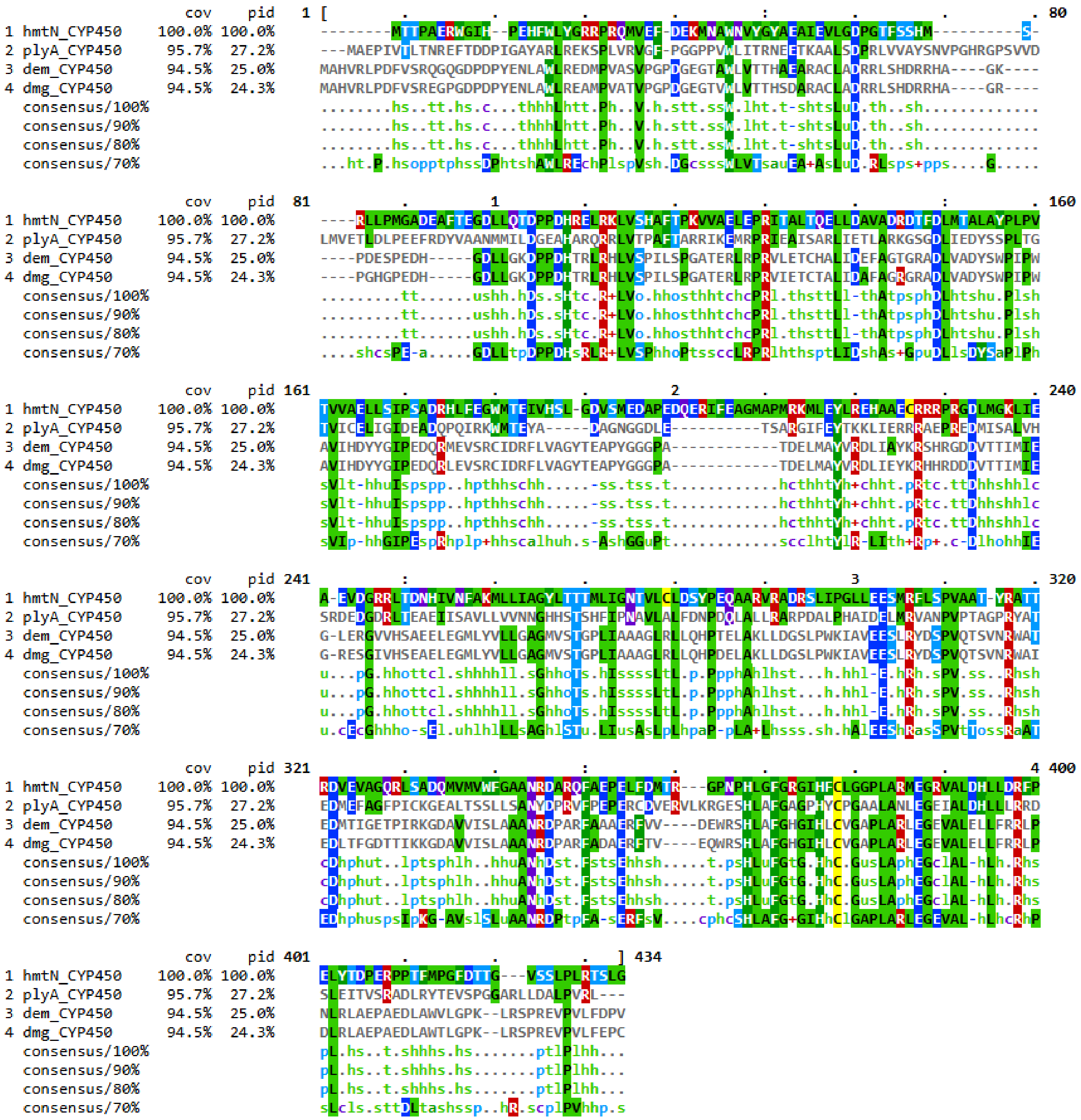

Figure S40. Sequence alignments of DemCYP450 with DmgCYP450, PlyA_CYP450 and HmtN_CYP450 protein sequences using the Clustal Omega multiple sequence alignment tool.

8. References

1. Núñez, I.; Matute, T. M9 minimal medium. *Protocols* **2024**. DOI: dx.doi.org/10.17504/protocols.io.8epv5x5j4g1b/v1
2. Lin, X.; Lee, C.G.; Casale, E.S.; Shih, J.C. Purification and characterization of a keratinase from a feather-degrading *Bacillus licheniformis* strain. *Appl. Environ. Microbiol.* **1992**, *58* (10), 3271-3275. DOI: 0099-2240/92/103271-05$02.00/0
3. Seibel, E.; Um, S.; Bodawatta, K.H.; Komor, A.J.; Decker, T.; Fricke, J.; Murphy, R.; Maiah, G.; Iova, B.; Maus, H.; Schirmeister, T. Bacteria from the *Amycolatopsis* genus associated with a toxic bird secrete protective secondary metabolites. *Nat. Commun.* **2024**, *15* (1), 8524, DOI: 10.1038/s41467-024-52316-3
4. Jørgensen, T.S.; Mohite, O.S.; Sterndorff, E.B.; Alvarez-Arevalo, M.; Blin, K.; Booth, T.J.; Charusanti, P.; Faurdal, D.; Hansen, T.Ø.; Nuhamunada, M.; Mourched, A.S. A treasure trove of 1034 actinomycete genomes. *Nucl. Acids Res.* **2024**, *52* (13), 7487-7503. DOI: 10.1093/nar/gkae523
5. Kim, K.H.; Ramadhar, T.R.; Beemelmanns, C.; Cao, S.; Poulsen, M.; Currie, C.R.; Clardy, J. Natalamycin A, an ansamycin from a termite-associated *Streptomyces* sp. *Chem. Sci.* **2014**, *5* (11), 4333-4338. DOI: 10.1039/C4SC01136H
6. Carlsohn, M.R.; Groth, I.; Tan, G.Y.A.; Schütze, B.; Saluz, H.P.; Munder, T.; Yang, J.; Wink, J.; Goodfellow, M. *Amycolatopsis saalfeldensis* sp. nov., a novel actinomycete isolated from a medieval alum slate mine. *Int. J. Syst. Evol. Microbiol.* **2007**, *57* (7), 1640-1646. DOI: 10.1099/ijs.0.64903-0
7. Decker, T.; Kochems, R.; de Kruijff, M.; Kushnareva, A.; Monisha Krishnan, M.; Beemelmanns, C. (unpublished results). Phylogenomics and Activity-Guided Discovery of Natural Products from *Rhodococcus* spp. of Zoo Animal Dung Microbiomes.
8. Schweizer, L.; Eckart; K. A.; Kochems, R., de Kruijff, M.; Bodawatta, K.H.; Jønsson K.A.; Beemelmanns, C. (unpublished results). Toxic birds and their protective symbionts:OMICs-driven analysis of uropygial gland microbiome for novel anti-infective discovery
9. Beemelmanns, C.; Ramadhar, T.R.; Kim, K.H.; Klassen, J.L.; Cao, S.; Wyche, T.P.; Hou, Y.; Poulsen, M.; Bugni, T.S.; Currie, C.R.; Clardy, J. Macrotermycins A–D, glycosylated macrolactams from a termite-associated *Amycolatopsis* sp. M39. *Org. Lett.* **2017**, *19* (5), 1000-1003. DOI: 10.1021/acs.orglett.6b03831
10. Roepstorff, P.; Fohlman, J. Proposal for a common nomenclature for sequence ions in mass spectra of peptides. *Biomed. Mass Spectrom.* **1984**, *11*, 601. DOI: 10.1002/bms.1200111109
11. Stachelhaus, T.; Mootz, H.D.; Marahiel, M.A.; 1999. The specificity-conferring code of adenylation domains in nonribosomal peptide synthetases. *Chem. Biol.* **1999**, *6* (8), 493-505.
