# Supplementary material for: Targeted pathway mining reveals novel modified hexapeptides from the demiguisin family
Source: Commun Chem. 2026 Oct 17;9:327. doi: 10.1038/s42004-026-02250-w (PMC13676617; doi:10.1038/s42004-026-02250-w)
Supplement: Supplementary file 3 [file 42004_2026_2250_MOESM3_ESM.pdf]

Description of Additional Supplementary files

File: Supplementary Data 1

Description: Keratin degradation assay measuring the absorbance of keratin azure at 595 nm.

File: Supplementary Data 2:

Description: Relative abundance of compounds **1 – 6** when *Amycolatopsis* sp. PS_44_ISF1 and NBC_01488 were grown in feather broth/agar and feather meal broth/agar over 7,10 or 14 days.

File: Supplementary Data 3:

Description: Relative abundance of compounds **1 – 6** when *Amycolatopsis* sp. PS_44_ISF1 and NBC_01488 were grown in differing culture media (i.e., PDA, PDB or M9).

File: Supplementary Data 4:

Description: Relative abundance of compounds **1 – 6** when *Amycolatopsis* sp. PS_44_ISF1 and NBC_01488 were grown in PDA over 4, 7 and 10 days.

File: Supplementary Data 5:

Description: Relative abundance of compounds **1 – 6** when *Amycolatopsis* sp. PS_44_ISF1 and NBC_01488 were grown in PDA in co-culture with *Kocuria* sp. LS23, *Streptomyces* sp. M56 and *Rhodococcus* sp. Zoo49.
